# Integration of Wnt-inhibitory activity and structural novelty scoring results to uncover novel bioactive natural products: new Bicyclo[3.3.1]non-3-ene-2,9-diones from the leaves of *Hymenocardia punctata*

**DOI:** 10.3389/fchem.2024.1371982

**Published:** 2024-04-04

**Authors:** Luis-Manuel Quiros-Guerrero, Laurence Marcourt, Nathareen Chaiwangrach, Alexey Koval, Emerson Ferreira Queiroz, Bruno David, Antonio Grondin, Vladimir L. Katanaev, Jean-Luc Wolfender

**Affiliations:** ^1^ Institute of Pharmaceutical Sciences of Western Switzerland, University of Geneva, Centre Médical Universitaire, Geneva, Switzerland; ^2^ School of Pharmaceutical Sciences, University of Geneva, Centre Médical Universitaire, Geneva, Switzerland; ^3^ Centre of Excellence in Cannabis Research, Department of Pharmaceutical Chemistry and Pharmacognosy, Faculty of Pharmaceutical Sciences, Naresuan University, Phitsanulok, Thailand; ^4^ Department of Cell Physiology and Metabolism, Translational Research Centre in Oncohaematology, Faculty of Medicine, Geneva, Switzerland; ^5^ Green Mission Department, Herbal Products Laboratory, Pierre Fabre Research Institute, Toulouse, France; ^6^ School of Medicine and Life Sciences, Far Eastern Federal University, Vladivostok, Russia

**Keywords:** natural products, Wnt-pathway, Wnt-pathway modulators, structural novelty discovery, ‘*Inventa*’ scoring, *Hymenocardia punctata*, metabolomics (LC-MS), structural elucidation

## Abstract

In natural products (NPs) research, methods for the efficient prioritization of natural extracts (NEs) are key for discovering novel bioactive NPs. In this study a biodiverse collection of 1,600 NEs, previously analyzed by UHPLC-HRMS^2^ metabolite profiling was screened for Wnt pathway regulation. The results of the biological screening drove the selection of a subset of 30 non-toxic NEs with an inhibitory IC_50_ ≤ 5 *μ*g/mL. To increase the chance of finding structurally novel bioactive NPs, *Inventa*, a computational tool for automated scoring of NEs based on structural novelty was used to mine the HRMS^2^ analysis and dereplication results. After this, four out of the 30 bioactive NEs were shortlisted by this approach. The most promising sample was the ethyl acetate extract of the leaves of *Hymenocardia punctata* (Phyllanthaceae). Further phytochemical investigations of this species resulted in the isolation of three known prenylated flavones (**3**, **5**, **7**) and ten novel bicyclo[3.3.1]non-3-ene-2,9-diones (**1**, **2**, **4**, **6**, **8**–**13**), named *Hymenotamayonins*. Assessment of the Wnt inhibitory activity of these compounds revealed that two prenylated flavones and three novel bicyclic compounds showed interesting activity without apparent cytotoxicity. This study highlights the potential of combining *Inventa*’s structural novelty scores with biological screening results to effectively discover novel bioactive NPs in large NE collections.

## Introduction

Nature is a valuable source of chemical diversity, offering a wide range of molecules with therapeutic properties (Newman and Cragg, 2020). Plants serve as important reservoirs of bioactive natural products (NPs) that have been utilized for medicinal purposes for centuries. NPs often exhibit complex chemical structures due to evolutionary processes that enable them to interact with biological targets in precise ways (Feher and Schmidt, 2003; Atanasov et al., 2021). These characteristics are challenging to replicate synthetically, making NPs exceptionally suitable as starting points for drug development (Clark, 1996; Dias et al., 2012; Allard et al., 2023). Natural extracts (NEs) from plant origin possess a vast chemical diversity of NPs, positioning them as highly promising assets for the exploration and advancement of novel therapeutic agents. Although to date, only about *c.a*. 20% of plant species have been investigated, finding novel or rare structural scaffolds is becoming increasingly difficult. This challenge arises because species that are taxonomically related tend to biosynthesize similar constituents (David et al., 2015).

The most common approaches used for the selection of NEs prior to *in-depth* phytochemical studies include high-throughput bioactivity screening, traditional use of given medicinal plants, and literature reports (Hostettmann and Terreaux, 2000; Sarker et al., 2005; Sarker and Nahar, 2012). The identification of the active principles is classically performed by bio guided isolation. This strategy is resource-intensive and time-consuming due to the need for multiple rounds of fractionation and bioassays. There is also a risk of bioactivity lost during the isolation process while other concerns include false positives, selectivity issues in bioassays, and missing synergistic effects (Pieters and Vlietinck, 2005; Hamburger, 2019; Najmi et al., 2022). To overcome certain limitations and anticipate the chances to find bioactive NPs of interest, strategies like structural dereplication and extensive metabolites annotations through metabolomics are increasingly being integrated early in research workflows (Olivon et al., 2017; Caesar et al., 2021).

Early structural identifications of NPs in NEs can assist researchers in avoiding reported active NPs or efficiently searching for analogs of previously reported bioactive NPs (Hubert et al., 2017; Selegato et al., 2023). With the advancement of computational annotations methods throughput in metabolomics, it is now possible to evaluate the chemical space of large NEs collections (Gaudry et al., 2023). This information can be used to prioritize samples in the search for structurally novel NPs (Quiros-Guerrero et al., 2022).

To automatedly mine the large amount of metabolite profiling data and make use of prior pharmacognosy knowledge we recently introduced *Inventa* (Quiros-Guerrero et al., 2022), a metabolomics bioinformatic workflow designed to streamline the NEs selection process. Its primary objective is to pinpoint NEs with a heightened probability of containing structurally novel NPs within NEs collections, that have undergone untargeted UHPLC-HRMS^2^ metabolite profiling. *Inventa* follows a structured process and takes as input the results from the MZmine data processing (Schmid et al., 2023), the subsequent MS^2^ spectral data organization using Featured-Based Molecular Networking (FBMN) (Nothias et al., 2020), and the MS^2^ spectra annotation from advanced computational methods like TIMA (Allard et al., 2016; Rutz et al., 2019), and SIRUS (Dührkop et al., 2019). The annotation results of the features [a peak with an *m/z* value at a given retention time (RT)] detected in the samples include molecular formulas, chemical classes based on NPClassifier (Dührkop et al., 2021; Kim et al., 2021), and structural candidates (Dührkop et al., 2015; Cabral et al., 2016). It integrates previous literature reports for the considered taxon by conducting automated searches in the LOTUS initiative (Rutz et al., 2022), where NP structure occurrences are catalogued in their respective source organisms. Additionally, it exploits the MEMO (Gaudry et al., 2022) spectral fingerprints to evaluate the spectral diversity exhibited by a particular sample within a set of NEs. Based on all these data, *Inventa* calculates four individual component scores: the proportion of annotated features in each NE, the specificity of these features within a NEs data set, the number of reported structures in the NE taxon and the spectral divergence of the individual NE within the data set. It provides a combined score (*Priority Score*, PS) that enables the prioritization of NEs based on their potential for containing structurally novel NPs. In this study we intend to evaluate how *Inventa* can be combined with bioactivity screening results to highlight structurally novel bioactive NPs capable of regulating the Wnt signaling pathway.

The Wnt signaling pathway (Q155769) is critical in several biological processes like embryonic development, tissue homeostasis, and cellular proliferation (Blagodatski et al., 2020; Boudou et al., 2022; Liu et al., 2022). However, when dysregulated, it has been associated with several disorders, including cancer (Shaw et al., 2019b; Lim et al., 2021; Jiang et al., 2022), Alzheimer’s (Inestrosa et al., 2012), and osteoporosis (Houschyar et al., 2018; Lojk and Marc, 2021). Many of the current cancer treatments affect rapidly dividing cells resulting in notable side effects since these cells are essential for tissue maintenance in adults. A more targeted and specific approach, with fewer side effects, may be possible by focusing on targeting the Wnt signaling pathway exclusively in the cancer cells (Shaw et al., 2019b). Several NPs from diverse plant species have been reported to have some activity over the Wnt-signaling pathway through disruption of the Wnt/*β*-catenin cascade (Pooja and Karunagaran, 2014; Gu et al., 2019). Thus, the discovery of NPs capable of inhibiting or regulating the Wnt-signaling pathway has become a topic of significant interest in drug discovery programs (Fuentes et al., 2015; Nusse and Clevers, 2017).

The collection of NEs used for the Wnt-pathway regulation screening consists in a subset of 1,600 NEs from the Pierre Fabre Laboratories (PFL) Library that were previously analyzed by massive UHPLC-HRMS^2^ metabolite profiling, and different annotations workflows were applied. The set data was publicly disclosed allowing researchers to explore a wide range of chemical compositions across different plant species (Allard et al., 2023). The NEs were generated directly from the plant material by maceration with ethyl acetate, followed by SiO_2_-SPE filtration. This method was optimized for the recovery of middle polarity compounds, which is crucial for the objectives of the HTS program conducted by PFL. The samples were prepared in DMSO at a concentration of 5 mg/mL (Allard et al., 2023). This set of 1,600 NEs has been exploited for the development of bioinformatics tools (Gaudry et al., 2022; Gaudry et al., 2023).

In the search for structurally novel bioactive NPs from plants, we sought to investigate the UHPLC-HRMS^2^ metabolite profiling and Wnt-pathway regulation screening results for this set of 1,600 NEs. Then, to increase the chances of selecting active NEs containing novel NPs, *Inventa* was used to calculate priority scores for structural novelty. The combination of both information, the screening results, and *Inventa*’s scores highlighted several bioactive NEs with a high potential of containing structurally novel NPs.

## Results and discussion

### Selection of promising NEs by combining bioactivity results and structural novelty scores

The same samples used for the UHPLC-HRMS^2^ metabolite profiling previously described by Allard et al. (2023) were screened for the presence of compounds with a potential Wnt-regulatory activity. The screening experimental design used the BT-20 triple-negative breast cancer cell line (TNBC), stably transfected with the TopFlash reporter construct, and sensitive to purified Wnt3a stimulation (Koval et al., 2014; Shaw et al., 2019a). The NEs were screened in single repeats at five different concentrations (50, 25, 12.5, 6, and 3 *μ*g/mL) and cytotoxicity was monitored at the same time. Given that the assay does not include positive control compounds, an NEs or compound is considered ‘toxic’ if its IC_50_ value against Renilla luciferase is less than 1.7 times the estimated TopFlash value. This indicates that any reduction observed in the TopFlash response is likely influenced by a significant toxic effect (Shaw et al., 2019a).

The results of the Wnt-regulatory bioactivity testing showed that out of the 1,600 NEs, 497 exhibited either Wnt-regulatory or cytotoxic activity. Among these active samples, 389 active NEs were classified into 148 NEs Wnt potentiators (79 NEs were non-toxic and 69 NEs had a toxicity IC_50_ > 50 *μg*/mL), and 241 NEs Wnt inhibitors (all non-toxic). The remaining 108 NEs were solely cytotoxic, with an IC_50_ value ranging between 0.30 *μg*/mL and 50 *μg*/mL.

Out of these 241 inhibitory NEs, 132 NEs showed a Wnt-inhibition IC_50_ < 50 *μg*/mL, with 53 NEs having a Wnt-inhibition IC_50_ < 10 *μg*/mL. Focusing on samples capable of inhibiting the Wnt pathway is essential for discovering NPs to treat diseases linked to the dysregulation of this pathway. Therefore, in this study, only inhibitory NEs were further considered.

The 241 NEs with Wnt inhibitory activities mentioned above comprised a total of 58 different botanical families, 97 different genera, and 105 unique species. Fabaceae (Q44448) is the most represented family with 14 samples, followed by Rubiaceae (Q156569) and Euphorbiaceae (Q156584) with nine and eight samples respectively. The most represented genus is *Pandanus* (Q471914, Pandanaceae Q736182) with five samples, followed by *Sambucus* (Q131448, Adoxaceae Q156677) with four samples, and *Elegia* (Q3007993, Restionaceae Q131501), *Baliospermum* (Q4850999, Euphorbiaceae Q156584), and *Dolichos* (Q526727, Fabaceae), each with three samples.

To further reduce the list only NEs with an inhibition IC_50_ below 5 *μg*/mL were considered. This reduced the 241 to 30 NEs comprising 25 different species from 17 different botanical families, with Fabaceae and Euphorbiaceae being the most prominent, each contributing 5 NEs. Within this reduced set, there were 23 different genera, with the majority represented by only 1 NE, except for *Elegia* with three samples, and *Pandanus*, *Euphorbia* (Q146567), *Ehretia* (Q276756), and *Baliospermum* with two samples each.

**FIGURE 1:**
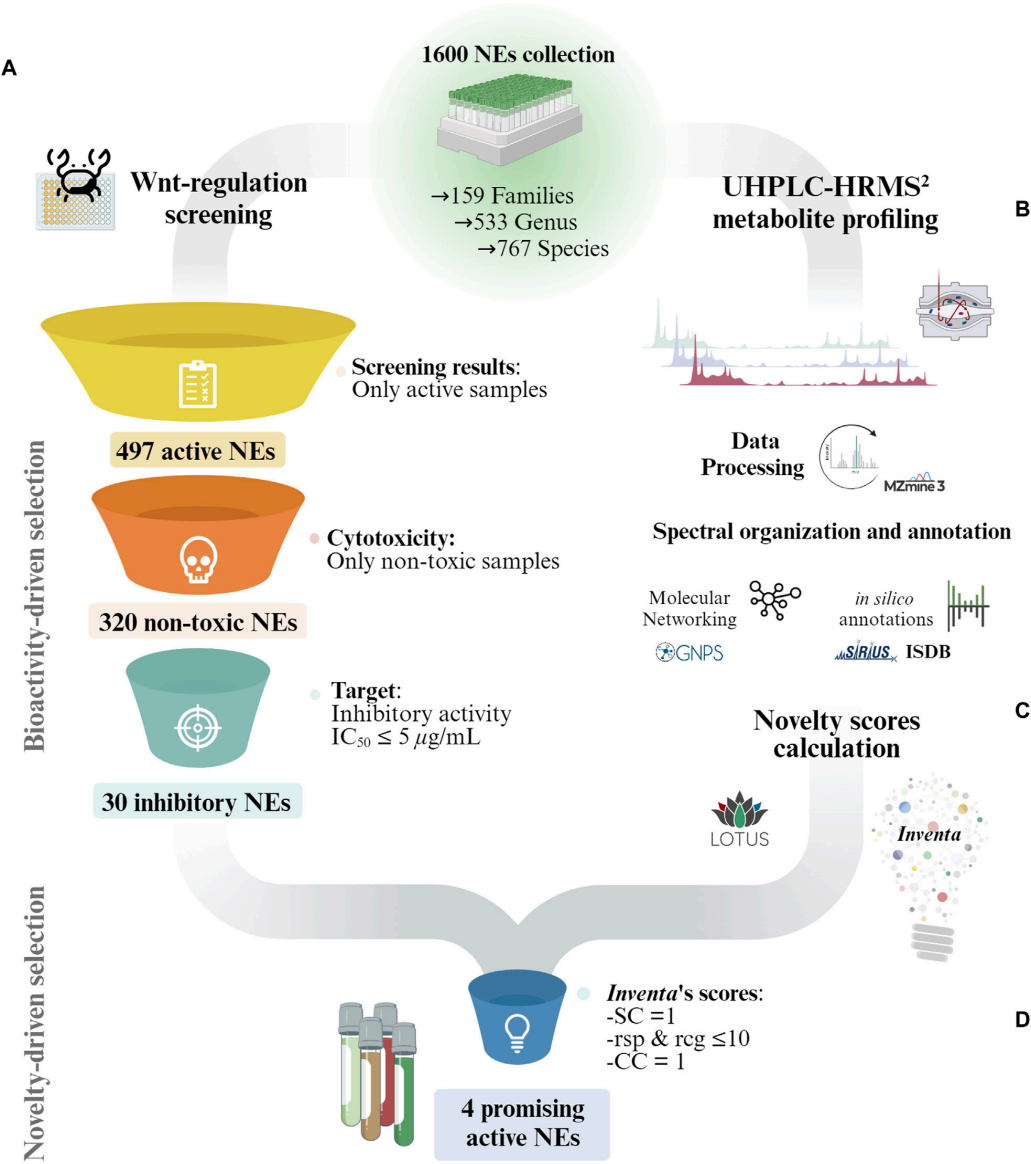
Overview of the general strategy for the selection of promising NEs for the discovery of structurally novel bioactive NPs in collections of NEs. **(A)** Bioactive-driven selection: The NEs collection is reduced according to the bioactivity screening results. In this study only non-toxic NEs with an Wnt inhibitory IC_50_ ≤ 5 *μ*g/mL were considered. **(B)** General overview of the UHPLC-HRMS^2^ profiling and annotation workflow used for the characterization of the 1,600 NEs collection by Allard et al. (2023). **(C)** The UHPLC-HRMS^2^ and annotations results for the entire 1,600 NEs collection were fed to *Inventa* to calculate the structural novelty scores. **(D)** Novelty-driven selection: Each component given by *Inventa* for the reduced set of NEs was individually assessed for a more precise selection. SC: Similarity Component; CC: Class Component; rsp: reported compounds in species; rsg: reported compounds in genus.

The biological screening results drove the first selection step, resulting in a subset of 30 non-toxic NEs with an inhibitory IC_50_ ≤ 5 *μ*g/mL (see Figure 1A). To further refine the selection, additional selection criteria based on UHPLC-HRMS^2^ metabolite profiling data were used. Specifically, *Inventa* scores, which evaluates the metabolites potential structural novelty within NEs (Quiros-Guerrero et al., 2022). These scores were calculated for the entire 1,600 NEs set using the positive ionization (PI) mode UHPLC-HRMS^2^ (Allard et al., 2023) (Figures 1.B,C). They were therefore not limited to the 30 active extracts alone, and thus better demonstrated their potential for holding new structures, since the reference sample set was much broader than that restricted by the biological activity filter.

The rough ranking based on PS significantly reduces the number of samples to consider which is important in large datasets. However, within the list of top-ranked samples, it is important to evaluate each parameter individually and, when possible, refine the literature search. This provides a better overview of the available data. The PS score enables to rapidly estimate the likeliness of a sample to contain potentially structurally novel NPs. This should not be interpreted as an absolute ranking. In this study, the focus shifted from the entire collection of 1,600 NEs to a much smaller subset of 30 active NEs. Instead of selecting these NEs based on their overall PS assigned by *Inventa*, a more meticulous approach was adopted. Each *Inventa*’s component for these NEs was individually assessed for a more precise selection.

The importance of the different novelty score components in the selection of NEs lies in its multifaceted approach to evaluating the structural richness and dissimilarities among samples. *Inventa* operates at two levels: first, by assessing individual features within each extract to gauge their specificity and annotation status, and second, by comparing the overall spectral space of each extract to measure dissimilarities in a sample set and potentially highlight NEs holding a pool of spectra correlated to a very specific metabolome. Subsequently, it integrates data from literature reports for the taxon, highlighting NEs potentially containing novel NPs (Quiros-Guerrero et al., 2022). The insights gained from these scores offer a thorough evaluation of the potential for extracts to contain structurally novel NPs. This comprehensive evaluation framework empowers researchers to pinpoint NEs with untapped metabolic potential, thereby facilitating the discovery of novel NPs with potential therapeutic applications.

First, to ensure that only NEs potentially holding specific constituents (specific pool of MS^2^ spectra), only those with a *Similarity Component* (SC) value of ‘1’ were further considered. This score highlights extracts containing metabolites whose MS^2^ spectra are significantly different from those of all 1,600 extracts in the data set. The SC employes the MEMO metric (Gaudry et al., 2022) to generate a matrix containing all MS^2^ information in the form of peaks and neutral losses (Huber et al., 2020; 2021) and automatic outlier detection machine learning algorithms to emphasize NEs that display substantial spectral dissimilarity (Quiros-Guerrero et al., 2022). Out of the 30 NEs only six remained after this filter.

Then, only NEs with a *Literature Component* (LC) value close to ‘1’ were selected. The value of LC reflects a rough estimation of the extent of the prior phytochemical knowledge for a given taxon (according to LOTUS). The closer to ‘1’, the fewer compounds have been reported at the species, genus, and family levels for a given sample*. Inventa* calculates the number of reported compounds for each species, genus, and family, and this data forms part of the final information provided for each sample. Based on this information, only NEs reporting less than 10 reported compounds in both genus and species levels were further considered (Figure 1D).

This reduced the 6 NEs to only 4 NEs with few compounds reported, since the species *Derris scandens* (Q15488445, Fabaceae) and *Iris lactea* (Q6747387, Iridaceae Q155941) presented over 100 and 400 compounds reported at the genus level respectively (see Wikidata Query results for genus *Derris* and *Iris*). Additionally, the remaining 4 NEs presented a *Class Component* (CC) of ‘1’. A CC value of one indicates that there are chemical classes proposed by CANOPUS (Dührkop et al., 2021) not yet reported for both the species and the genus (according to LOTUS). This suggests a high probability of potentially discovering unreported NPs within these NEs (Quiros-Guerrero et al., 2022).

The four highly active NEs remaining were: *Hymenocardia punctata* Wall. ex Lindl. (Q15514019, Phyllanthaceae Q133206), *Aporosa villosa* (Lindl.) Baill. (Q11128570, Euphorbiaceae Q156584), and 2 NEs of different plant parts of *Baliospermum sonalifolium* (Burm.) Suresh (Q15386102, Euphorbiaceae).

To further refine the selection process, a thorough complementary literature search was done on this final set of three species, considering all the possible botanical synonyms according to WFO Plant List. This search revealed no direct reports for the *Brosimum solanifolium* species. However, for one of its synonyms, *Baliospermum montanum* (Q3595677), the literature reports the presence of alkaloids, daphnanes, ingenanes, and phorbol esters (tiglianes Q27117179) like montanin (Q27107381) and baliospermin (Q27105913) (Seigler, 1994; Mali and Wadekar, 2008) with proven anticancer activity (Ogura et al., 1978). These metabolites were not accessible by LOTUS, so they were not considered in first instance. Since *B. solanifolium*, presents active reported NPs, both extracts were no further considered. For *A. villosa*, some reports already described various bioactivities of ethanolic extracts from different plant parts were reported (Srikrishna et al., 2008; Venkataraman et al., 2010; Nanna et al., 2021). Additionally, preliminary metabolite profiling indicated a high concentration of fatty acids. Therefore, this plant was not initially considered for further study.

In contrast, for *Hymenocardia punctata*, there were no existing reports on its chemical composition or bioactivity evaluation. This lack of information aligned with its initial *LC* score based on the Lotus. Consequently, the ethyl acetate extract of *H. punctata* leaves was identified as the most promising candidate for the discovery of novel NPs. This plant is a flowering shrub from the Phyllanthaceae family, found in Myanmar, Thailand, Laos, Cambodia, the Malay Peninsula, and Sumatra (van Welzen, 2016).

### Dereplication results overview for the ethyl acetate extract of *Hymenocardia punctata* leaves

**FIGURE 2:**
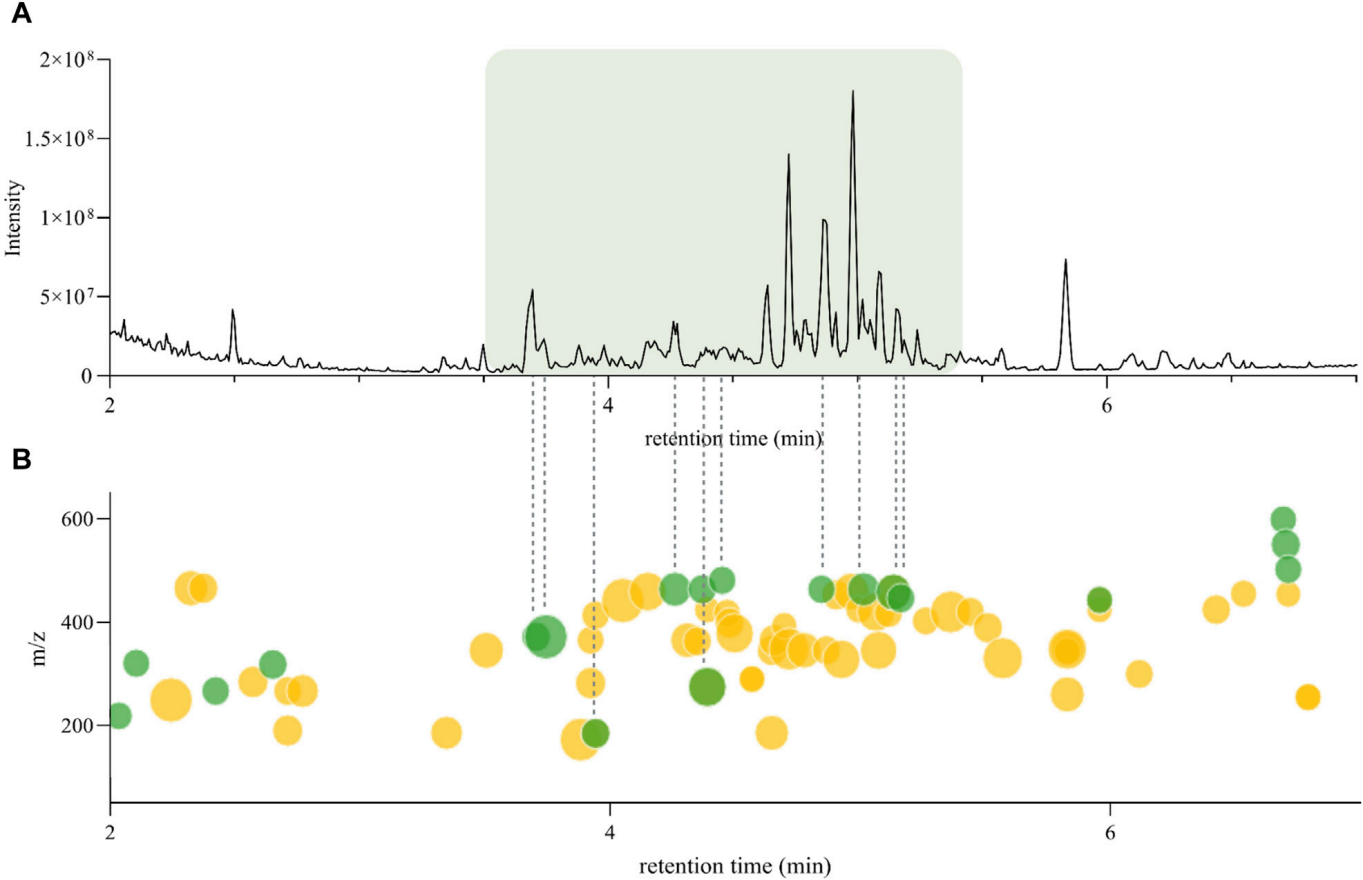
**(A)** Original UHPLC-HRMS^2^ chromatogram of *Hymenocardia punctata* leaves ethyl acetate extract from the 1,600 NEs collection. **(B)** *Inventa*’s ion map for the Original UHPLC-HRMS^2^ chromatogram of *Hymenocardia punctata* leaves ethyl acetate extract. Each dot represents one feature, the size is proportional to the intensity. The color shows the annotation status, green: unannotated, yellow: annotated (see interactive plot here).

According to *Inventa*’s results, the annotation rate for the *H. punctata* extract was notably high (*c.a*. 75%). To further explore the regions of the chromatogram that were annotated, the comparison between the original (PI) UHPLC-HRMS^2^ chromatogram (from the 1,600 NEs collection metabolite profiling) and the *Ionmap* generated by *Inventa* was carefully inspected (Figure 2). Upon examination of the SIRIUS and ISDB annotation results, as well as the outcomes of Ion Identity FBMN (II-FBMN, see Supplementary Figure S1, PDF version here) (Nothias et al., 2020; Schmid et al., 2021), it emerged clearly that the most intense features (Figure 2A), were not annotated (green dots on the *Ionmap* in Figure 2B).

**FIGURE 3:**
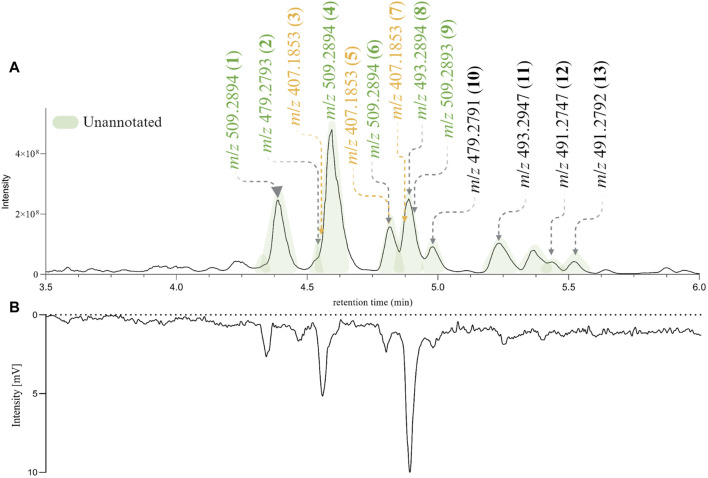
**(A)** PI UHPLC-HRMS^2^ chromatogram of *de novo Hymenocardia punctata* leaves ethyl acetate extract (HPE) between 3.5 min and 6 min. **(B)** Charge Aerosol Detector semiquantitative trace of HPE. The chromatographic peaks colored in green correspond to unannotated features after the dereplication process. The mass-to-charge ratio (*m/z*) of the most intense ions in the PI HRMS^2^ trace are shown. The *m/z* colored in green (unannotated features) and yellow (annotated features) found in the original HRMS^2^ profiling of *Hymenocardia punctata* (in the 1,600 NEs collection, Figure 2A).

For the following phytochemical studies, the leaves of *H. punctata* were subsequently extracted on a larger scale with hexane, ethyl acetate (HPE) and methanol. The HPE and HPM extracts underwent UHPLC-HRMS^2^ metabolite profiling. Additionally, a Charge Aerosol Detector (CAD) was used to obtain semiquantitative information (Ligor et al., 2013; Gamache, 2017). After careful composition assessment, only HPE was further considered. As shown in Figure 3A the features of interest were present and correlated with the major compounds in the extract according to the CAD chromatographic trace (Figure 3B).

The (PI) UHPLC-HRMS^2^ data from HPE was used to generate a new II-FBMN which confirmed most information obtained in the original extract from the 1,600 NEs collection. The most intense peaks were clustered together indicating their close structural relationship (II-FBMN, see Supplementary Figure S2, PDF version here). The chemical class and structural annotations obtained through GNPS, SIRIUS and CANOPUS (Dührkop et al., 2019; 2021) suggested that most compounds derived from the shikimate-phenylpropanoid and terpenoid pathways (refer to Treemap overview Supplementary Figure S3 -interactive plot visualization here-, and Supplementary Table S1).

Both the CAD and MS traces confirmed that the major constituents of HPE were not annotated. This, together with the novelty scores given by *Inventa*, confirmed that HPE is a promising extract for the search for new bioactive NPs.

### HPLC-based bioactivity profiling of the ethyl acetate extract of *Hymenocardia punctata* leaves

An HPLC-based bioactivity profiling (Hamburger, 2019) was carried out to establish a relationship between the major unannotated chromatographic peaks (potentially new NPs) and the observed bioactivity of HPE. A small amount of HPE (*c*.*a*. 10 mg) was fractionated by semi-preparative HPLC-UV under optimized chromatographic conditions. Column’s effluent was collected into a 96 deep-well plate and the Wnt-regulatory bioactivity of each dried micro-fraction was assessed. The HPLC based bioactivity profile confirmed that the bioactivity was mainly related to the major unannotated peaks (See Supplementary Figure S4).

### Isolation and *de novo* structural characterization of compounds from the *Hymenocardia punctata* leaves ethyl acetate extract

According to the dereplication results and the HPLC-based bioactivity profiling, the isolation efforts should be focused on the peaks with retention times between 3.5 and 6 min (see Figure 3). An *in-depth* phytochemical study of the HPE extract was carried out to corroborate the presence of structurally novel NPs and evaluate their Wnt regulatory activity. The chromatographic conditions used for the HPLC based bioactivity profiling were adapted to the flash chromatography scale using a geometrical gradient transfer (Guillarme et al., 2008). Several of the fractions obtained contained mixtures of compounds that were further separated by high resolution semi-preparative HPLC using dry-load injection (Queiroz et al., 2019).

**FIGURE 4:**
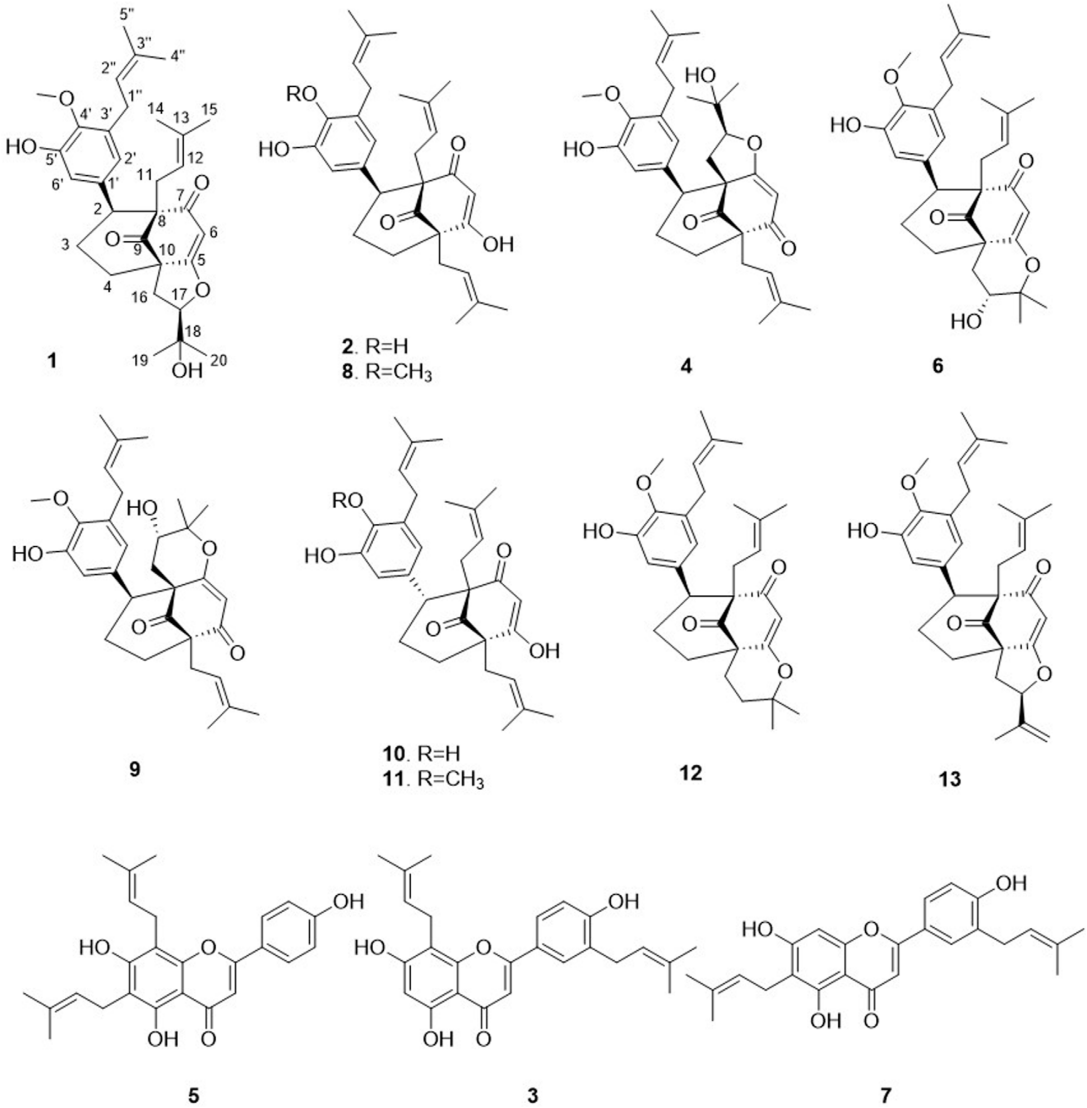
Structures of the isolated compounds from the ethyl acetate extract of *Hymenocardia punctata* leaves.

This approach allowed the successful isolation of thirteen compounds (**1**–**13**, see Figure 4). The structures of all the compounds were determined based on the NMR and HRMS analyses. Three of these compounds were identified as 3′,8-diprenylapigenin **3**) previously isolated from *Morus alba* (Q157307) (Dat et al., 2010), 6,8-diprenylapigenin **5**) previously isolated from *Glycyrrhiza inflata* (Q5572787) (Lin et al., 2017), and 3′,6-diprenylapigenin **7**) previously isolated from *Glycyrrhiza uralensis* (Q1196166) (Fukai et al., 1991). These compounds (**3**, **5**, **7**) were annotated as prenylated isoflavones by GNPS and Sirius. The other ten molecules corresponded to new compounds and were named *Hymenotamayonins* A-J (**1**, **2**, **4**, **6**, **8**–**13**), their full *de novo* structure identification is detailed below.

The relative retention time in the PI HRMS^2^ chromatogram is depicted in Figure 3 (numbers in parenthesis). The 13 compounds were recovered in sufficient amounts to allow full structural characterization and assessment of biological activity from only 55 g of dried plant material.

**FIGURE 5:**
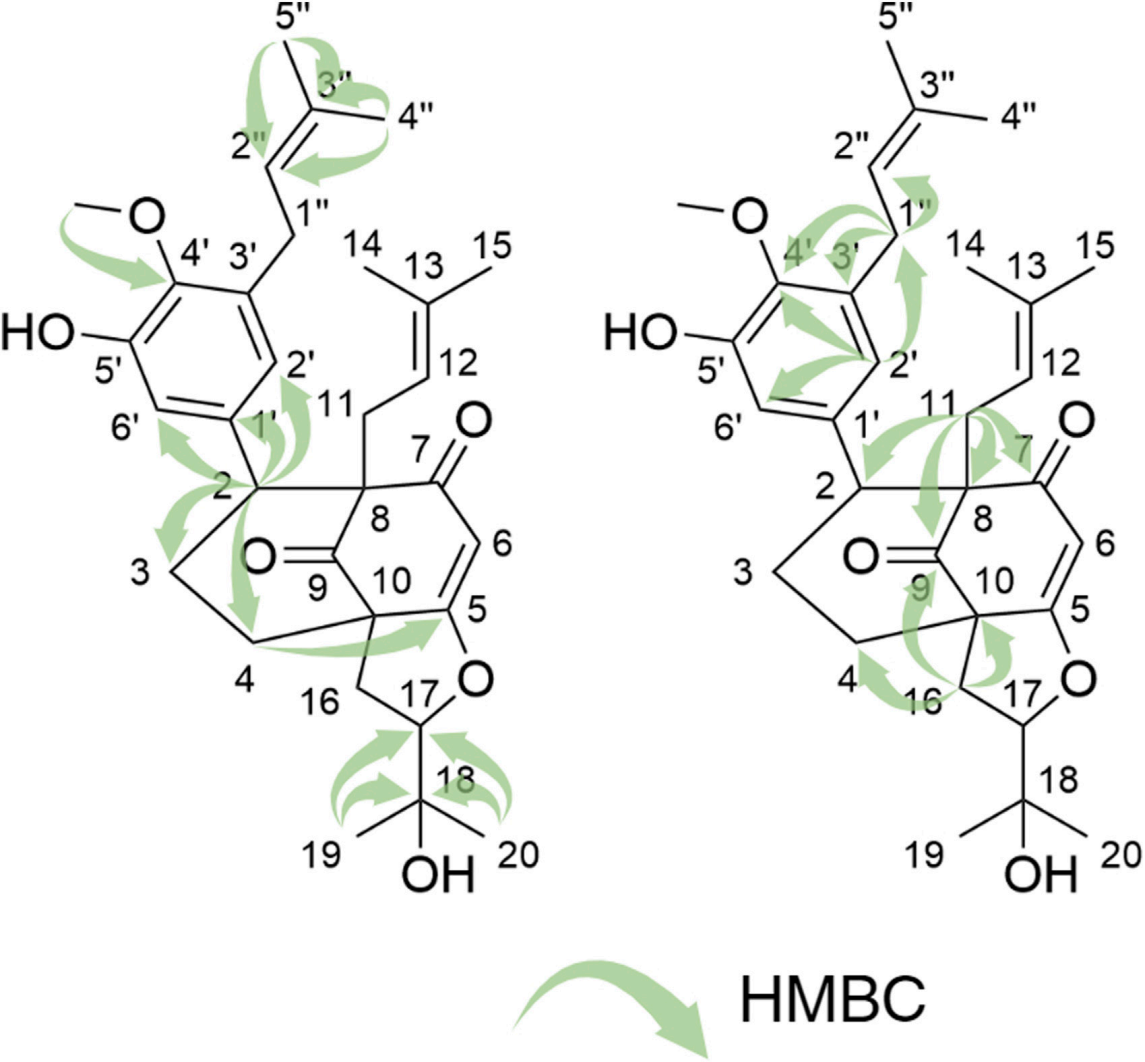
Key HMBC correlation for compound **1**.

**FIGURE 6:**
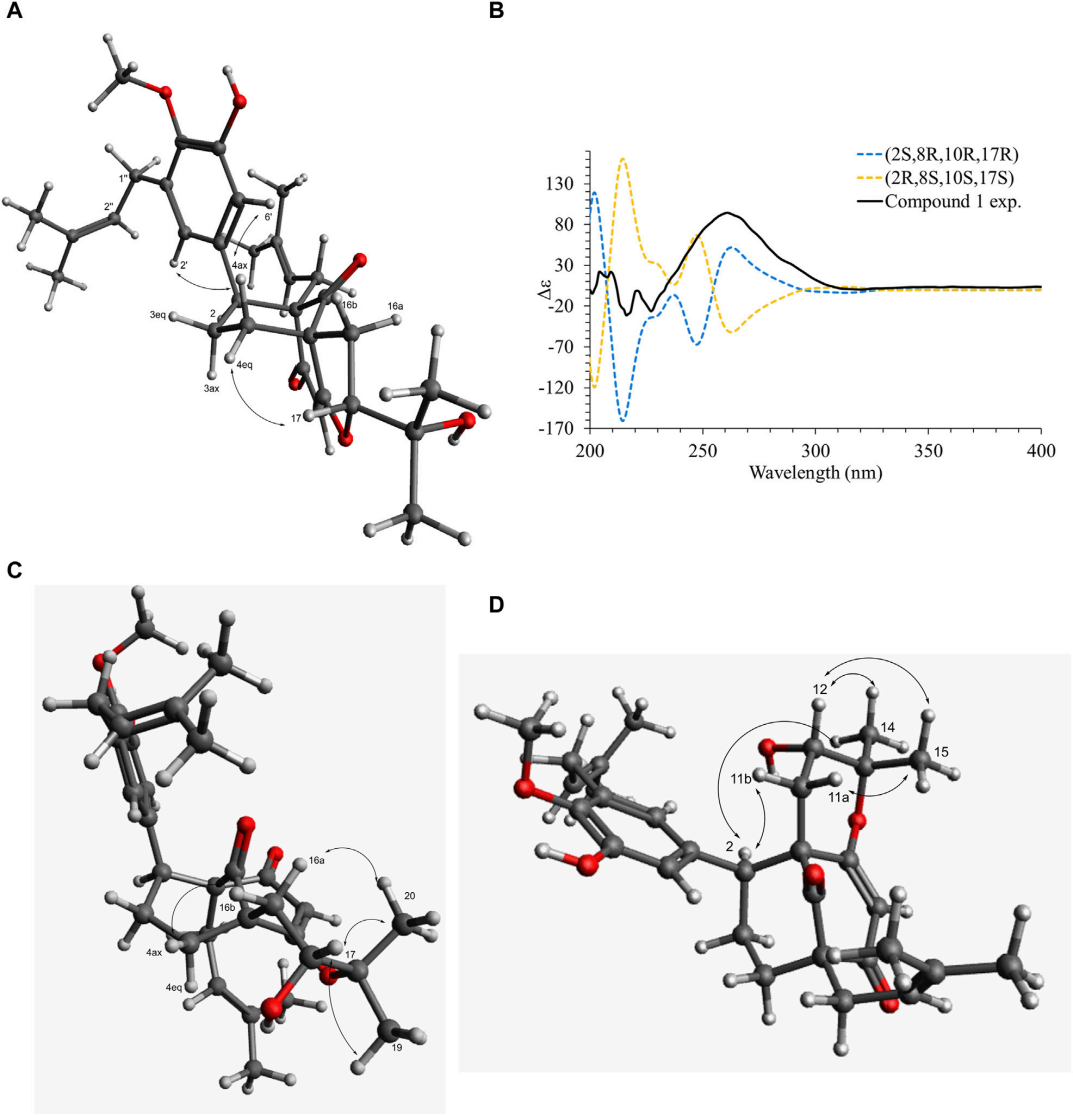
**(A)** Key dipolar correlations and ROESY spectrum for compound **1**. **(B)** Experimental and B3LYP/def2svp//B3LYP/6-31G(d,p) calculated ECD spectra in methanol for compound **1**. **(C)** Key dipolar correlations for compound **6**. **(D)** Key dipolar correlations for compound **9**.

Compound **1** was isolated as an amorphous pale yellow powder with an [M + H]^+^ of *m/z* 509.2894 corresponding to a molecular formula (MF) of C_31_H_41_O_6_ (error −0.65 ppm) (see Table 1). It corresponded to the peak at 4.4 min in Figure 3. The NMR data showed signals related to a 3-prenyl-4-methoxy-5-hydroxyphenyl group; with two meta aromatic protons coupled to each other (*J* = 2.3 Hz) at δ_H_ 6.38 (CH-2′) and δ_H_ 6.47 (CH-6′), a methoxy singlet at δ_H_ 3.75 (4′-OCH_3_), a methylene at δ_H_ 3.28 (2H, m, CH_2_-1″), a vinylic proton at δ_H_ 5.24 (1H, thept, *J* = 6.7, 1.3 Hz, CH-2″) and two methyl groups at δ_H_ 1.73 (3H, d, *J* = 1.3 Hz, CH_3_-4″) and δ_H_ 1.77 (3H, d, *J* = 1.3 Hz, CH_3_-5″). This part of the molecule was confirmed based on the HMBC correlations from CH-2′ to C-1'' (δ_C_ 29.1), C-4' (δ_C_ 146.0), and C-6' (δ_C_ 115.8), from H-6′ to C-2' (δ_C_ 121.6), C-4′ and C-5' (δ_C_ 151.0), from CH_3_-4″ and CH_3_-5″ to C-3'' (δ_C_ 133.6) and C-2'' (δ_C_ 123.9), from CH_2_-1″ to C-2′, C-3' (δ_C_ 136.3), C-4′, C-2″ and C-3″, and from 4′-OCH_3_ to C-4' (see Figure 5). A second prenyl group was identified based on the signals at δ_H_ 2.05 and δ_H_ 2.17 for the methylene CH_2_-11, δ_H_ 4.76 for the ethylenic proton CH-12, and δ_H_ 1.48 and δ_H_ 1.50 for the two methyl groups CH_3_-14 and CH_3_-15. The COSY correlation from the methylene CH_2_-16 (δ_H_ 1.91 and δ_H_ 2.82) to the oxymethine CH-17 (δ_H_ 4.72) and the HMBC correlations from the methyl groups CH_3_-19 and CH_3_-20 (δ_H_ 1.21 and δ_H_ 1.34) to C-18 (δ_C_ 71.1) and CH-17 (δ_C_ 92.8) allowed the identification of a 3-methylbutyl chain with positions two and three oxygenated. The aromatic moiety from the 3-prenyl-4-methoxy-5-hydroxyphenyl group was shown to be linked to a methine, which is connected to two consecutive methylenes. This was evidence thanks to the HMBC correlations from the methine CH-2 (δ_H_ 3.12) to C-1' (δ_C_ 137.9), C-2′, C-6′, the methylenes CH_2_-3 (δ_C_ 27.5) and CH_2_-4 (δ_C_ 33.1), as well as the one from CH-3_eq_ (δ_H_ 1.64) to C-1' (see Figure 5). A vinylic singlet at δ_H_ 5.80 (CH-6), two quaternary carbons (δ_C_ 61.7 (C-10) and 67.2 (C-8)), and three carbonyl carbons (δ_C_ 181.2 (C-5), 201.3 (C-7), and 207.7 (C-9)) fitted the number of carbons (31) with the molecular formula. The HMBC correlations from CH_2_-11 to CH-2, C-7, C-8, and C-9, from CH_2_-16 to CH_2_-4, C-5, C-9, and C-10, from CH-6 to C-5, C-8, and C-10, from CH-2 to C-7, and C-9, and from CH_2_-4 to C-5 allowed to position the 3-prenyl-4-methoxy-5-hydroxyphenyl group, the prenyl, and the 2,3-dihydroxy-3-methylbutyl group on a bicyclic[3.3.1] core as shown in Figure 4. The carbons C-8 and C-10 are bridged by a ketone (C-9) with a typical chemical shift (δ_C_ 207.7). These carbons together with CH-2, CH_2_-3, and CH_2_-4 integrate the first 6-member ring. The second ring shares the carbons C-8, C-9, and C-10 with a keto-enol system between carbons C-5, C-6, and C-7. According to the molecular formula, an additional ring must be established either between the tertiary alcohol in C-18 and C-5 to form a tetrahydropyran ring, or between the secondary alcohol in C-17 and C-5 to form a tetrahydrofuran ring. The lack of HMBC correlation between CH-17 and C-5 could not answer this question. Measurements in CDCl_3_ were done to try to find correlations between these protons by avoiding solvent exchanges, but they did not show any correlations either. However, the CH-17 and C-18 chemical shift values (δ_C_ 92.8 and δ_C_ 71.1, respectively) compared to values in the literature for oblongifolin R (which presents a tetrahydrofuran ring with CH at δ_C_ 94.8 and C at δ_C_ 71.1) and oblongifolin S (bearing a tetrahydropyran ring with CH at δ_C_ 69.6 and C at δ_C_ 86.9) (Zhang et al., 2014) led to the conclusion that a 5-member ring is present. The two-dimensional structure of compound **1** was established as shown in Figure 4. The relative configuration was established through the ROESY correlations observed in CD_3_OD and CDCl_3_. The spectra recorded in CDCl_3_ helped identify the pseudo equatorial and axial positions of CH_2_-3 and CH_2_-4 protons. These proton signals overlapped with other ones in CD_3_OD. The ROESY correlations from CH-2'/CH-6′ to CH-4_ax_ indicated that the 3-prenyl-4-methoxy-5-hydroxyphenyl group was in a pseudo axial position and the coupling constant value of CH-2 (*J* = 5.4 Hz in CD_3_OD, *J* = 6.1 Hz in CDCl_3_) confirmed that CH-2 was in a pseudo equatorial position (Figure 6A). The correlation between CH-17 and CH-4_eq_ indicated the relative configuration of C-17 (Figure 6A). Consequently, the relative configuration of **1** was proposed as (2*S*,8*R*,10*R*,17*R*) or (2*R*,8*S*,10*S*,17*S*). To establish its absolute configuration, the ECD spectrum was calculated based on the relative configuration proposed by NMR and compared to the experimental data (Figure 6B). Thus, compound **1** was assigned as (2*S*,8*R*,10*R*,17*R*)-2-(5-hydroxy, 4-methoxy-3-(3-methylbut-2-en-1-yl)phenyl)-12-(2-hydroxypropan-2-yl)-8-(3-methylbut-2-en-1-yl)-5-oxotricyclo[6.3.1.0^5,10^]dodec-5-ene-7,9-dione and named Hymenotamayonin G.

**TABLE 1:** ^1^H NMR (600 MHz) and^13^C NMR (151 MHz) data of compounds 1, 2, 4, 6, and eight in CD_3_OD. NA: signal not detected due to the keto-enol tautomerism in system C(5)-C(6)-C(7).

No.	Compound 1		Compound 2		Compound 4		Compound 6		Compound 8	
	δ_H_ (multi, *J* in Hz)	δ_C_	δ_H_ (multi, *J* in Hz)	δ_C_	δ_H_ (multi, *J* in Hz)	δ_C_	δ_H_ (multi, *J* in Hz)	δ_C_	δ_H_ (multi, *J* in Hz)	δ_C_
**2ax eq**	3.12 (d, 5.4)	55.2	3.07 (d, 6.4)	53.8	−3.40 (d, 6.4)	49.4	3.03 (d, 6.2)	55.0	3.12 (d, 6.4)	53.6
**3ax eq**	2.28 m) 1.64 (overlapped)	27.5	2.34 (tt, 14.3,5.8) 1.55 (overlapped)	28.1	2.36 (tt, 15.0, 14.1, 6.4, 5.0) 1.67 (overlapped)	27.8	2.26 (tt, 14.2, 5.6) 1.55 (brd, 10.8)	27.3	2.31 (tt, 13.8, 6.4) 1.56 (overlapped)	27.8
**4ax eq**	2.29 m) 2.16 (overlapped)	33.1	2.17 (td, 13.8,4.9) 1.79 (dd, 13.8,5.2)	34.7	2.08 (td, 14.1,4.8) 1.78 (overlapped)	34.9	2.09 (td, 14.5, 4.6) 2.63 (dd, 14.5, 4.9)	36.2	2.17 (td, 13.8, 4.7) 1.79 (overlapped)	34.5
**5**	-	181.2	-	NO	-	200.7	-	176.5	-	NO
**6**	5.80 s)	104.3	NO	NO	5.81 s)	104.7	5.88 s)	114.7	NO	NO
**7**	-	201.3	-	NO	-	182.1	-	200.7	-	NO
**8**	-	67.2	-	64.7	-	64.7	-	67.5	-	64.4
**9**	-	207.7	-	211.5	-	208.0	-	209.7	-	210.4
**10**	-	61.7	-	61.6	-	64.4	-	51.6	-	61.7
**11**	2.17 (dd, 14.3, 7.9) 2.05 (dd, 14.3, 6.0)	30.4	2.09 m) 2.09 m)	30.3	2.53 m) 1.74 (overlapped)	28.7	2.15 (dd, 14.7,7.8) 2.07 (overlapped)	30.5	2.15 (overlapped) 2.08 (dd, 14.2,5.2)	30.1
**12**	4.76 (thept, 7.9, 6.0, 1.2)	121.1	4.87 (overlapped)	122.0	4.62 (dd, 11.1,5.4)	92.2	4.81 (thept, 7.8, 1.4)	121.2	4.87 (overlapped)	121.4
**13**	-	134.1	-	133.2	-	71.1	-	134.0		133.8
**14**	1.48 (d, 1.2)	18.0	1.47 (d, 1.5)	18.0	1.28 s)	26.3	1.47 (d, 1.4)	18.0	1.46 (d, 1.5)	18.0
**15**	1.50 (d, 1.2)	26.1	1.55 (d, 1.5)	26.1	1.10 s)	25.2	1.53 (d, 1.4)	26.1	1.55 (d, 1.5)	26.1
**16**	2.82 (t, 12.9, 10.9) 1.91 (dd, 12.9, 5.5)	30.8	2.57 (dd, 14.5, 6.9) 2.47 (dd, 14.5, 6.9)	31.1	2.53 m) 2.43 (dd, 14.5,6.9)	31.1	2.94 (dd, 14.9, 3.9) 1.74 (dd, 14.9, 5.3)	31.5	2.58 (dd, 14.5, 7.1) 2.49 (dd, 14.5, 7.1)	30.9
**17**	4.72 (dd, 10.9, 5.5)	92.8	5.19 (thept, 6.9, 1.5)	121.9	5.07 (brt, 7.3)	121.1	3.79 (t, 5.3, 3.9)	69.9	5.19 (thept, 7.1,1.5)	121.4
**18**	-	71.1	-	133.9	-	134.5	-	84.8	-	134.4
**19**	1.34 s)	26.5	1.70 (d, 1.5)	18.2	1.68 s)	18.2	1.44 s)	24.0	1.68 (d, 1.4)	18.2
**20**	1.21 s)	25.4	1.68 (d, 1.5)	26.1	1.63 s)	26.1	1.26 s)	26.9	1.70 (d, 1.4)	26.2
**1′**	-	137.9	-	133.4	-	138.1	-	138.1	-	138.2
**2′**	6.38 (d, 2.3)	121.6	6.28 (d, 2.2)	121.4	6.53 (d, 2.2)	115.9	6.31 (d, 2.3)	121.4	6.34 (d, 2.3)	121.6
**3′**	-	136.3	-	129.4	-	136.7	-	136.2	-	136.2
**4′**	-	146.0	-	143.0	-	146.1	-	145.9	-	145.9
**5′**	-	151.0	-	145.8	-	151.4	-	151.0	-	150.9
**6′**	6.47 (d, 2.3)	115.8	6.38 (d, 2.2)	114.8	6.43 (d, 2.2)	121.3	6.42 (d, 2.3)	116.1	6.44 (d, 2.3)	116.2
**1**″	3.28 m) 3.28 m)	29.1	3.25 m) 3.25 m)	29.2	3.28 (d, 7.4)	29.2	3.28 (d, 7.5)	29.0	3.27 m) 3.27 m)	29.3
**2**″	5.24 (thept, 6.7, 1.3)	123.9	5.30 (thept, 7.4, 1.4)	123.9	5.23 (brt, 7.4)	123.8	5.25 (thept, 7.5, 1.3)	123.8	5.25 (thept, 7.5, 1.4)	124.0
**3**″	-	133.6	-	133.2	-	133.6	-	133.8	-	133.4
**4**″	1.73 (d, 1.3)	17.9	1.72 (d, 1.4)	17.9	1.73 s)	18.0	1.74 (d, 1.3)	17.9	1.74 (d, 1.4)	18.0
**5**″	1.77 (d, 1.3)	26.0	1.76 (d, 1.4)	26.0	1.76 s)	26.0	1.79 (d, 1.3)	26.1	1.76 (d, 1.4)	26.0
**4′OMe**	3.75 s)	60.8			3.74 s)	60.8	3.75 s)	60.8	3.74 s)	60.8

Compound **2** was isolated as an amorphous pale yellow powder with an [M + H]^+^ of *m/z* 479.2793 corresponding to MF of C_30_H_39_O_5_ (error 0.21 ppm) (see Table 1). It corresponded to the peak at 4.5 min in Figure 3. The ^1^H NMR data compared to that of **1** showed that: i) the vinylic singlet at δ_H_ 5.80 (CH-6) was missing, ii) the methoxy (4′-OCH_3_) signal was absent, and iii) signals from the 2,3-dihydroxy-3-methylbutyl chain were replaced by those belonging to a third prenyl group (a methylene CH_2_-16 at δ_H_ 2.47/2.57, a vinylic proton CH-17 at δ_H_ 5.19, and two methyl CH_3_-20/CH_3_-19 δ_H_ 1.68/1.70). This prenyl group was positioned in C-10 thanks to the HMBC correlations from CH_2_-16 to the methylene CH_2_-4 (δ_C_ 34.7), the quaternary carbon C-10 (δ_C_ 61.6) and the ketone C-9 (δ_C_ 211.9). To fit with the molecular formula, an enolized 1,3-diketone was placed in C-5, C-6, and C-7. Due to the rapid tautomeric equilibrium and H/D exchange with the solvent, the ^1^H and ^13^C signals of C-5, CH-6, and C-7 were not observed (Traven et al., 1997; Rajaonarivelo et al., 2016) (See Supplementary Figure S5). The relative configuration of **2** was the same as described for **1**, and the absolute configuration was confirmed with the comparison between the calculated and experimental ECD spectra (See Supplementary Figure S6). Thus compound **2** was identified as (2*S*,8*R*,10*R*)-(4,5-dihydroxy-3-(3-methylbut-2-en-1-yl)phenyl)-5-hydroxy-8,10-bis(3-methylbut-2-en-1-yl)bicyclo[3.3.1]non-6-ene-7,9-dione and named Hymenotamayonin A.

The molecular weight of compound **4,** *m/z* 509.2894 [M + H]^+^ (calculated for MF C_31_H_41_O_6_, error −0.65 ppm) was the same as compound **1** and the NMR data were also (see Table 1) very close with the same functional groups: a 3-prenyl-4-methoxy-5-hydroxyphenyl, a prenyl group, a 2,3-dihydroxy-3-methylbutyl chain, all attached to a bicyclic[3.3.1] core. The difference between **1** and **4** was the positions of the prenyl group and the 2,3-dihydroxy-3-methylbutyl chain in the bicyclic core. In **4**, the HMBC correlations from the methylene CH_2_-16 of the prenyl at δ_H_ 2.43/2.53 to CH_2_-4 (δ_C_ 27.8), C-10 (δ_C_ 64.4), C-5 (δ_C_ 200.7), and C-9 (δ_C_ 208.0), and from the methylene CH_2_-11 of the 2,3-dihydroxy-3-methylbutyl chain at δ_H_ 1.74/2.53 to CH-2 (δ_C_ 49.4), C-8 (δ_C_ 64.7), and C-7 (δ_C_ 182.1) indicated that the prenyl was in C-10 and the 2,3-dihydroxy-3-methylbutyl chain was in C-8. As in **1**, the chemical shift of CH-12 (δ_C_ 92.2) and C-13 (δ_C_ 71.1) showed that a tetrahydrofuran ring is present between CH-12 and C-7. The coupling constant of CH-2 (d, *J* = 6.4 Hz) and the ROESY from the aromatic protons CH-2′ and CH-6′ to CH-4ax at δ_H_ 2.08 (td, *J* = 14.1, 4.8 Hz) indicated that as in **1**, that the aromatic group is in a pseudo axial configuration. The ROESY correlation from CH-2 to the methine CH-12 of the tetrahydrofuran group showed that the configuration of C-12 and C-2 is *S*. Comparison of the experimental and calculated ECD spectra corroborated this observation (See Supplementary Figure S6). Compound **4** was identified as (2*S*,8*R*,10*R*,12*S*)-2-(5-hydroxy, 4-methoxy-3-(3-methylbut-2-en-1-yl)phenyl)-17-(2-hydroxypropan-2-yl)-10-(3-methylbut-2-en-1-yl-7-oxotricyclo[6.3.1.0^7,8^]dodec-6-ene-5,9-dione and named Hymenotamayonin H. It corresponded to one of the major peak at 4.6 min in both, MS and CAD traces (Figure 3).

Similar to **1**, compound **6** exhibited an MF of C_31_H_40_O_6_ (*m/z* 509.2894 [M + H]^+^, error −0.65 ppm) (see Table 1). The NMR data of **6** were very close to those of **1** and indicated that the connectivity and configuration of the bicyclic[3.3.1] core, the pseudo axial position of the 3-prenyl-4-methoxy-5-hydroxyphenyl group in C-2, the linkage of the prenyl group in C-8 were the same as those described for **1**. The main differences concerned the 2,3-dihydroxy-3-methylbutyl chain for which the chemical shift values of the oxymethine C-17 were observed at δ_C_ 69.9 (δ_C_ 92.8 for **1**) and the quaternary oxygenated carbon C-18 at δ_C_ 84.8 (δ_C_ 71.1 for **1**). This agreed with the presence of a tetrahydropyran ring (Figure 4). The configuration at C-17 was determined thanks to the ROESY correlation from CH-16eq at δ_H_ 1.74 (dd, *J* = 14.9, 5.3 Hz) to CH-4ax at δ_H_ 2.09 (td, *J* = 14.5, 4.6 Hz), from CH-16ax at δ_H_ 2.94 (dd, *J* = 14.9, 3.9 Hz) to CH_3_-20 at δ_H_ 1.26 (Figure 6C). The coupling constant value of CH-17 (t, *J* = 5.3, 3.9 Hz) and its ROESY correlations with CH_3_-19 and CH_3_-20 indicated its equatorial position. The configuration proposed was confirmed by comparison of the experimental and calculated ECD spectra (See Supplementary Figure S6). Compound **6** was then identified as (2*S*,8*R*,10*R*,17*R*)-17-hydroxy-2-(5-hydroxy, 4-methoxy-3-(3-methylbut-2-en-1-yl)phenyl)-18,18-dimethyl-8-(3-methylbut-2-en-1-yl)-5-oxotricyclo[7.3.1.0^5,10^]dodec-5-ene-7,9-dione and named Hymenotamayonin I. It corresponded to one of the peaks at 4.8 min in Figure 3. (Table 2)

**TABLE 2:** ^1^H NMR (600 MHz) and^13^C NMR (151 MHz) data of compounds 9–13 in CD_3_OD. NA: signal not detected due to the keto-enol tautomerism in system C(5)-C(6)-C(7).

No.	Compound 9		Compound 10		Compound 11		Compound 12		Compound 13	
	δ_H_ (multi, *J* in Hz)	δ_C_	δ_H_ (multi, *J* in Hz)	δ_C_	δ_H_ (multi, *J* in Hz)	δ_C_	δ_H_ (multi, *J* in Hz)	δ_C_	δ_H_ (multi, *J* in Hz)	δ_C_
**2ax eq**	3.63 (d, 6.2)	51.9	2.64 (dd, 13.2,4.0) -	56.2	2.68 (dd, 13.1, 4.0) -	55.7	−3.04 (d, 6.2)	54.7	−3.14 (d, 3.8)	55.3
**3ax eq**	2.36 (tt, 14.2, 6.2, 4.7) 1.53 (dt, 14.2, 4.7, 2.1)	27.5	2.21 m) 1.71 (overlapped)	28.1	2.25 (td, 13.1, 4.8) 1.71 (overlapped)	28.2	2.23 m) 1.63 m)	27.6	2.32 (overlapped) 1.66 m)	27.6
**4ax eq**	2.11 (td, 14.2,4.7) 1.64 (ddd, 14.2, 4.7, 2.1)	33.5	1.64 (overlapped) 1.97 (ddd, 13.1, 4.9, 1.8)	39.2	1.63 (overlapped) 1.98 (ddd, 12.9, 4.8, 1.8)	39.0	1.96 (td, 13.3,4.5) 2.28 (dd, 13.3, 4.5)	33.2	2.30 (overlapped) 2.23 (brd, 9.4)	32.6
**5**	-	200.2	-	NO	-	NO	-	176.8	-	NO
**6**	5.89 s)	115.7	NO	NO	NO	NO	5.82 s)	113.8	5.80 s)	104.2
**7**	-	177.4	-	NO	-	NO	-	200.4	-	201.3
**8**	-	55.3					-	67.8	-	67.3
**9**	-	209.7	-	211.0	-	211.1	-	210.3	-	207.8
**10**	-	65.0					-	51.0	-	61.7
**11**	2.61 (dd, 14.7,3.9) 1.59 (dd, 14.7,7.8)	28.7	2.30 (dd, 15.0,6.7) 2.21 m)	29.8	2.32 (dd, 14.9, 7.2) 2.18 (dd, 14.9, 7.2)	29.7	2.16 (dd, 14.7, 7.8) 2.06 (dd, 14.7, 6.1)	30.3	2.17 (dd, 14.5,7.7) 2.07 (dd, 14.5,6.0)	30.5
**12**	3.49 (dd, 7.8,3.9)	70.5	4.87 (overlapped)	121.9	4.89 (overlapped)	122.0	4.83 (thept, 7.6, 1.3)	121.3	4.78 (ddhept, 7.7, 6.0, 1.4)	121.0
**13**	-	85.3	-	133.3	-	133.1				134.2
**14**	1.40 s)	22.9	1.51 (d, 1.5)	18.2	1.51 (d, 1.6)	18.2	1.47 (d, 1.3)	17.9	1.49 (d, 1.4)	17.9
**15**	1.26 s)	27.1	1.59 (d, 1.5)	26.1	1.60 (d, 1.6)	26.1	1.54 (d, 1.3)	26.0	1.53 (d, 1.4)	26.0
**16**	2.54 (dd, 14.5,7.7) 2.44 (dd, 14.5,6.8)	30.8	2.48 m) 2.48 m)	30.9	2.50 (dd, 14.3, 7.0) 2.46 (dd, 14.3, 7.0)	31.0	2.56 (td, 14.4, 4.2) 1.71 (dt, 14.4, 4.2)	24.2	2.63 (dd, 13.0, 11.2) 2.12 (dd, 13.0, 5.4)	35.4
**17**	5.17 (thept, 7.7, 6.8, 1.3)	121.2	4.97 (thept, 6.9, 1.5)	121.4	4.98 (thept, 7.0, 1.6)	121.6	2.04 (td, 14.3, 4.1) 1.86 (dt, 14.3, 4.1)	31.2	5.32 (dd, 11.2 5.4)	89.2
**18**	-	134.7	-	134.0	-	134.1	-	82.3	-	143.1
**19**	1.69 (d, 1.3)	18.2	1.69 (d, 1.5)	18.2	1.69 (d, 1.3)	18.2	1.46 s)	30.1	1.78 (t, 1.2)	17.3
**20**	1.68 (d, 1.3)	26.2	1.63 (d, 1.5)	26.1	1.63 (d, 1.3)	26.1	1.29 s)	26.2	5.17 (q, 1.2) 5.05 (q, 1.2)	114.4
**1′**	-	138.2	-	131.3	-	136.6	-	138.1	-	137.9
**2′**	6.31 (d, 2.3)	121.5	6.32 (d, 2.2)	122.6	6.38 (d, 2.2)	122.9	6.31 (d, 2.3)	121.4	6.38 (d, 2.3)	121.6
**3′**	-	136.4	-	128.7	-	135.4	-	136.3	-	136.4
**4′**	-	146.0	-	143.4	-	146.2	-	145.9	-	146.0
**5′**	-	151.3	-	145.3	-	150.4	-	151.0	-	151.1
**6′**	6.46 (d, 2.3)	116.9	6.40 (d, 2.2)	114.4	6.47 (d, 2.2)	116.0	6.41 (d, 2.3)	115.9	6.47 (d, 2.3)	115.8
**1**″	3.27 m) 3.27 m)	29.4	3.28 (dd, 15.6,7.4) 3.23 (dd, 15.6,7.4)	29.2	3.27 m) 3.27 m)	29.4	3.28 m) 3.28 m)	29.0	3.27 m) 3.27 m)	29.1
**2**″	5.24 (thept, 8.1, 1.3)	124.0	5.29 (thept, 7.4, 1.4)	124.2	5.24 (thept, 7.2, 1.3)	124.5	5.24 (thept, 7.5, 1.3)	123.8	5.25 (thept, 7.5, 1.3)	123.9
**3**″	-	133.6	-	132.7	-	132.9	-	134.0	-	133.5
**4**″	1.74 (d, 1.3)	18.0	1.71 (d, 1.4)	17.9	1.73 (d, 1.3)	18.0	1.73 (d, 1.3)	18.0	1.73 (d, 1.3)	18.0
**5**″	1.77 (d, 1.3)	26.0	1.73 (d, 1.4)	26.0	1.73 (d, 1.3)	25.9	1.78 (d, 1.3)	26.1	1.77 (d, 1.3)	26.1
**4′OMe**	3.73 s)	60.7			3.73 s)	60.7	3.74 s)	60.8	3.75 s)	60.8

The HRMS and NMR data (Table 1) showed that compound **8 (**Hymenotamayonin C) was the *O*-methyl derivative of **2**: (2*S*,8*R*,10*R*)-2-(5-hydroxy-4-methoxy-3-(3-methylbut-2-en-1-yl)phenyl)-5-hydroxy-8,10-bis(3-methylbut-2-en-1-yl)bicyclo[3.3.1]non-6-ene-7,9-dione. Thus, the group in C-2 was the same as **1**, **4,** and **6**: a 3-prenyl-4-methoxy-5-hydroxyphenyl group. It corresponded to one of the peaks at 4.9 min in Figure 3.

The NMR data of **9** (Table 2) displayed very close similarities to those of **4**, for the same MF as indicated by the HRMS ion at *m/z* 509.2893 [M + H]^+^. As for the previous comparison between **6** and **1**, the difference between **9** and **4** was the presence of a tetrahydropyran ring in **9** whereas **4** had a tetrahydrofuran ring in the same place. This was indicated by the chemical shift’s values of CH-12 (δ_C_ 70.5) and C-13 (δ_C_ 85.3), in **9** compared to those of **4** (δ_C_ 92.2 and δ_C_ 71.1, respectively). The ROESY from CH-11eq at δ_H_ 1.59 (dd, *J* = 14.7, 7.8 Hz) to CH-2 at δ_H_ 3.63 (d, *J* = 6.2 Hz), from CH-11ax at δ_H_ 2.61 (dd, *J* = 14.7, 3.9 Hz) to CH_3_-15 at δ_H_ 1.26 indicated the *S* configuration at C-12 (See Figure 6D). Thus, compound **9** was identified as (2*S*,8*R*,10*R*,12*S*)-12-hydroxy-2-(5-hydroxy, 4-methoxy-3-(3-methylbut-2-en-1-yl)phenyl)-13,13-dimethyl-10-(3-methylbut-2-en-1-yl)-7-oxotricyclo[7.3.1.0^7,8^]dodec-5-ene-5,9-dione and named Hymenotamayonin J. Its absolute configuration was confirmed based on the comparison of the experimental and calculated ECD spectra (See Supplementary Figure S6) (see Table 2).

The NMR data of **10** were closely related to those of **2** and the HRMS data confirmed that both molecules had the same MF C_30_H_39_O_5_ (*m/z* 479.2791 [M + H]^+^, error −0.19 ppm). The main differences were the chemical shift value of CH-2 and its coupling constants. Whereas the chemical shift values of CH-2 were observed above δ_H_ 3.00 in the previous compounds it appeared shielded in **10** at δ_H_ 2.64. In the previous compounds, CH-2 was observed as a doublet with a coupling constant between 5.4 Hz and 6.4 Hz. In **10**, CH-2 resonated as a doublet of doublet (*J* = 13.2, 4.0 Hz) indicating that it was, in a pseudo-axial configuration. This was confirmed by the ROESY correlation from CH-2 to CH-4ax at δ_H_ 1.64 (overlapped). Thus, compound **10** was identified as (2*R*,8*R*,10*R*)-2-(4,5-dihydroxy-3-(3-methylbut-2-en-1-yl)phenyl)-5-hydroxy-8,10-bis(3-methylbut-2-en-1-yl)bicyclo[3.3.1]non-6-ene-7,9-dione and named Hymenotamayonin B. It corresponded to one of the peaks at 4.95 min in Figure 3 (see Table 2).

The NMR and HRMS data of **11** indicated that it was the 4′-*O*-methyl derivative of **10**, thus (2*R*,8*R*,10*R*)-2-(5-hydroxy-4-methoxy-3-(3-methylbut-2-en-1-yl)phenyl)-5-hydroxy-8,10-bis(3-methylbut-2-en-1-yl)bicyclo[3.3.1]non-6-ene-7,9-dione (Hymenotamayonin D). It corresponded to one of the peaks at 5.25 min in Figure 3. The HRMS of **12** gave an [M + H]^+^ at *m/z* 493.2747 calculated for an MF C_31_H_41_O_5_ (error −0.16 ppm) (see Table 2). The NMR data showed as in **6**, that the bicyclic[3.3.1] core presented a 3-prenyl-4-methoxy-5-hydroxyphenyl substitution in a pseudo axial configuration in C-2 and a prenyl group in C-8. The oxymethine CH-17 observed in the tetrahydropyran ring of **6** was replaced by a methylene (δ_H_ 2.04 (td, *J* = 14.3, 4.1 Hz, CH-17ax), δ_H_ 1.86 (dt, *J* = 14.3, 4.1 Hz, CH-17eq) and δ_C_ 31.2). The HMBC correlations from CH-16ax (δ_H_ 2.56, td, *J* = 14.4, 4.2 Hz) to CH_2_-4 (δ_C_ 33.2) and C-10 (δ_C_ 51.0), from CH-17eq to C-10, from CH-16eq (δ_H_ 1.71, dt, *J* = 14.4, 4.2 Hz), CH_3_-19 (δ_H_ 1.46) and CH_3_-20 (δ_H_ 1.29) to quaternary oxygenated carbon C-18 (δ_C_ 82.3) confirmed that **12** was the 17-deoxy derivative of **6**: (2*S*,8*R*,10*R*)-2-(5-hydroxy, 4-methoxy-3-(3-methylbut-2-en-1-yl)phenyl)-18,18-dimethyl-8-(3-methylbut-2-en-1-yl)-5-oxotricyclo[7.3.1.0^5,10^]dodec-5-ene-7,9-dione (Hymenotamayonin F). It corresponded to one of the peaks at 5.45 min in Figure 3.

The MF of **13** was established as C_31_H_38_O_5_ by HRMS (*m/z* 491.2792 [M + H]^+^, error 0.08 ppm). As compounds **1**, **6**, **8**, **11,** and **12**, the NMR data of **13** (Table 2) showed signals corresponding to a bicyclic[3.3.1] core with, in C-2, a 3-prenyl-4-methoxy-5-hydroxyphenyl in a pseudo axial configuration and, in C-8, a prenyl group. Similar to **1**, a tetrahydrofuran ring was observed between C-10 and C-5, as indicated by the signals from the methylene CH_2_-16 (δ_H_ 2.63/δ_H_ 2.12) and the oxymethine CH-17 (δ_H_ 5.32). The HMBC correlations from the methylidene observed at δ_H_ 5.17 (q, *J* = 1.2 Hz, CH-20a) and δ_H_ 5.05 (p, *J* = 1.2 Hz, CH-20b) to the oxymethine CH-17 (δ_C_ 89.2), the vinylic carbon C-18 (δ_C_ 143.1) and the methyl CH_3_-19 (δ_C_ 17.3) enabled the identification of the structure shown in Figure 4. The configuration in C-17 was determined as *R* thanks to the ROESY correlations from CH-17 to CH-4eq (δ_H_ 2.23, brd, J = 9.4 Hz, the relative configuration of the other carbons were established as (2*S*,8*R*,10*R*). Thus, compound **13** was identified as (2*S*,8*R*,10*R*,17*R*)-2-(5-hydroxy, 4-methoxy-3-(3-methylbut-2-en-1-yl)phenyl)-8-(3-methylbut-2-en-1-yl)-17-(prop-1-en-2-yl-5-oxotricyclo[6.3.1.0^5,10^]dodec-5-ene-7,9-dione and named as Hymenotamayonin E. It corresponded to one of the peaks at 5.5 min in Figure 3.

Compounds **1**,**2**,**4**,**6**, and **8**–**13** make part of a very restricted group of NPs that contain an uncommon bicyclo[3.3.1]non-3-ene-2,9-dione core (Lin et al., 2018). To date, only three NPs with this core have been reported. The first compound ever reported in this class was acutifolin A (Q15410235), isolated from a Moraceae (Q156579) Brazilian medicinal plant *B. acutifolium* (Q15471077) (Takashima and Ohsaki, 2001). Later, tazettone A and tazettone B were isolated from *Narcissus tazetta* var. *chinensis* (Q25115128) (Fu et al., 2013), an Amaryllidaceae (Q155848). According to the Angiosperm (Q14832431) phylogeny grouping, *N. tazzeta* belongs to the Asparagales order (Q26229), while *Brosimum acutifolium* and *H. punctata* are comprised in the Fabids clade (Q2683213) but in different orders, Rosales (Q21895) and Malpighiales (Q21887), respectively. There are therefore no closer taxonomic relationships between these species that could explain common biosynthetic pathways (The Angiosperm Phylogeny Group, 2009).

**FIGURE 7:**
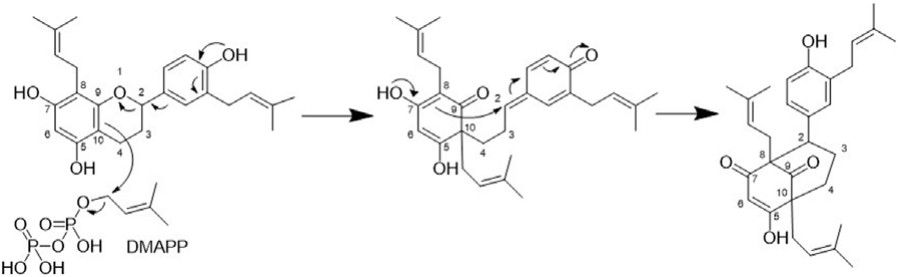
Proposed biosynthesis for the bicyclo[3.3.1]non-3-ene-2,9-dione core of compounds **1**,**2**,**4**,**6**, and **8**-**13**.

From a biosynthetic point of view, the new compounds isolated from *H. punctata* may have been formed by rearrangement of an 8-prenylflavane as suggested for acutifolin A, tazettone A, and tazettone B (Figure 7). A similar rearrangement occurs also when catechin is subjected to basic conditions, forming catechinic acid (Sears et al., 1974; Ibrahim et al., 2007; Khiari et al., 2017). The extraction process was under neutral conditions, which is an indication of the authenticity of the compounds. The proposed biosynthesis pathway involves the presence of an electrophilic species that introduces the hydroxy group at position C-10 in acutifolin A, and tazettone A and B. We hypothesize that, in our case, this electrophilic species is a dimethylallyl diphosphate (DMAPP) unit, resulting in the prenylation of the position C-10 (Yazaki et al., 2009; Zhou et al., 2021). This assertion is plausible since we were able to isolate prenylated flavonoids (**3**, **5** and **7**), which is an indication of their abundance in this plant.

### Evaluation of the Wnt-regulatory activity of isolated compounds

The Wnt-regulatory activity of all isolated compounds from *H. punctata* was evaluated using two different cancer cell lines representing TNB cancer, known for its reliance on Wnt signaling: BT-20 and HCC1395 cells. Additionally, Human Embryonic Kidney HEK293 cells were used to represent non-malignant cells. The results for the active compounds are shown in Table 3 (and Supplementary Figure S7).

**TABLE 3:** Wnt-inhibition assay results IC_50_ (*µ*M) for the isolated compounds **one to four** and **7**.

Compound	HCC1395	BT-20	HEK293
**1**	15.0 ± 0.3	51 ± 25	35 ± 1
**2**	17 ± 1	28 ± 10	26 ± 4
**3**	13 ± 1	27 ± 8	40.0 ± 0.2
**4**	14.0 ± 0.1	26 ± 1	31 ± 1
**7**	17 ± 5	16 ± 11	12 ± 2

The results demonstrated that the prenylated flavone **7**, exhibited the highest potency against the HEK293 cell line, with an IC_50_ value of 12 *µ*M. Other compounds exhibited significantly higher selectivity against HCC1395 cancer cells. For example, the other prenylated flavone **3** demonstrated the highest selectivity, followed by one of the newly discovered bicyclic compounds **4,** with IC_50_ values of 13 *µ*M and 14 *μ*M, respectively. Notably, their potency against both BT-20 and HEK293 cells was nearly two-fold lower. In the case of the BT-20 cell line, **seven** displayed the highest activity, followed by **4**, with IC_50_ values of 16 *µ*M and 26 *μ*M, respectively. It is worth noting that, in general, the prenylated flavones (**3** and **7**) had lower IC_50_ values compared to the novel bicyclic compounds (**1**, **2** and **4**) in at least two out of the three different cell models. Additionally, for all compounds, the specificity for Wnt inhibition was controlled by the absence of effects of the compounds on co-transfected constitutively expressed Renilla luciferase, serving as a reporter of cytotoxic or other negative effects of the compounds on the cell wellbeing (Shaw et al., 2019a).

Over the past 2 decades, numerous studies have revealed that flavonoids and structurally related compounds possess inhibitory effects on human diseases by targeting various cellular signaling components (Amado et al., 2011; 2014). Flavonoids have been recognized as inhibitors of the Wnt pathway, with many of them shown to inhibit it by downregulating the levels of *β-*catenin (Fuentes et al., 2015). For instance, Apigenin (Q424567), the first flavonoid to be reported as a Wnt inhibitor, has been found to decrease *β-*catenin levels and promote cell cycle arrest in breast and colorectal cancer (Song et al., 2000; Landesman-Bollag et al., 2001; Amado et al., 2011). However, to date, there have been no reported findings of whether and how prenylation changes the potency of flavones towards the Wnt/*β*-catenin pathway. A few reports show the direct activity of prenylated isoflavones, such as 8-prenylgenistein by promoting osteogenesis (Zhang et al., 2018; Qiu et al., 2020). Our results clearly demonstrate that prenylated flavones, and similar analogs like the new bicyclic compounds, selectively disrupt the Wnt/*β*-catenin pathway, however with a potency only modestly improved from that reported for apigenin, from *c. a*. 30 *μ*M down to 10–20 *µ*M. This is also paralleled by other studies showing that structurally similar prenylated chalcones such as derricin and derricidin isolated from *Lonchocarpus sericeus* (Q15471182) reduce cell viability and induce cell cycle arrest in colorectal cancer HCT116 cells (Q28334584) through negative modulation of the Wnt/*β*-catenin pathway (Stevens, 2020).

## Experimental section

### General experimental procedures

The plant material was extracted in a Thermo Scientific Dionex ASE 350 Accelerated Solvent Extractor (Thermo Scientific™, Bremen, Germany). HPLC analyses were performed on an HP 1260 Infinity Agilent High-Performance Liquid Chromatography System equipped with a photodiode array detector (HPLC-PDA) (Agilent Technologies, Santa Clara, CA, United States) connected to an Evaporative Light Scattering Detector (ELSD, SEDERE, Orleans, France). The HPLC-based biactivity profiling was performed on an HP 1260 Agilent Infinity II High-Performance liquid chromatography equipped with a photodiode array detector (HPLC-PDA) and a sample collector (Agilent Technologies, Santa Clara, CA, United States). Flash chromatography was performed on a Sepacore instrument (Buchi, Flawil, Switzerland) composed of a pump module C-605, fraction collector model C-620, and UV spectrophotometer model C-640. The semi-preparative HPLC was performed using a Shimadzu system consisting of LC-20A module pumps, an SPD-20A UV/Vis detector, a 7725I Rheodyne^®^ valve, and an FRC-10A fraction collector (Shimadzu, Kyoto, Japan). The system was controlled using the LabSolutions software from Shimadzu. NMR spectroscopic data were acquired on a Bruker Avance Neo 600 MHz spectrometer equipped with a QCI 5 mm Cryoprobe and a sampleJet automated extract changer (Bruker BioSpin, Rheinstetten, Germany). Chemical shifts are reported in parts per million (ppm, δ), and coupling constants are reported in Hertz (Hz, *J*). The residual CD_3_OD/CDCl_3_ signals (δ_H_ 3.31/7.26, δ_C_ 49.8/77.16) were used as internal standards for ^1^H and ^13^C, respectively. Comprehensive assignments were based on 2D-NMR spectroscopy techniques such as COSY, edited-HSQC, HMBC, and ROESY. Electronic Circular Dichroism (ECD) measurements were measured using a JASCO J-815 spectrometer (Loveland, CO, United States) in methanol, utilizing a 1 cm cell. The scan speed was set at 200 nm/min in continuous mode, scanning from 400 nm to 165 nm. Optical rotations were determined in methanol using a JASCO P-1030 polarimeter (Loveland, CO, United States) with a 1 mL, 10 cm tube.

### Extraction of plant material

PFL supplied the dried, grounded leaves of *H. punctata* (Phyllanthaceae) (identifier V114372GP-01). This plant was part of the PFL collection registered with the European Commission on 1 April 2020, under accession number 03-FR-2020. This official registration acknowledges the collection’s compliance with legal standards for access and management. It signifies that the PFL collection adheres to the European Access and Benefit Sharing Regulation criteria, which enforces the Nagoya Protocol’s directives at the European level. These directives pertain to accessing genetic resources and justly sharing the benefits derived from their use (Nagoya Protocol, 2011).

A mass of 55 g was extracted in a 100 mL pressure-resistant stainless steel extraction cell using an ASE system. At the bottom and the top of the cell, a cellulose filter (Dionex™ 100, Thermo Scientific™, Bremen, Germany) was added to prevent solid particles from reaching the internal system. The cell was extracted with 60% rinse volume under pressure at 40°C, three cycles, and a static time of 5 min per cycle. The sample was extracted successively with HPLC quality hexane (Fisher Chemicals, Reinach, Switzerland), ethyl acetate (Fisher Chemicals, Reinach, Switzerland), and methanol (Fisher Chemicals, Reinach, Switzerland). The resulting extracts were dried at 35°C under vacuum in a rotary evaporator to yield 0.32 g of hexanic extract (HPH), 1.13 g of ethyl acetate extract (HPE), and 2.60 g of methanolic extract (HPM).

### HPLC-based fractioning of HPE extract for bioactivity profiling

A mass of 10 mg of HPE was dissolved in 200 *μ*L of DMSO (molecular biology grade, Sigma, St Louis, United States) and then separated on an X-Bridge C_18_ column (250 × 10 mm i. d., 5 *μ*m) equipped with a Waters C_18_ precolumn cartridge holder (5 × 10 mm i. d., 5 *μ*m). The flow rate was set to 3.700 mL/min, and a binary solvent system consisting of 0.1% aqueous formic acid [A] and 0.1% formic acid in ACN [B] (Fisher Chemicals, Reinach, Switzerland) was used. A gradient (v/v) of [B] was employed as follows [t(min), %B]: 0.00, 2; 2.00, 2; 6.00, 50; 30.00, 100; 34.0, 100; followed by re-equilibration steps (36.00, 2; 40.00, 2). The collection was done at a fixed fraction volume of 1.600 mL per well (96-W 2 mL Deep Well Plate, Scientific Specialties Inc., California, United States). A 100 *µ*L aliquot of each well was pooled (row-wise and column-wise) in a preweighted vial and then dried under an N_2_ flux. After weighing, each pooled sample was reconstituted in DMSO at 5 mg/mL.

### Isolation of compounds from the HPE extract

The HPE (1.1 g) was separated on a Puriflash C18-HP column (200 × 30 mm I.D., 15 *μ*m, Interchim, Montlucon, France) on a Buchi system. The flow rate was set to 60 mL/min, and a binary solvent system consisting of 0.1% aqueous formic acid [A] and 0.1% formic acid in acetonitrile [B] was used. A gradient (v/v) of [B] was employed as follows [t(min), %B]: 0.00, 2; 3.70, 10; 73.50, 65; 84.00, 100; 94.00, 100. The collection was done by a fixed fraction volume of 50 mL per tube. After combining tubes based on the 254 nm and 280 nm UV traces, a total of 24 fractions were obtained. This separation yielded 31 mg of **1** (RT 74.0 min), 33.5 mg of **4** (RT 78.0 min), and 48.5 mg of **8** (RT 83.0 min). The fractions collected at RT 76.0 min (F13, 19.8 mg) and RT 81.0 min (F15, 23.0 mg) were separated in an X-Bridge C_18_ column (250 × 10 mm i. d., 5 *μ*m) equipped with a Waters C_18_ precolumn cartridge holder (5 × 10 mm i. d., 5 *μ*m); solvent system ACN B) and H_2_O A), both containing 0.1% FA, in an isocratic run 50% ACN, to give **2** (1.0 mg, RT 38.5 min), and **3** (0.6 mg, RT 42.0 min); and **6** (0.5 mg, RT 45.0 min) respectively. The following separations were done using the same solvent system and column but different isocratic compositions. The fraction F17 (RT 85.0 min, 48.4 mg) was separated using an isocratic of 55% ACN to give **5** (1.0 mg, RT 36.0 min), **8** (1.7 mg, RT 38.0 min), **9** (2.2 mg, RT 41.0 min) and **10** (2.3 mg, RT 43.5 min). The fractions F18 (RT 87.5 min, 25.7 mg), and F19 (RT 88.0 min, 26.9 mg) were separated using an isocratic of 60% ACN to give **7** (0.4 mg, RT 26 min), and **11** (1.7 mg, RT 41.0 min). Finally, the fraction F20 (RT 89.0 min, 27.7 mg) was separated using an isocratic of 60% ACN to give **12** (0.4 mg, RT 41.0 min), and **13** (0.5 mg, RT 43.0 min). All the fractions were introduced in the system using a Dry-load injection (Queiroz et al., 2019).

### Description of isolated compounds

Compound **1** (Hymenotamayonin G): (2*S*,8*R*,10*R*,17*R*)-2-(5-hydroxy, 4-methoxy-3-(3-methylbut-2-en-1-yl)phenyl)-12-(2-hydroxypropan-2-yl)-8-(3-methylbut-2-en-1-yl)-5-oxotricyclo[6.3.1.0^5,10^]dodec-5-ene-7,9-dione. Amorphous pale-yellow powder, ${\left[\alpha \right]}_{D}^{20}$ −16 (c 0.03, MeOH); UV (c 0.03, MeOH) λ_max_ 220, 269 nm.

^1^H NMR (CD_3_OD, 600 MHz) δ 1.21 (3H, s, H_3_-20), 1.34 (3H, s, H_3_-19), 1.48 (3H, d, *J* = 1.2 Hz, H_3_-14), 1.50 (3H, d, *J* = 1.2 Hz, H_3_-15), 1.64 (1H, overlapped, H-3eq), 1.73 (3H, d, *J* = 1.3 Hz, H_3_-4″), 1.77 (3H, d, *J* = 1.3 Hz, H_3_-5″), 1.91 (1H, dd, *J* = 12.9, 5.5 Hz, H-16b), 2.05 (1H, dd, *J* = 14.3, 6.0 Hz, H-11b), 2.16 (1H, overlapped, H-4eq), 2.17 (1H, dd, *J* = 14.3, 7.9 Hz, H-11a), 2.28 (2H, m, H-3ax, H-4ax), 2.82 (1H, t, *J* = 12.9, 10.9 Hz, H-16a), 3.12 (1H, d, *J* = 5.4 Hz, H-2), 3.28 (2H, m, H_2_-1″), 3.75 (3H, s, 4′-OCH_3_), 4.72 (1H, dd, *J* = 10.9, 5.5 Hz, H-17), 4.76 (1H, thept, *J* = 7.9, 6.0, 1.2 Hz, H-12), 5.24 (1H, thept, *J* = 6.7, 1.3 Hz, H-2″), 5.80 (1H, s, H-6), 6.38 (1H, d, *J* = 2.3 Hz, H-2′), 6.47 (1H, d, *J* = 2.3 Hz, H-6′); ^13^C NMR (CD_3_OD, 151 MHz) δ 17.9 (CH_3_-4″), 18.0 (CH_3_-14), 25.4 (CH_3_-20), 26.0 (CH_3_-5″), 26.1 (CH_3_-15), 26.5 (CH_3_-19), 27.5 (CH_2_-3), 29.1 (CH_2_-1″), 30.4 (CH_2_-11), 30.8 (CH_2_-16), 33.1 (CH_2_-4), 55.2 (CH-2), 60.8 (4′-OCH_3_), 61.7 (C-10), 67.2 (C-8), 71.1 (C-18), 92.8 (CH-17), 104.3 (CH-6), 115.8 (CH-6′), 121.1 (CH-12), 121.6 (CH-2′), 123.9 (CH-2″), 133.6 (C-3″), 134.1 (C-13), 136.3 (C-3′), 137.9 (C-1′), 146.0 (C-4′), 151.0 (C-5′), 181.2 (C-5), 201.3 (C-7), 207.7 (C-9). Supplementary Figures S8–S13. NP0332440.

^1^H NMR (CDCl_3_, 600 MHz) δ 1.25 (3H, s, H_3_-20), 1.38 (3H, s, H_3_-19), 1.51 (3H, s, H_3_-14), 1.55 (3H, s, H_3_-15), 1.67 (1H, overlapped, H-3eq), 1.73 (3H, s, H_3_-4″), 1.77 (3H, s, H_3_-5″), 1.85 (1H, dd, *J* = 13.0, 5.3 Hz, H-16b), 2.04 (1H, dd, *J* = 13.6, 4.9 Hz, H-4eq), 2.15 (1H, dd, *J* = 15.1, 5.7 Hz, H-11b), 2.25 (1H, td, *J* = 13.6, 4.9 Hz, H-4ax), 2.25(1H, dd, *J* = 15.1, 6.1 Hz, H-11a), 2.33 (1H, tt, *J* = 13.6, 6.1, 4.9 Hz, H-3ax), 2.83 (1H, t, *J* = 13.0, 11.1 Hz, H-16a), 3.24 (1H, d, *J* = 6.1 Hz, H-2), 3.30 (2H, d, *J* = 7.3 Hz, H_2_-1″), 3.77 (3H, s, 4′OCH3), 4.57 (1H, dd, *J* = 11.1, 5.3 Hz, H-17), 4.77 (1H, t, *J* = 6.1, 5.7 Hz, H-12), 5.23 (1H, t, *J* = 7.3 Hz, H-2″), 5.86 (1H, s, H-6), 6.41 (1H, d, *J* = 2.3 Hz, H-2′), 6.53 (1H, d, *J* = 2.3 Hz, H-6′); ^13^C NMR (CDCl_3_, 151 MHz) δ 17.9 (CH_3_-4″), 18.1 (CH_3_-14), 24.4 (CH_3_-20), 26.0 (CH_3_-5″, CH_3_-15), 26.6 (CH_2_-3), 27.0 (CH_3_-19), 28.3 (CH_2_-1″), 29.3 (CH_2_-11), 30.4 (CH_2_-16), 32.1 (CH_2_-4), 53.8 (CH-2), 60.1 (C-10), 61.3 (4′-OCH_3_), 66.0 (C-8), 70.9 (C-18), 91.0 (CH-17), 104.4 (CH-6), 113.1 (CH-6′), 119.7 (CH-12), 121.9 (CH-2″), 122.3 (CH-2″), 133.4 (C-3″), 133.7 (C-13), 134.5 (C-3′), 137.5 (C-1′), 144.2 (C-4′), 148.7 (C-5′), 177.7 (C-5), 198.8 (C-7) 206.4 (C-9). HRESIMS *m/z* 509.2894 [M + H]^+^ (calculated for C_31_H_41_O_6_, error −0.65 ppm); MS/MS CCMSLIB00011431737.

InChI = 1S/C31H40O6/c1-182)8-9-20-14-21(15-23(32)27(20)36-7)22-11-12-30-17-26(29(5,6)35)37-25(30)16-24(33)31(22,28(30)34)13-10-193)4/h8,10,14-16,22,26,32,35H,9,11-13,17H2,1-7H3/t22-,26+,30+,31+/m0/s1.

Compound **2** (Hymenotamayonin A): (2*S*,8*R*,10*R*)-2-(4,5-dihydroxy-3-(3-methylbut-2-en-1-yl)phenyl)-5-hydroxy-8,10-bis(3-methylbut-2-en-1-yl)bicyclo[3.3.1]non-6-ene-7,9-dione. Amorphous pale-yellow powder, [α]D20 -2 (c 0.001, MeOH); UV (c 0.001, MeOH) λ_max_ 213, 281 nm.

^1^H NMR (CD_3_OD, 600 MHz) δ 1.47 (3H, d, *J* = 1.5 Hz, H_3_-14), 1.55 (3H, d, *J* = 1.5 Hz, H_3_-15), 1.55 (1H, overlapped, H-3eq), 1.68 (3H, d, *J* = 1.5 Hz, H_3_-20), 1.70 (3H, d, *J* = 1.5 Hz, H_3_-19), 1.72 (3H, d, *J* = 1.4 Hz, H_3_-4″), 1.76 (3H, d, *J* = 1.4 Hz, H_3_-5″), 1.79 (1H, dd, *J* = 13.8, 5.2 Hz, H-4eq), 2.09 (2H, m, H_2_-11), 2.17 (1H, td, *J* = 13.8, 4.9 Hz, H-4ax), 2.34 (1H, tt, *J* = 14.3, 5.8 Hz, H-3ax), 2.47 (1H, dd, *J* = 14.5, 6.9 Hz, H-16b), 2.57 (1H, dd, *J* = 14.5, 6.9 Hz, H-16a), 3.07 (1H, d, *J* = 6.4 Hz, H-2eq), 3.25 (2H, m, H_2_-1″), 4.87 (1H, overlapped, H-12), 5.19 (1H, thept, *J* = 6.9, 1.5 Hz, H-17), 5.30 (1H, thept, *J* = 7.4, 1.4 Hz, H-2″), 6.28 (1H, d, *J* = 2.2 Hz, H-2′), 6.38 (1H, d, *J* = 2.2 Hz, H-6′); ^13^C NMR (CD_3_OD, 151 MHz) δ 17.9 (CH_3_-4″), 18.0 (CH_3_-14), 18.2 (CH_3_-19), 26.0 (CH_3_-5″), 26.1 (CH_3_-15), 26.1 (CH_3_-20), 28.1 (CH_2_-3), 29.2 (CH_2_-1″), 30.3 (CH_2_-11), 31.1 (CH_2_-16), 34.7 (CH_2_-4), 53.8 (CH-2), 61.6 (C-10), 64.7 (C-8), 114.8 (CH-6′), 121.4 (CH-2′), 121.9 (CH-17), 122.0 (CH-12), 123.9 (CH-2″), 129.4 (C-3′), 133.2 (C-3″, C-13), 133.4 (C-1′), 133.9 (C-18), 143.0 (C-4′), 145.8 (C-5′), 211.5 (C-9). Supplementary Figures S14–S19. NP0332433. HRESIMS *m/z* 479.2793 [M + H]^+^ (calculated for C_30_H_39_O_5_, error 0.21 ppm); MS/MS CCMSLIB00011431729.

InChI = 1S/C30H38O5/c1-18(2)7-8-21-15-22(16-24(31)27(21)34)23-11-13-29(12-9-19(3)4)25(32)17-26(33)30(23,28(29)35)14-10-20(5)6/h7,9-10,15-17,23,31-32,34H,8,11-14H2,1-6H3/t23-,29-,30+/m0/s1.

Compound **3**: 3′,8-diprenylapigenin (Dat et al., 2010). Amorphous white powder, [α]D20 -14 (c 0.0006, MeOH); UV (c 0.0006, MeOH) λ_max_ 203, 276, 349 nm.

^1^H NMR (CD_3_OD, 600 MHz) δ 1.68 (3H, s, H_3_-5″), 1.74 (3H, s, H_3_-10′), 1.78 (3H, s, H_3_-11′), 1.81 (3H, s, H_3_-4″), 3.37 (2H, d, *J* = 7.4 Hz, H_2_-7′), 3.53 (2H, d, *J* = 7.2 Hz, H_2_-1″), 5.29 (1H, t, *J* = 7.2 Hz, H-2″), 5.36 (1H, t, *J* = 7.4 Hz, H-8′), 6.26 (1H, s, H-6), 6.57 (1H, s, H-3), 6.90 (1H, d, *J* = 8.3 Hz, H-5′), 7.69 (1H, dd, *J* = 8.3, 2.9 Hz, H-6′), 7.76 (1H, d, *J* = 2.6 Hz, H-2′); ^13^C NMR (CD_3_OD, 151 MHz) δ 17.9 (CH_3_-10′), 18.2 (CH_3_-4″), 22.6 (CH_2_-1″), 25.9 (CH_3_-11′), 26.0 (CH_3_-5″), 29.0 (CH_2_-7′), 99.6 (CH-6), 103.3 (CH-3), 105.2 (C-10), 108.2 (C-8), 116.2 (CH-5′), 123.0 (CH-8′), 123.4 (C-1′), 123.6 (CH-2″), 126.9 (CH-6′), 128.8 (CH-2′), 130.3 (C-3′), 132.7 (C-3″), 134.2 (C-9′), 156.5 (C-9), 160.5 (C-4′), 160.8 (C-5), 163.4 (C-7), 166.5 (C-2), 184.3 (C-4). Supplementary Figures S20–S25 NP0332434. HRESIMS *m/z* 407.1853 [M + H]^+^ (calculated for C_25_H_39_O_5_, error 0.14 ppm); MS/MS CCMSLIB00011431727.

InChI = 1S/C25H26O5/c1-142)5-7-16-11-17(8-10-19(16)26)23-13-22(29)24-21(28)12-20(27)18(25(24)30-23)9-6-153)4/h5-6,8,10-13,26-28H,7,9H2,1-4H3.

Compound **4** (Hymenotamayonin H): (2*S*,8*R*,10*R*,12*S*)-2-(5-hydroxy, 4-methoxy-3-(3-methylbut-2-en-1-yl)phenyl)-17-(2-hydroxypropan-2-yl)-10-(3-methylbut-2-en-1-yl-7-oxotricyclo[6.3.1.0^7,8^]dodec-6-ene-5,9-dione. Amorphous pale yellow powder, [α]D20 + 3 (c 0.03, MeOH); UV (c 0.03, MeOH) λ_max_ 214, 269 nm.

^1^H NMR (CD_3_OD, 600 MHz) δ 1.10 (3H, s, H_3_-15), 1.28 (3H, s, H_3_-14), 1.63 (3H, s, H_3_-20), 1.67 (1H, overlapped, H-3eq), 1.68 (3H, s, H-_3_19), 1.73 (3H, s, H_3_-4″), 1.74 (1H, overlapped, H-11b), 1.76 (3H, s, H_3_-5″), 1.78 (1H, overlapped, H-4eq), 2.08 (1H, td, *J* = 14.1, 4.8 Hz, H-4ax), 2.36 (1H, tt, *J* = 15.0, 14.1, 6.4, 5.0 Hz, H-3ax), 2.43 (1H, dd, *J* = 14.5, 6.9 Hz, H-16b), 2.53 (2H, m, H-11a, H-16a), 3.28 (2H, d, *J* = 7.4 Hz, H_2_-1″), 3.40 (1H, d, *J* = 6.4 Hz, H-2), 3.74 (3H, s, 4′-OCH_3_), 4.62 (1H, dd, *J* = 11.1, 5.4 Hz, H-12), 5.07 (1H, brt, *J* = 7.3 Hz, H-17), 5.23 (2H, brt, *J* = 7.4 Hz, H-2″), 5.81 (1H, s, H-6), 6.43 (1H, d, *J* = 2.3 Hz, H-2′), 6.53 (1H, d, *J* = 2.3 Hz, H-6′); ^13^C NMR (CD_3_OD, 151 MHz) δ 18.0 (CH_3_-4″), 18.2 (CH_3_-19), 25.2 (CH_3_-15), 26.0 (CH_3_-5″), 26.1 (CH_3_-20), 26.3 (CH_3_-14), 27.8 (CH_2_-3), 28.7 (CH_2_-11), 29.2 (CH_2_-1″), 31.1 (CH_2_-16), 34.9 (CH_2_-4), 49.4 (CH-2), 60.8 (4′-OCH_3_), 64.4 (C-10), 64.7 (C-8), 71.1 (C-13), 92.2 (CH-12), 104.7 (CH-6), 115.9 (CH-6′), 121.1 (CH-17), 121.3 (CH-2′), 123.8 (CH-2″), 133.6 (C-3″), 134.5 (C-18), 136.7 (C-3′), 138.1 (C-1′), 146.1 (C-4′), 151.4 (C-5′), 182.1 (C-7), 200.7 (C-5), 208.0 (C-9). Supplementary Figures S26–S31. NP0332430. HRESIMS *m/z* 509.2894 [M + H]^+^ (calculated for C_31_H_41_O_6_, error −0.65 ppm); MS/MS CCMSLIB00011431736.

InChI = 1S/C31H40O6/c1-18(2)8-9-20-14-21(15-23(32)27(20)36-7)22-11-13-30(12-10-19(3)4)24(33)16-25-31(22,28(30)34)17-26(37-25)29(5,6)35/h8,10,14-16,22,26,32,35H,9,11-13,17H2,1-7H3/t22-,26-,30-,31+/m0/s1.

Compound **5**: 6,8-diprenylapigenin (Lin et al., 2017). Amorphous white powder, [α]D20 -15 (c 0.001, MeOH); UV (c 0.001, MeOH) λ_max_ 207, 281, 337 nm.

^1^H NMR (CD_3_OD, 600 MHz) δ 1.68 (3H, d, *J* = 1.4 Hz, H_3_-5″), 1.70 (3H, d, *J* = 1.5 Hz, H_3_-5‴), 1.80 (3H, d, *J* = 1.4 Hz, H_3_-4″), 1.84 (3H, d, *J* = 1.5 Hz, H_3_-4‴), 3.39 (2H, d, *J* = 7.2 Hz, H_2_-1″), 3.60 (2H, d, *J* = 6.6 Hz, H_2_-1‴), 5.21 (2H, m, H-2″, H-2‴), 6.60 (1H, s, H-3), 6.93 (2H, d, *J* = 8.8 Hz, H-3′, H-5′), 7.85 (2H, d, *J* = 8.8 Hz, H-2′, H-6′); ^13^C NMR (CD_3_OD, 151 MHz) δ 18.0 (CH_3_-4″), 18.3 (CH_3_-4‴), 22.5 (CH_2_-1″), 23.0 (CH_2_-1‴), 25.9 (CH_3_-5‴), 26.0 (CH_3_-5″), 103.6 (CH-3), 105.5 (C-10), 108.2 (C-8), 113.3 (C-6), 117.0 (CH-3′, CH-5′), 123.2 (CH-2″), 123.7 (C-1′), 123.9 (CH-2‴), 129.5 (CH-2′, CH-6′), 132.8 (C-3″), 133.0 (C-3‴), 154.6 (C-9), 157.9 (C-5), 160.8 (C-7), 162.7 (C-4′), 166.1 (C-2), 184.4 (C-4). Supplementary Figures S32–S37. NP0332439. HRESIMS *m/z* 407.1853 [M + H]^+^ (calculated for C_25_H_27_O_5_, error 0.14 ppm); MS/MS CCMSLIB00011431739.

InChI = 1S/C25H26O5/c1-14(2)5-11-18-23(28)19(12-6-15(3)4)25-22(24(18)29)20(27)13-21(30-25)16-7-9-17(26)10-8-16/h5-10,13,26,28-29H,11-12H2,1-4H3.

Compound **6** (Hymenotamayonin I): (2*S*,8*R*,10*R*,17*R*)-17-hydroxy-2-(5-hydroxy, 4-methoxy-3-(3-methylbut-2-en-1-yl)phenyl)-18,18-dimethyl-8-(3-methylbut-2-en-1-yl)-5-oxotricyclo[7.3.1.0^5,10^]dodec-5-ene-7,9-dione. Amorphous pale yellow powder, [α]D20 + 18 (c 0.0005, MeOH); UV (c 0.0005, MeOH) λ_max_ 207, 273 nm.

^1^H NMR (CD_3_OD, 600 MHz) δ 1.26 (3H, s, H_3_-20), 1.44 (3H, s, H_3_-19), 1.47 (3H, d, *J* = 1.4 Hz, H_3_-14), 1.53 (3H, d, *J* = 1.4 Hz, H_3_-15), 1.55 (1H, brd, *J* = 10.8 Hz, H-3eq), 1.74 (1H, dd, *J* = 14.9, 5.3 Hz, H-16b), 1.74 (3H, d, *J* = 1.3 Hz, H_3_-4″), 1.79 (3H, d, *J* = 1.3 Hz, H_3_-5″), 2.09 (1H, td, *J* = 14.5, 4.6 Hz, H-4ax), 2.07 (1H, overlapped, H-11b), 2.15 (1H, dd, *J* = 14.7, 7.8 Hz, H-11a), 2.26 (1H, tt, *J* = 14.2, 5.6 Hz, H-3ax), 2.63 (1H, dd, *J* = 14.5, 4.9 Hz, H-4eq), 2.94 (1H, dd, *J* = 14.9, 3.9 Hz, H-16a), 3.03 (1H, d, *J* = 6.2 Hz, H-2eq), 3.28 (2H, d, *J* = 7.5 Hz, H_2_-1″), 3.75 (3H, s, 4′-OCH_3_), 3.79 (1H, t, *J* = 5.3, 3.9 Hz, H-17), 4.81 (1H, thept, *J* = 7.8, 1.4 Hz, H-12), 5.25 (1H, thept, *J* = 7.5, 1.3 Hz, H-2″), 5.88 (1H, s, H-6), 6.31 (1H, d, *J* = 2.3 Hz, H-2′), 6.42 (1H, d, *J* = 2.3 Hz, H-6′); ^13^C NMR (CD_3_OD, 151 MHz) δ 17.9 (CH_3_-4″), 18.0 (CH_3_-14), 24.0 (CH_3_-19), 26.1 (CH_3_-5″), 26.1 (CH_3_-15), 26.9 (CH_3_-20), 27.3 (CH_2_-3), 29.0 (CH_2_-1″), 30.5 (CH_2_-11), 31.5 (CH_2_-16), 36.2 (CH_2_-4), 51.6 (C-10), 55.0 (CH-2), 60.8 (4′-OCH_3_), 67.5 (C-8), 69.9 (CH-17), 84.8 (C-18), 114.7 (CH-6), 116.1 (CH-6′), 121.2 (CH-12), 121.4 (CH-2′), 123.8 (CH-2″), 133.8 (C-3″), 134.0 (C-13), 136.2 (C-3′), 138.1 (C-1′), 145.9 (C-4′), 151.0 (C-5′), 176.5 (C-5), 200.7 (C-7), 209.7 (C-9). Supplementary Figures S38–43. NP0332438. HRESIMS *m/z* 509.2894 [M + H]^+^ (calculated for C_31_H_41_O_6_, error −0.65 ppm); MS/MS CCMSLIB00011431735.

InChI = 1S/C31H40O6/c1-18(2)8-9-20-14-21(15-23(32)27(20)36-7)22-11-12-30-17-25(34)29(5,6)37-26(30)16-24(33)31(22,28(30)35)13-10-19(3)4/h8,10,14-16,22,25,32,34H,9,11-13,17H2,1-7H3/t22-,25+,30+,31+/m0/s1.

Compound **7**: 3′,6-diprenyl-diprenylapigenin (Fukai et al., 1991). Amorphous white powder, [α]D20 -23 (c 0.0004, MeOH); UV (c 0.0004, MeOH) λ_max_ 206, 275, 343 nm.

^1^H NMR (CD_3_OD, 600 MHz) δ 1.67 (3H, d, *J* = 1.4 Hz, H_3_-5″), 1.76 (3H, d, *J* = 1.3 Hz, H_3_-10′), 1.79 (6H, d, *J* = 1.4 Hz, H_3_-4″, H_3_-11′), 3.32 (2H, overlapped, H_2_-1″), 3.36 (2H, d, *J* = 7.4 Hz, H_2_-7′), 5.24 (1H, thept, *J* = 7.0, 1.4 Hz, H-2″), 5.36 (1H, thept, *J* = 7.4, 1.3 Hz, H-8′), 6.48 (1H, s, H-8), 6.55 (1H, s, H-3), 6.89 (1H, d, *J* = 9.0 Hz, H-3′), 7.67 (2H, m, H-2′, H-6′); ^13^C NMR (CD_3_OD, 151 MHz) δ 17.9 (CH_3_-4″, CH_3_-10′), 22.3 (CH_2_-1″), 25.8 (CH_3_-11′), 26.0 (CH_3_-5″), 29.2 (CH_2_-7′), 94.1 (CH-8), 103.6 (CH-3), 105.1 (C-10), 113.1 (C-6), 116.2 (CH-3′), 123.2 (CH-8′, C-1′), 123.4 (CH-2″), 126.8 (CH-2′), 128.9 (CH-6′), 130.4 (C-5′), 132.2 (C-3″), 134.0 (C-9′), 157.4 (C-9), 159.9 (C-5), 160.5 (C-4′), 163.7 (C-7), 166.4 (C-2), 184.1 (C-4). Supplementary Figures S44–48; NP0332428. HRESIMS *m/z* 407.1853 [M + H]^+^ (calculated for C_25_H_27_O_5_, error 0.22 ppm); MS/MS CCMSLIB00011431728.

InChI = 1S/C25H26O5/c1-142)5-7-16-11-17(8-10-19(16)26)22-13-21(28)24-23(30-22)12-20(27)18(25(24)29)9-6-153)4/h5-6,8,10-13,26-27,29H,7,9H2,1-4H3.

Compound **8** (Hymenotamayonin C): (2*S*,8*R*,10*R*)-2-(5-hydroxy-4-methoxy-3-(3-methylbut-2-en-1-yl)phenyl)-5-hydroxy-8,10-bis(3-methylbut-2-en-1-yl)bicyclo[3.3.1]non-6-ene-7,9-dione. Amorphous orange powder, [α]D20 -14 (c 0.001, MeOH); UV (c 0.001, MeOH) λ_max_ 219, 271 nm.

^1^H NMR (CD_3_OD, 600 MHz) δ 1.46 (3H, d, *J* = 1.5 Hz, H_3_-14), 1.55 (3H, d, *J* = 1.5 Hz, H_3_-15), 1.56 (1H, overlapped, H-3eq), 1.68 (3H, d, *J* = 1.4 Hz, H_3_-19), 1.70 (3H, d, *J* = 1.4 Hz, H_3_-20), 1.74 (3H, d, *J* = 1.4 Hz, H_3_-4″), 1.76 (3H, d, *J* = 1.4 Hz, H_3_-5″), 1.79 (1H, overlapped, H-4eq), 2.08 (1H, dd, *J* = 14.2, 5.2 Hz, H-11b), 2.15 (1H, overlapped, H-11a), 2.17 (1H, td, *J* = 13.8, 4.7 Hz, H-4ax), 2.31 (1H, tt, *J* = 13.8, 6.4 Hz, H-3ax), 2.49 (1H, dd, *J* = 14.5, 7.1 Hz, H-16b), 2.58 (1H, dd, *J* = 14.5, 7.1 Hz, H-16a), 3.12 (1H, d, *J* = 6.4 Hz, H-2eq), 3.27 (2H, m, H_2_-1″), 3.74 (3H, s, 4′-OCH_3_), 4.87 (1H, overlapped, H-12), 5.19 (1H, thept, *J* = 7.1, 1.5 Hz, H-17), 5.25 (1H, thept, *J* = 7.5, 1.4 Hz, 2″), 6.34 (1H, d, *J* = 2.3 Hz, H-2′), 6.44 (1H, d, *J* = 2.3 Hz, H-6′); ^13^C NMR (CD_3_OD, 151 MHz) δ 18.0 (CH_3_-4″, CH_3_-14), 18.2 (CH_3_-19), 26.0 (CH_3_-5″), 26.1 (CH_3_-15), 26.2 (CH_3_-20), 27.8 (CH_2_-3), 29.3 (CH_2_-1″), 30.1 (CH_2_-11), 30.9 (CH_2_-16), 34.5 (CH_2_-4), 53.6 (CH-2), 60.8 (4′-OCH_3_), 116.2 (CH-6′), 121.4 (CH-12, CH-17), 121.6 (CH-2′), 124.0 (CH-2″), 133.4 (C-3″), 133.8 (C-13), 134.4 (C-18), 136.2 (C-3′), 138.2 (C-1′), 145.9 (C-4′), 150.9 (C-5′), 210.4 (C-9). Supplementary Figures S49–54. NP0332429. HRESIMS *m/z* 493.2894 [M + H]^+^ (calculated for C_31_H_41_O_5_, error −0.34 ppm); MS/MS CCMSLIB00011431733.

InChI = 1S/C31H40O5/c1-19(2)8-9-22-16-23(17-25(32)28(22)36-7)24-12-14-30(13-10-20(3)4)26(33)18-27(34)31(24,29(30)35)15-11-21(5)6/h8,10-11,16-18,24,32-33H,9,12-15H2,1-7H3/t24-,30-,31+/m0/s1.

Compound **9** (Hymenotamayonin J): (2*S*,8*R*,10*R*,12*S*)-12-hydroxy-2-(5-hydroxy, 4-methoxy-3-(3-methylbut-2-en-1-yl)phenyl)-13,13-dimethyl-10-(3-methylbut-2-en-1-yl)-7-oxotricyclo[7.3.1.0^7,8^]dodec-5-ene-5,9-dione. Amorphous orange powder, [α]D20 -20 (c 0.002, MeOH); UV (c 0.002, MeOH) λ_max_ 217, 276, 344 nm.

^1^H NMR (CD_3_OD, 600 MHz) δ 1.46 (3H, d, *J* = 1.5 Hz, H_3_-14), 1.55 (3H, d, *J* = 1.5 Hz, H_3_-15), 1.56 (1H, overlapped, H-3eq), 1.68 (3H, d, *J* = 1.4 Hz, H_3_-19), 1.70 (3H, d, *J* = 1.4 Hz, H_3_-20), 1.74 (3H, d, *J* = 1.4 Hz, H_3_-4″), 1.76 (3H, d, *J* = 1.4 Hz, H_3_-5″), 1.79 (1H, overlapped, H-4eq), 2.08 (1H, dd, *J* = 14.2, 5.2 Hz, H-11b), 2.15 (1H, overlapped, H-11a), 2.17 (1H, td, *J* = 13.8, 4.7 Hz, H-4ax), 2.31 (1H, tt, *J* = 13.8, 6.4 Hz, H-3ax), 2.49 (1H, dd, *J* = 14.5, 7.1 Hz, H-16b), 2.58 (1H, dd, *J* = 14.5, 7.1 Hz, H-16a), 3.12 (1H, d, *J* = 6.4 Hz, H-2eq), 3.27 (2H, m, H_2_-1″), 3.74 (3H, s, 4′-OCH_3_), 4.87 (1H, overlapped, H-12), 5.19 (1H, thept, *J* = 7.1, 1.5 Hz, H-17), 5.25 (1H, thept, *J* = 7.5, 1.4 Hz, 2″), 6.34 (1H, d, *J* = 2.3 Hz, H-2′), 6.44 (1H, d, *J* = 2.3 Hz, H-6′); ^13^C NMR (CD_3_OD, 151 MHz) δ 18.0 (CH_3_-4″, CH_3_-14), 18.2 (CH_3_-19), 26.0 (CH_3_-5″), 26.1 (CH_3_-15), 26.2 (CH_3_-20), 27.8 (CH_2_-3), 29.3 (CH_2_-1″), 30.1 (CH_2_-11), 30.9 (CH_2_-16), 34.5 (CH_2_-4), 53.6 (CH-2), 60.8 (4′-OCH_3_), 116.2 (CH-6′), 121.4 (CH-12, CH-17), 121.6 (CH-2′), 124.0 (CH-2″), 133.4 (C-3″), 133.8 (C-13), 134.4 (C-18), 136.2 (C-3′), 138.2 (C-1′), 145.9 (C-4′), 150.9 (C-5′), 210.4 (C-9). Supplementary Figures S55–S60. NP0332431. HRESIMS *m/z* 509.2893 [M + H]^+^ (calculated for C_31_H_41_O_6_, error −0.83 ppm); MS/MS CCMSLIB00011431738.

InChI = 1S/C31H40O6/c1-18(2)8-9-20-14-21(15-23(32)27(20)36-7)22-11-13-30(12-10-19(3)4)24(33)16-26-31(22,28(30)35)17-25(34)29(5,6)37-26/h8,10,14-16,22,25,32,34H,9,11-13,17H2,1-7H3/t22-,25-,30-,31+/m0/s1.

Compound **10** (Hymenotamayonin B): (2*R*,8*R*,10*R*)-2-(4,5-dihydroxy-3-(3-methylbut-2-en-1-yl)phenyl)-5-hydroxy-8,10-bis(3-methylbut-2-en-1-yl)bicyclo[3.3.1]non-6-ene-7,9-dione. Amorphous orange powder, [α]D20 -12 (c 0.002, MeOH); UV (c 0.002, MeOH) λ_max_ 221, 273 nm.

^1^H NMR (CD_3_OD, 600 MHz) δ 1.51 (3H, d, *J* = 1.5 Hz, H_3_-14), 1.59 (3H, d, *J* = 1.5 Hz, H_3_-15), 1.63 (3H, d, *J* = 1.5 Hz, H_3_-20), 1.64 (1H, overlapped, H-4ax), 1.69 (3H, d, *J* = 1.5 Hz, H_3_-19), 1.71 (3H, d, *J* = 1.4 Hz, H_3_-4″), 1.71 (1H, overlapped, H-3eq), 1.73 (3H, d, *J* = 1.4 Hz, H_3_-5″), 1.97 (1H, ddd, *J* = 13.1, 4.9, 1.8 Hz, H-4eq), 2.21 (2H, m, H-3ax, H-11b), 2.30 (1H, dd, *J* = 15.0, 6.7 Hz, H-11a), 2.48 (2H, m, H_2_-16), 2.64 (1H, dd, *J* = 13.2, 4.0 Hz, H-2ax), 3.23 (1H, dd, *J* = 15.6, 7.4 Hz, H-1″b), 3.28 (1H, dd, *J* = 15.6, 7.4 Hz, H-1″a), 4.87 (1H, overlapped, H-12), 4.97 (1H, thept, *J* = 6.9, 1.5 Hz, H-17), 5.29 (1H, thept, *J* = 7.4, 1.4 Hz, H-2″), 6.32 (1H, d, *J* = 2.2 Hz, H-2′), 6.40 (1H, d, *J* = 2.2 Hz, H-6′); ^13^C NMR (CD_3_OD, 151 MHz) δ 17.9 (CH_3_-4″), 18.2 (CH_3_-14, CH_3_-19), 26.0 (CH_3_-5″), 26.1 (CH_3_-15), 26.1 (CH_3_-20), 28.1 (CH_2_-3), 29.2 (CH_2_-1″), 29.8 (CH_2_-11), 30.9 (CH_2_-16), 39.2 (CH_2_-4), 56.2 (CH-2), 114.4 (CH-6′), 121.4 (CH-17), 121.9 (CH-12), 122.6 (CH-2′), 124.2 (CH-2″), 128.7 (C-3′), 131.3 (C-1′), 132.7 (C-3″), 133.3 (C-13), 134.0 (C-18), 143.4 (C-4′), 145.3 (C-5′), 211.0 (C-9). Supplementary Figures S61–66. NP0332436. HRESIMS *m/z* 479.2791 [M + H]^+^ (calculated for C_30_H_39_O_5_, error −0.19 ppm); MS/MS CCMSLIB00011431730.

InChI = 1S/C30H38O5/c1-182)7-8-21-15-22(16-24(31)27(21)34)23-11-13-29(12-9-193)4)25(32)17-26(33)30(23,28(29)35)14-10-205)6/h7,9-10,15-17,23,31-32,34H,8,11-14H2,1-6H3/t23-,29+,30-/m1/s1.

Compound **11** (Hymenotamayonin D): (2*R*,8*R*,10*R*)-2-(5-hydroxy-4-methoxy-3-(3-methylbut-2-en-1-yl)phenyl)-5-hydroxy-8,10-bis(3-methylbut-2-en-1-yl)bicyclo[3.3.1]non-6-ene-7,9-dione. Amorphous pale yellow powder, [α]D20 -27 (c 0.0008, MeOH); UV (c 0.0008, MeOH) λ_max_ 207, 286 nm.

^1^H NMR (CD_3_OD, 600 MHz) δ 1.51 (3H, d, *J* = 1.6 Hz, H_3_-14), 1.60 (3H, d, *J* = 1.6 Hz, H_3_-15), 1.63 (3H, d, *J* = 1.3 Hz, H_3_-20), 1.63 (1H, overlapped, H-4ax), 1.69 (3H, d, *J* = 1.3 Hz, H_3_-19), 1.71 (1H, overlapped, H-3eq), 1.73 (6H, d, *J* = 1.3 Hz, H_3_-4″, H_3_-5″), 1.98 (1H, ddd, *J* = 12.9, 4.8, 1.8 Hz, H-4eq), 2.18 (1H, dd, *J* = 14.9, 7.2 Hz, H-11b), 2.25 (1H, td, *J* = 13.1, 4.8 Hz, H-3ax), 2.32 (1H, dd, *J* = 14.9, 7.2 Hz, H-11a), 2.46 (1H, dd, *J* = 14.3, 7.0 Hz, H-16b), 2.50 (1H, dd, *J* = 14.3, 7.0 Hz, 16a), 2.68 (1H, dd, *J* = 13.1, 4.0 Hz, H-2ax), 3.27 (2H, m, H_2_-1″), 3.73 (3H, s, 4′-OCH_3_), 4.89 (1H, overlapped, H-12), 4.98 (1H, thept, *J* = 7.0, 1.6 Hz, H-17), 5.24 (1H, thept, *J* = 7.2, 1.3 Hz, H-2″), 6.38 (1H, d, *J* = 2.2 Hz, H-2′), 6.47 (1H, d, *J* = 2.2 Hz, H-6′); ^13^C NMR (MeOD, 151 MHz) δ 18.0 (CH_3_-4″), 18.2 (CH_3_-14, CH_3_-19), 25.9 (CH_3_-5″), 26.1 (CH_3_-15), 26.1 (CH_3_-20), 28.2 (CH_2_-3), 29.4 (CH_2_-1″), 29.7 (CH_2_-11), 31.0 (CH_2_-16), 39.0 (CH_2_-4), 55.7 (CH-2), 60.7 (4′-OCH_3_), 116.0 (CH-6′), 121.6 (CH-17), 122.0 (CH-12), 122.9 (CH-2′), 124.5 (CH-2″), 132.9 (C-3″), 133.1 (C-13), 134.1 (C-18), 135.4 (C-3′), 136.6 (C-1′), 146.2 (C-4′), 150.4 (C-5′), 211.1 (C-9). Supplementary Figures S67–72. NP0332437. HRESIMS *m/z* 493.2947 [M + H]^+^ (calculated for C_31_H_41_O_5_, error −0.28 ppm); MS/MS CCMSLIB00011431732.

InChI = 1S/C31H40O5/c1-192)8-9-22-16-23(17-25(32)28(22)36-7)24-12-14-30(13-10-203)4)26(33)18-27(34)31(24,29(30)35)15-11-215)6/h8,10-11,16-18,24,32-33H,9,12-15H2,1-7H3/t24-,30+,31-/m1/s1.

Compound **12** (Hymenotamayonin F): (2*S*,8*R*,10*R*)-2-(5-hydroxy, 4-methoxy-3-(3-methylbut-2-en-1-yl)phenyl)-18,18-dimethyl-8-(3-methylbut-2-en-1-yl)-5-oxotricyclo[7.3.1.0^5,10^]dodec-5-ene-7,9-dione. Amorphous pale yellow powder, [α]D20 + 6 (c 0.0004, MeOH); UV (c 0.0004, MeOH) λ_max_ 205, 273 nm.

^1^H NMR (CD_3_OD, 600 MHz) δ 1.29 (3H, s, H_3_-20), 1.46 (3H, s, H_3_-19), 1.47 (3H, d, *J* = 1.3 Hz, H_3_-14), 1.54 (3H, d, *J* = 1.3 Hz, H_3_-15), 1.63 (1H, m, H-3eq), 1.71 (1H, dt, *J* = 14.4, 4.2 Hz, H-16eq), 1.73 (3H, d, *J* = 1.3 Hz, H_3_-4″), 1.78 (3H, d, *J* = 1.3 Hz, H_3_-5″), 1.86 (1H, dt, *J* = 14.3, 4.1 Hz, H-17eq), 1.96 (1H, td, *J* = 13.3, 4.5 Hz, H-4ax), 2.04 (1H, td, *J* = 14.3, 4.1 Hz, H-17ax), 2.06 (1H, dd, *J* = 14.7, 6.1 Hz, H-11b), 2.16 (1H, dd, *J* = 14.7, 7.8 Hz, H-11a), 2.23 (1H, m, H-3ax), 2.28 (1H, dd, *J* = 13.3, 4.5 Hz, H-4eq), 2.56 (1H, td, *J* = 14.4, 4.2 Hz, H-16ax), 3.04 (1H, d, *J* = 6.2 Hz, H-2eq), 3.28 (2H, m, H_2_-1″), 3.74 (3H, s, 4′-OCH_3_), 4.83 (1H, thept, *J* = 7.6, 1.3 Hz, H-12), 5.24 (1H, thept, *J* = 7.5, 1.3 Hz, H-2″), 5.82 (1H, s, H-6), 6.31 (1H, d, *J* = 2.3 Hz, H-2′), 6.41 (1H, d, *J* = 2.3 Hz, H-6′); ^13^C NMR (CD_3_OD, 151 MHz) δ 17.9 (CH_3_-14), 18.0 (CH_3_-4″), 24.2 (CH_2_-16), 26.0 (CH_3_-15), 26.1 (CH_3_-5″), 26.2 (CH_3_-20), 27.6 (CH_2_-3), 29.0 (CH_2_-1″), 30.1 (CH_3_-19), 30.3 (CH_2_-11), 31.2 (CH_2_-17), 33.2 (CH_2_-4), 51.0 (C-10), 54.7 (CH-2), 60.8 (4′-OCH_3_), 67.8 (C-8), 82.3 (C-18), 113.8 (CH-6), 115.9 (CH-6′), 121.3 (CH-12), 121.4 (CH-2′), 123.8 (CH-2″), 134.0 (C-3″), 136.3 (C-3′), 138.1 (C-1′), 145.9 (C-4′), 151.0 (C-5′), 176.8 (C-5), 200.4 (C-7), 210.3 (C-9). Supplementary Figures S73–S78. NP0332435. HRESIMS *m/z* 491.2747 [M + H]^+^ (calculated for C_31_H_41_O_5_, error −0.16 ppm); MS/MS CCMSLIB00011431734.

InChI = 1S/C31H40O5/c1-19(2)8-9-21-16-22(17-24(32)27(21)35-7)23-11-12-30-15-14-29(5,6)36-26(30)18-25(33)31(23,28(30)34)13-10-20(3)4/h8,10,16-18,23,32H,9,11-15H2,1-7H3/t23-,30+,31+/m0/s1.

Compound **13** (Hymenotamayonin E): (2*S*,8*R*,10*R*,17*R*)-2-(5-hydroxy, 4-methoxy-3-(3-methylbut-2-en-1-yl)phenyl)-8-(3-methylbut-2-en-1-yl)-17-(prop-1-en-2-yl-5-oxotricyclo[6.3.1.0^5,10^]dodec-5-ene-7,9-dione. Amorphous pale yellow powder, [α]D20 + 0.4 (c 0.0005, MeOH); UV (c 0.0005, MeOH) λ_max_ 204, 270 nm.

^1^H NMR (CD_3_OD, 600 MHz) δ 1.49 (3H, d, *J* = 1.4 Hz, H_3_-14), 1.53 (3H, d, *J* = 1.4 Hz, H_3_-15), 1.66 (1H, m, H-3eq), 1.73 (3H, d, *J* = 1.3 Hz, H_3_-4″), 1.77 (3H, d, *J* = 1.3 Hz, H_3_-5″), 1.78 (3H, t, *J* = 1.2 Hz, H_3_-19), 2.07 (1H, dd, *J* = 14.5, 6.0 Hz, H-11b), 2.12 (1H, dd, *J* = 13.0, 5.4 Hz, H-16b), 2.17 (1H, dd, *J* = 14.5, 7.7 Hz, H-11a), 2.23 (1H, brd, *J* = 9.4 Hz, H-4eq), 2.30 (1H, overlapped, H-4ax), 2.32 (1H, overlapped, H-3ax), 2.63 (1H, dd, *J* = 13.0, 11.2 Hz, 16a), 3.14 (1H, d, *J* = 3.8 Hz, H-2eq), 3.75 (3H, s, 4′-OCH_3_), 4.78 (1H, ddhept, *J* = 7.7, 6.0, 1.4 Hz, H-12), 5.05 (1H, p, *J* = 1.2 Hz, 20b), 5.17 (1H, q, *J* = 1.2 Hz, 20a), 5.25 (2H, thept, *J* = 7.5, 1.3 Hz, 2″), 5.32 (1H, dd, *J* = 11.2, 5.4 Hz, H-17), 5.80 (1H, s, H-6), 6.38 (1H, d, *J* = 2.3 Hz, H-2′), 6.47 (1H, d, *J* = 2.3 Hz, H-6′); ^13^C NMR (CD_3_OD, 151 MHz) δ 17.3 (CH_3_-19), 17.9 (CH_3_-14), 18.0 (CH_3_-4″), 26.0 (CH_3_-15), 26.1 (CH_3_-5″), 27.6 (CH_2_-3), 29.1 (CH_2_-1″), 30.5 (CH_2_-11), 32.6 (CH_2_-4), 35.4 (CH_2_-16), 55.3 (CH-2), 60.8 (4′-OCH_3_), 61.7 (C-10), 67.3 (C-8), 89.2 (CH-17), 104.2 (CH-6), 114.4 (CH_2_-20), 115.8 (CH-6′), 121.0 (CH-12), 121.6 (CH-2′), 123.9 (CH-2″), 133.5 (C-3″), 134.2 (C-13), 136.4 (C-3′), 137.9 (C-1′), 143.1 (C-18), 146.0 (C-4′), 151.1 (C-5′), 180.9 (C-5), 201.3 (C-7), 207.8 (C-9). Supplementary Figures S79–S84. NP0332432. HRESIMS *m/z* 491.2792 [M + H]^+^ (calculated for C_31_H_39_O_5_, error 0.08 ppm); MS/MS CCMSLIB00011431731.

InChI = 1S/C31H38O5/c1-182)8-9-21-14-22(15-24(32)28(21)35-7)23-11-12-30-17-25(205)6)36-27(30)16-26(33)31(23,29(30)34)13-10-193)4/h8,10,14-16,23,25,32H,5,9,11-13,17H2,1-4,6-7H3/t23-,25+,30+,31+/m0/s1.

### Electronic circular dichroism calculations (ECD)

The absolute configuration assigned for all compounds was based on a comparison between the calculated and experimental ECD. The calculations were based on the relative configuration determined through NMR 2D ROESY experiments. The structures were used to find the conformers through a random rotor search algorithm (number of conformers, 100) employing the MMFF94s force field in Avogrado *v*1.2.0 (Hanwell et al., 2012). The conformers were further optimized using PM3 and B3LYP/6-31G(d,p) basis sets in Gaussian 16 software (^©^ 2015-2022, Gaussian Inc., Wallingford, CT, United States of America) with the SCRF model in methanol (Nugroho and Morita, 2014; Mándi and Kurtán, 2019). All optimized conformers were checked for imaginary frequencies. The conformers were subjected to ECD calculations using TD-DFT B3LYP/def2svp as a basis set and an SCRF model in methanol in Gaussian16 software. The calculated ECD spectrum was generated in SpecVis1.71 software (Berlin, Germany) based on a Boltzmann-weighted average. Supplementary Figure S6 shows the results. The ECD calculations were performed on the HPC Baoab cluster at the University of Geneva.

### UHPLC-HRMS^2^ analysis

Analyses were performed with a Vanquish Horizon (Thermo Scientific, Germany) equiped with a binary pump H, a dual split sampler HT and a Diode Array detector FG coupled to an Orbitrap Exploris 120 mass spectrometer (Thermo Scientific, Germany) and a Corona Veo RS Charged Aerosol Detector (CAD, Thermo Scientific, Germany). The Orbitrap employes a heated electrospray ionization source (H-ESI) with the following parameters: spray voltage: +3.5 kV; ion transfer tube temperature: 320.00°C; vaporizer temperature: 320.00°C; S-lens RF: 45 (arb units); sheath gas flow rate: 35.00 (arb units); Sweep Gas (arb): 1, and auxiliary gas flow rate: 10.00 (arb. units).

The mass analyzer was calibrated using a mixture of caffeine, methionine−arginine−phenylalanine− alanine−acetate (MRFA), sodium dodecyl sulfate, sodium taurocholate, and Ultramark 1,621 in an acetonitrile/methanol/water solution containing 1% formic acid by direct injection. Control of the instruments was done using Thermo Scientific Xcalibur software v. 4.6.67.17. Full scans were acquired at a resolution of 30,000 fwhm (at *m/z* 200) and MS2 scans at 15,000 fwhm in the range of 100–1,000 *m/z*, with one microscan, time (ms): 200 m, an RF lens (%): 70; AGC target custom (Normalized AGC target (%): 300); maximum injection time (ms): 130; Microscans: 1; data type: profile; Use EASY-IC(TM): ON. The Dynamic exclusion mode: Custom; Exclude after n times: 1; Exclusion duration s): 5; Mass tolerance: ppm; low: 10, high: 10, Exclude isotopes: true. Apex detention: Desired Apex Window (%): 50. Isotope Exclusion: Assigned and unassigned with an exclusion window (*m/z*) for unassigned isotopes: 8. The Intensity threshold was set to 2.5E5. and a targeted mass exclusion list was used. The centroid data-dependent MS^2^ (dd-MS^2^) scan acquisition events were performed in discovery mode, triggered by Apex detection with a trigger detection (%) of 300 with a maximum injection time of 120 m, performing one microscan. The top three abundant precursors (charge states one and 2) within an isolation window of 1.2 m/z were considered for MS/MS analysis. For precursor fragmentation in the HCD mode, a normalized collision energy of 15, 30, 45% was used. Data was recorded in profile mode (Use EASY-IC(TM): ON).

The chromatographic separation was done on a Waters BEH C18 column (50 × 2.1 mm i. d., 1.7 *μ*m, Waters, Milford, MA) using a gradient as follows (time (min), %B): 0.5, 8.2; 7,99; 8,99; 8.10,8.2; 9.75, 8.2. The mobile phases were A) water with 0.1% formic acid and B) acetonitrile with 0.1% formic acid. The flow rate was set to 600 μL/min, the injection volume was 1 *μ*L, and the column was kept at 40 °C. The PDA detector was used from 210 to 400 nm with a resolution of 1.2 nm. The CAD was kept at 40°C, 5 bar N_2_, and power function one for a data collection rate of 20 Hz.

### Data conversion

All raw data files were converted using ThermoRawFileParser v.1.4.0.101 (https://github.com/compomics/ThermoRawFileParser) (Hulstaert et al., 2020).

### MZmine data preprocessing

The converted files were processed with MZmine3 (Schmid et al., 2023). For mass detection at the MS^1^ level, the noise level was set to 1.0 E^6^. For MS^2^ detection, the noise level was set to 0.00. The ADAP chromatogram builder parameters were set as follows: Minimum consecutive scans, 5; Minimum intensity for consecutive scans, 1.0 E^6^; Minimum absolute height, 1.0 E^6^, and *m/z* tolerance of 0.0020 or 10.0 ppm. The Local minimum feature resolver algorithm was used for chromatogram deconvolution with the following parameters: Chromatographic threshold, 80; Minimum search range RT/Mobility (absolute), 0.10; Minimum relative height, 1%; Minimum absolute height, 1.0 E^6^; Min ratio of peak top/edge, 1.0; peak duration range, 0.01–1.0 min; Minimum scans, 5. Isotopes were detected using the ^13^C isotope filter with an *m/z* tolerance of 0.0050 or 8.0 ppm, a Retention Time tolerance of 0.05 min (absolute), the maximum charge set at 2, and the representative isotope used was the most intense. Each file was filtered to remove duplicates using the Duplicate peak filter with an *m/z* tolerance of 0.005 or 10 ppm and an RT tolerance of 0.10 min. The Feature list row filter was used to filter with the following parameters: Minimum features in an isotope pattern, 2; Retention time, 0.50–7.00 min; Feature duration range: 0.1–1.0 min; and only the ions with an associated MS^2^ spectrum were kept. The resulting filtered list was subjected to Ion Identity Networking (Schmid et al., 2021) starting with the metaCorrelate module (RT tolerance, 0.10 min; minimum height, 1.0 E^5^; Intensity correlation threshold 1.0 E^5^ and the Correlation Grouping with the default parameters). Followed by the Ion identity networking (*m/z* tolerance, 8.0 ppm; check: one feature; Minimum height: 1.0 E^3^, Ion identity library [maximum charge, 2; maximum molecules/cluster, 2; Adducts ([M + H]^+^, [M + Na]^+^, [M + K]^+^, [M + NH_4_]^+^, [M+2H]^2+^), Modifications ([M-H_2_O], [M-2H_2_O], [M-CO_2_], [M + HFA], [M + ACN])], Annotation refinement (Delete small networks without major ion, yes; Delete networks without monomer, yes), Add ion identities networks (*m/z* tolerance, 8 ppm; Minimum height, 1.0 E^5^; Annotation refinement (Minimum size, 1; Delete small networks without major ion, yes; Delete small networks: Link threshold, 4; Delete networks without monomer, yes)) and Check all ion identities by MS/MS (*m/z* tolerance (MS^2^)), 10 ppm; min-height (in MS^2^), 1.0 E^3^; Check for multimers, yes; Check neutral losses (MS^1^ - > MS^2^), yes) modules. The resulting aligned peak list was exported as a. *mgf* file for further analysis.

### Spectral organization through molecular networking

A molecular network for HPE was constructed from the. *mgf* file exported from MZmine3, using the FBMN workflow on the GNPS platform (Wang et al., 2016; Nothias et al., 2020). The precursor ion mass tolerance was set to 0.02 Da with an MS^2^ fragment ion tolerance of 0.02 Da. A network was created where edges were filtered to have a cosine score above 0.7 and more than six matched peaks. The spectra in the network were then searched against GNPS’ spectral libraries. All matches between network and library spectra were required to have a score above 0.6, and at least three matched peaks. Job link: https://gnps.ucsd.edu/ProteoSAFe/status.jsp?task=c9e133b094404c0ab373c991b8924fb0.

### Taxonomically informed metabolite annotation

The.*mgf* file exported from MZmine3 was also annotated by spectral matching against an *in silico* database to obtain putative annotations (Allard et al., 2016). The resulting annotations were subjected to taxonomically informed metabolite scoring (https://taxonomicallyinformedannotation.github.io/tima-r/, *v 2.8.2*) and re-ranking (Rutz et al., 2019) from the chemotaxonomic information available on LOTUS (Rutz et al., 2022). The *in silico* database used for this process includes the combined records of the Dictionary of Natural Products (DNP, *v*30.2) and the LOTUS Initiative outputs.

### SIRIUS metabolite annotation

The SIRIUS. *mgf* file exported from MZmine3 (using the SIRIUS export module) that contains MS^1^ and MS^2^ information was processed with SIRIUS (v5.6.3) (Dührkop et al., 2019). The parameters were set as follows: Possible ionizations: [M + H]^+^, [M + NH_4_]^+^, [M-H_2_O + H]^+^, [M + K]^+^, [M + Na]^+^, [M-4H_2_O + H]^+^; Instrument profile: Orbitrap; Mass accuracy: 5 ppm for MS^1^ and 7 ppm for MS^2^, the Database for molecular formulas and structures: BIO, Maximum *m/z* to compute: 1,000. ZODIAC was used to improve molecular formula prediction using a threshold filter of 0.99 (Ludwig et al., 2020). Metabolite structure prediction was made with CSI: FingerID (Dührkop et al., 2015) and the significance was computed with COSMIC (Hoffmann et al., 2022). The chemical class prediction was made with CANOPUS (Dührkop et al., 2021) using the NPClassifier chemical taxonomy (Kim et al., 2021).

### Wnt activity assessing assay

#### Cell lines and culture conditions

The BT-20, HCC1395 and HEK293 cell lines were cultured and utilized in experimental conditions in Dulbecco’s Modified Medium (Thermo Fisher Scientific) supplemented with 10% Fetal Calf Serum and 1% penicillin-streptomycin at 37°C and 5% CO_2_.

#### Luciferase-based assay of the Wnt-dependent transcriptional activity

Purified Wnt3a was obtained from mouse L-cells stably transfected with Wnt3a, as previously described (Willert et al., 2003), with our own modifications (Xu et al., 2020). The 3 cell lines, stably transfected with the M50 Super 8×TopFlash plasmid, were seeded at a density of 6,000 cells/well in white tissue-culture-treated 384-well plates in 20 *µ*L/well maintenance medium. The cells were also transfected with the pCMV-RL plasmid to allow for constitutive expression of Renilla luciferase, using XtremeGENE nine reagent according to the manufacturer’s protocol. After 24 h post-transfection, the medium was replaced with 2-fold indicated concentrations of compounds in 10 *µ*L/well maintenance medium. Following a 1-h preincubation, Wnt3a was added to a final concentration of 500 ng/mL in an additional 10 *µ*L/well volume. After a further 24 h of incubation, the medium was removed and measurements were taken using a Tecan Infinite M200 PRO plate reader equipped with a two-channel dispensing unit by injecting sequentially 15 *µ*L of each of the buffer solutions for activity measurements of firefly and Renilla luciferase, as described previously (Boudou et al., 2022). The resulting dose-response data for this and the MTT assay were fitted using GraphPad Prism nine software (*v*9.4.0, Boston, United States) to obtain IC_50_ values. Since the assay is designed to not use positive control compounds, an extract or compound is considered ‘*toxic*’ if the IC50 value against Renilla luciferase is less than 1.7-fold of estimated TopFlash one, indicating that the decrease observed in TopFlash response is affected by a strong toxic effect.

## Conclusion

The findings of this study demonstrate the potential of combining *Inventa*’s structural novelty scores with bioactivity results for guiding the discovery of structurally novel bioactive NPs in collections of NEs. Through the evaluation of Wnt-regulation activity results and *Inventa*’s scores, a collection of 1,600 NEs was narrowed down to four active NEs with a high potential of containing structural novel NPs.

*Inventa*’s multifaceted approach to evaluating structural richness and dissimilarities among extracts proves instrumental in this process. By assessing individual features within each extract and comparing the overall spectral space*, Inventa* effectively identifies extracts with potentially unknown specialized metabolisms. Through the integration of data from these two levels and the incorporation of literature reports for the taxon, *Inventa* highlights extracts with high novelty potential. The priority score, derived from its four components provides a comprehensive evaluation of the NEs potential of containing novel NPs. While *Inventa*’s novelty scores may not directly correlate with observed bioactivity, they play a crucial role in prioritizing NEs and reducing selection prior to *in-depth* phytochemical study. This approach mitigates the risk of prioritizing known NPs and underscores the importance of employing comprehensive bioinformatics approaches in sample selection. Thus, *Inventa* empowers researchers to identify NEs harboring structurally novel NPs with potential therapeutic applications.

The subsequent phytochemical investigation of *H. punctata* leaves led to the isolation of ten novel bicyclo[3.3.1]non-3-ene-2,9-diones and three known prenylated flavones. Some of the newly isolated compounds exhibited appreciable IC_50_ values and showed no apparent cytotoxicity in three different cell lines, indicating their potential as Wnt inhibitory compounds. This work illustrates the utility of *Inventa* in assisting the efficient selection of active NEs from large sample collections for the identification of novel and bioactive NPs.

## Data Availability

The mzML and raw UHPLC-HMRMS2 data for all the H. punctata de novo extract, can be accessed through MassIVE with the accession number MSV000092572. To access the molecular network follow the hyperlink: https://gnps.ucsd.edu/ProteoSAFe/status.jsp?task=c9e133b094404c0ab373c991b8924fb0 (PI). The ISDB and Sirius annotations (CSI:FingerID and CANOPUS), and the Cytoscape files for the molecular network in positive ionization pode are available here: https://massive.ucsd.edu/ProteoSAFe/dataset_files.jsp?task=ee8c8d92afd744c99ba0b6e1a53dfa0c#%7B%22table_sort_history%22%3A%22main.collection_asc%22%7D. The standard workflow used for processing and generating the Feature-Based Molecular Networking can be found in the GNPS documentation. The workflow for ISDB annotation and taxonomical re-weighting can be found here: https://taxonomicallyinformedannotation.github.io/tima-r/index.html. The script for cleaning and consolidating the annotations from GNPS can be found here: https://github.com/luigiquiros/inventa. The raw data files for the NMR and ECD (calculated and experimental) analysis are available at the following link: https://doi.org/10.26037/yareta:tnynbxbghrayrjqhnrzax4ijgi.
