# Supplementary material for: Integration of Wnt-inhibitory activity and structural novelty scoring results to uncover novel bioactive natural products: new Bicyclo[3.3.1]non-3-ene-2,9-diones from the leaves of Hymenocardia punctata
Source: Front Chem. 2024 Apr 4;12:1371982. doi: 10.3389/fchem.2024.1371982 (PMC11024435; doi:10.3389/fchem.2024.1371982)
Supplement: Supplementary file 1 [file DataSheet1.PDF]

SUPPLEMENTARY INFORMATION

Integration of Wnt-inhibitory Activity and Structural Novelty Scoring Results to Uncover Novel Bioactive Natural Products: New Bicyclo[3.3.1]non-3-ene-2,9-diones from the Leaves of *Hymenocardia punctata*.

Luis-Manuel Quiros-Guerrero[1,2,*], Laurence Marcourt[1,2], Nathareen Chaiwangrach[3], Alexey Koval[4,5], Vladimir L. Katanaev[4,5], Emerson Ferreira-Queiroz[1,2], Bruno David[4], Antonio Grondin[6], Jean-Luc Wolfender[1,2,*].

[1] Institute of Pharmaceutical Sciences of Western Switzerland, University of Geneva, CMU, 1211 Geneva, Switzerland.
[2] School of Pharmaceutical Sciences, University of Geneva, CMU, 1211 Geneva, Switzerland.
[3] Centre of Excellence in Cannabis Research, Department of Pharmaceutical Chemistry and Pharmacognosy, Faculty of Pharmaceutical Sciences, Naresuan University, Phitsanulok 65000, Thailand.
[4] Department of Cell Physiology and Metabolism, Translational Research Centre in Oncohaematology, Faculty of Medicine, Geneva, Switzerland.
[5] Institute of Life Sciences and Biomedicine, Far Eastern Federal University, Vladivostok, Russia.
[6] Green Mission Department, Herbal Products Laboratory, Pierre Fabre Research Institute, Toulouse, France.
corresponding author(s): Luis Quiros-Guerrero (luis.guerrero@unige.ch), Jean-Luc Wolfender (jean-luc.wolfender@unige.ch)

Supplementary Table SI. Selected annotation in positive ionization mode for the extract of *H. punctata* (HPE).

Row ID	*m/z*	Retention time (min)	chemical class	SpectrumID	InchiKey (2D structures)	Name
152	409.2009	4.50	Flavanones	CCMSLIB00005744844	CEBSROOTTDEPKN-UHFFFAOYSA-N	Bolusanthol C
194	435.2171	4.46	Flavanones	Sirius	IDUZXXVWQZBMFV-UHFFFAOYSA-N	8-(2,4-dihydroxyphenyl)-5,7-dihydroxy-2,2-dimethyl-10-(3-methylbut-2-enyl)-7,8-dihydropyrano[3,2-g]chromen-6-one
207	439.1759	3.28	Flavanones	Sirius	YGIMNZIIYOGVNP-UHFFFAOYSA-N	Lupinisol C
30	255.0653	3.20	Flavones	CCMSLIB00000006048	RTIXKCRFFJGDFG-UHFFFAOYSA-N	Diadzein
57	315.0865	3.35	Flavones	CCMSLIB00010105201	ROCUOVBWAWAQFD-UHFFFAOYSA-N	3,7-Dihydroxy-3',4'-dimethoxyflavone
67	337.1073	4.12	Flavones	CCMSLIB00006421975	YABIJLLNNFURIJ-UHFFFAOYSA-N	Psoralidin
86	355.1178	3.00	Flavones	Sirius	GLRTXWXOVXNDGS-UHFFFAOYSA-N	8-(2,6-dihydroxyphenyl)-5-hydroxy-2,2-dimethyl-7,8-dihydropyrano[3,2-g]chromen-6-one
121	391.1906	5.69	Flavones	CCMSLIB00005722626	YBJJPNXZVZRDIU-UHFFFAOYSA-N	Erysubin F
227	449.1077	1.32	Flavones	CCMSLIB00010112331	ODBRNZZJSYPIDI-VJXVFPJBSA-N	Isoorientin
34	269.0809	4.10	Flavones	CCMSLIB00000205268	PTXIWVJSAQPKEY-UHFFFAOYSA-N	7-Hydroxy-3'-methoxyflavone
65	329.1021	3.78	Flavones	CCMSLIB00010105025	GTTYINPAUAIFMI-UHFFFAOYSA-N	3-Hydroxy-6,3',4'-trimethoxyflavone
77	343.1178	3.28	Flavones	CCMSLIB00006422351	URSUMOWUGDXZHU-UHFFFAOYSA-N	Tetramethylscutellarein
136	403.1543	5.84	Isoflavanones	Sirius	ACNNVOPYPNMOSB-UHFFFAOYSA-N	Ulexone B
140	405.1698	4.84	Isoflavanones	CCMSLIB00004719514	DCTLJGWMHPGCOS-UHFFFAOYSA-N	Osajin
162	421.1649	4.57	Isoflavanones	CCMSLIB00004719155	GHCZYXUOYFOXIP-UHFFFAOYSA-N	pomiferin
163	421.1654	3.78	Isoflavanones	Sirius	ZSSVYEUXLNVQMH-UHFFFAOYSA-N	3-(2,4-dihydroxyphenyl)-5-hydroxy-8,8-dimethyl-6-(3-methylbut-2-enyl)pyrano[2,3-h]chromen-4-one
204	439.1757	3.70	Isoflavanones	Sirius	IDUZXXVWQZBMFV-UHFFFAOYSA-N	8-(2,4-dihydroxyphenyl)-5,7-dihydroxy-2,2-dimethyl-10-(3-methylbut-2-enyl)-7,8-dihydropyrano[3,2-g]chromen-6-one
83	353.1384	4.47	Isoflavones	CCMSLIB00005739462	JQNSUDIGIIGIOL-UHFFFAOYSA-N	Gancaonin A
87	355.1178	3.55	Isoflavones	CCMSLIB00005748211	KCUZCRLRQVRBBV-UHFFFAOYSA-N	Licoisoflavone A
147	407.1853	4.54	Isoflavones	CCMSLIB00005722080	FXJPTJQFJYNFKC-UHFFFAOYSA-N	3,8-diprenyl-4'5,7-trihydroxyisoflavone
124	395.1495	4.12	Pyrano coumarin	Sirius	WZBWCXHRCQICHJ-UHFFFAOYSA-N	3-[2,4-dihydroxy-3-(3-methylbut-2-enyl)phenyl]-5-hydroxy-6,8,8-trimethyl-2,3-dihydropyrano[2,3-h]chromen-4-one

Supplementary Table SII. Wnt-activity results of the pooled fractions of the Microfractionation of the ethyl acetate extract of *Hymenocardia punctata* leaves.

Code	IC50, µg/ml	Comment
**HPE-A**	21.49	
**HPE-B**	~43	extrapolated
**HPE-C**	NA	inactive upon retesting
**HPE-E**	~100	extrapolated
**HPE-F**	~43	extrapolated
**HPE-G**	NA	inactive upon retesting
**HPE-H**	~29	extrapolated
**HPE-6**	20	
**HPE-9**	~28	extrapolated
**HPE-10**	23.82	
**HPE-11**	7.30	

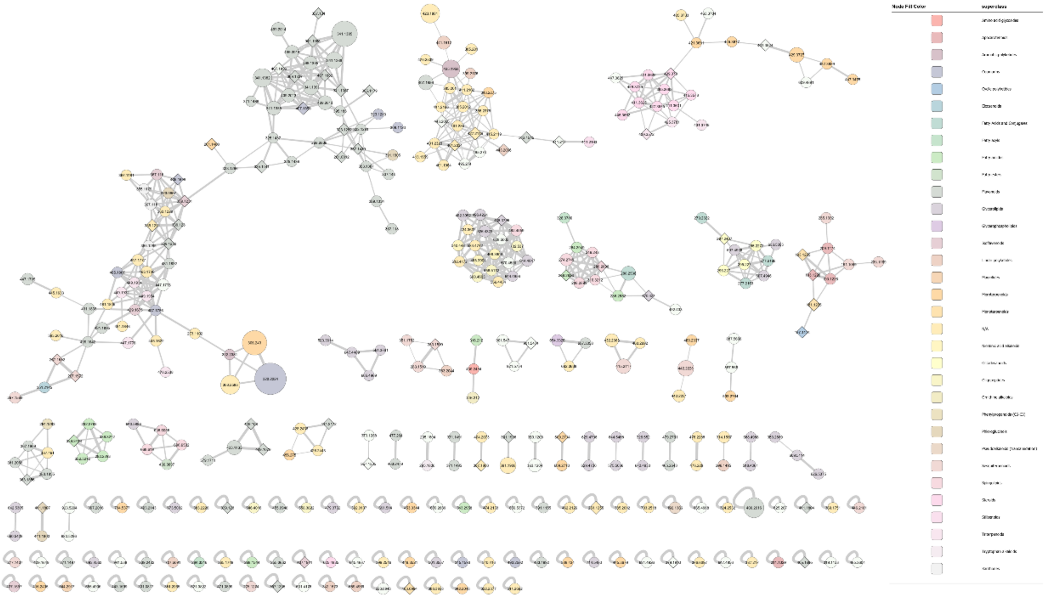

Supplementary Figure S1. Ion Identity-FBMN in positive mode for the original *Hymenocardia punctata* leaves extract (PDF version here). Node size is proportional to the chromatographic peak intensity. Dashed lines correspond to MS[1] correlations based on Ion Identity MN[61]. Color code corresponds to the ‘class’ given by CANOPUS. The annotated nodes are rhomboid-shaped while the unannotated nodes are round. The full list of color codes for the chemical classes can be found in here).

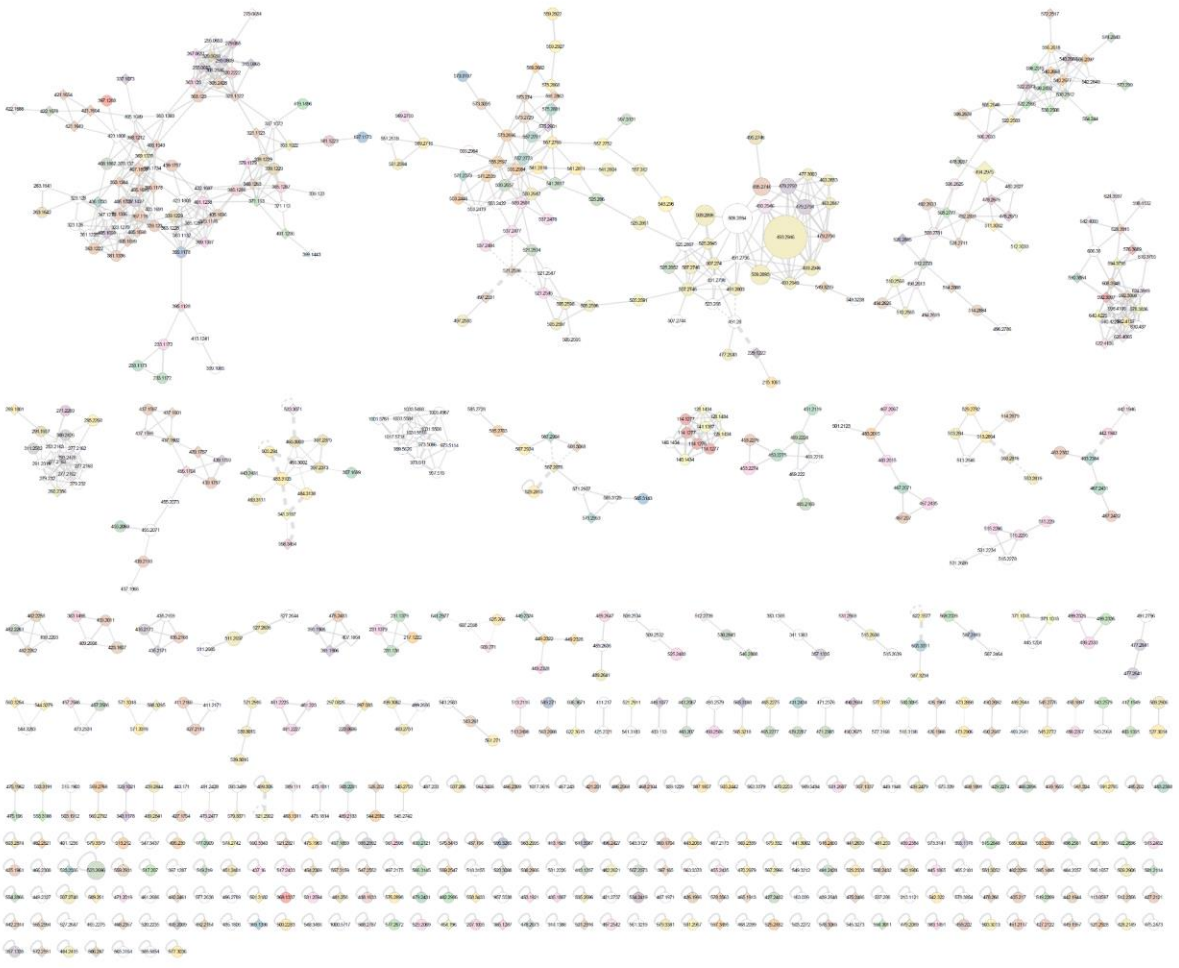

Supplementary Figure S2. II-FBMN in positive mode for *de novo Hymenocardia punctata* leaves ethyl acetate extract (HPE, PDF version here). The enlarged section corresponds to the second largest cluster which contains the most intense features in the chromatogram. Node size is proportional to the chromatographic peak intensity. Dashed edges correspond to MS^1 correlations based on II-FBMN. Color code corresponds to the 'class' given by CANOPUS. The annotated nodes are rhomboid-shaped while the unannotated nodes are round. The full list of color codes for the chemical classes can be found here.

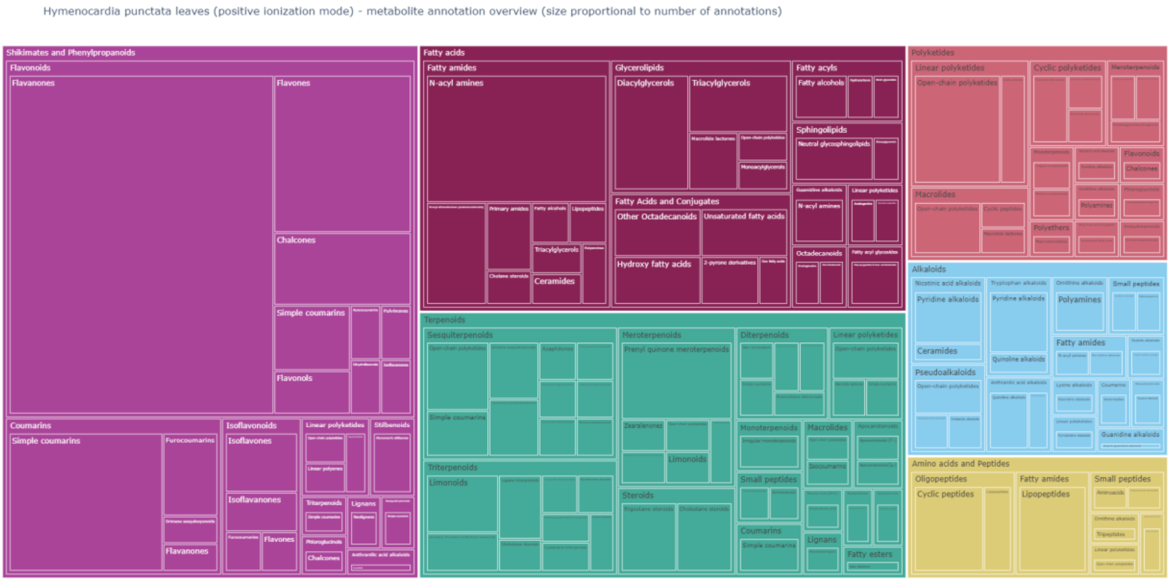

Supplementary Figure S3. Treemap overview of the chemical class metabolite annotation in positive mode for the extract of *H. punctata*. (interactive treemap here). The Sunburst plot version can be found here.

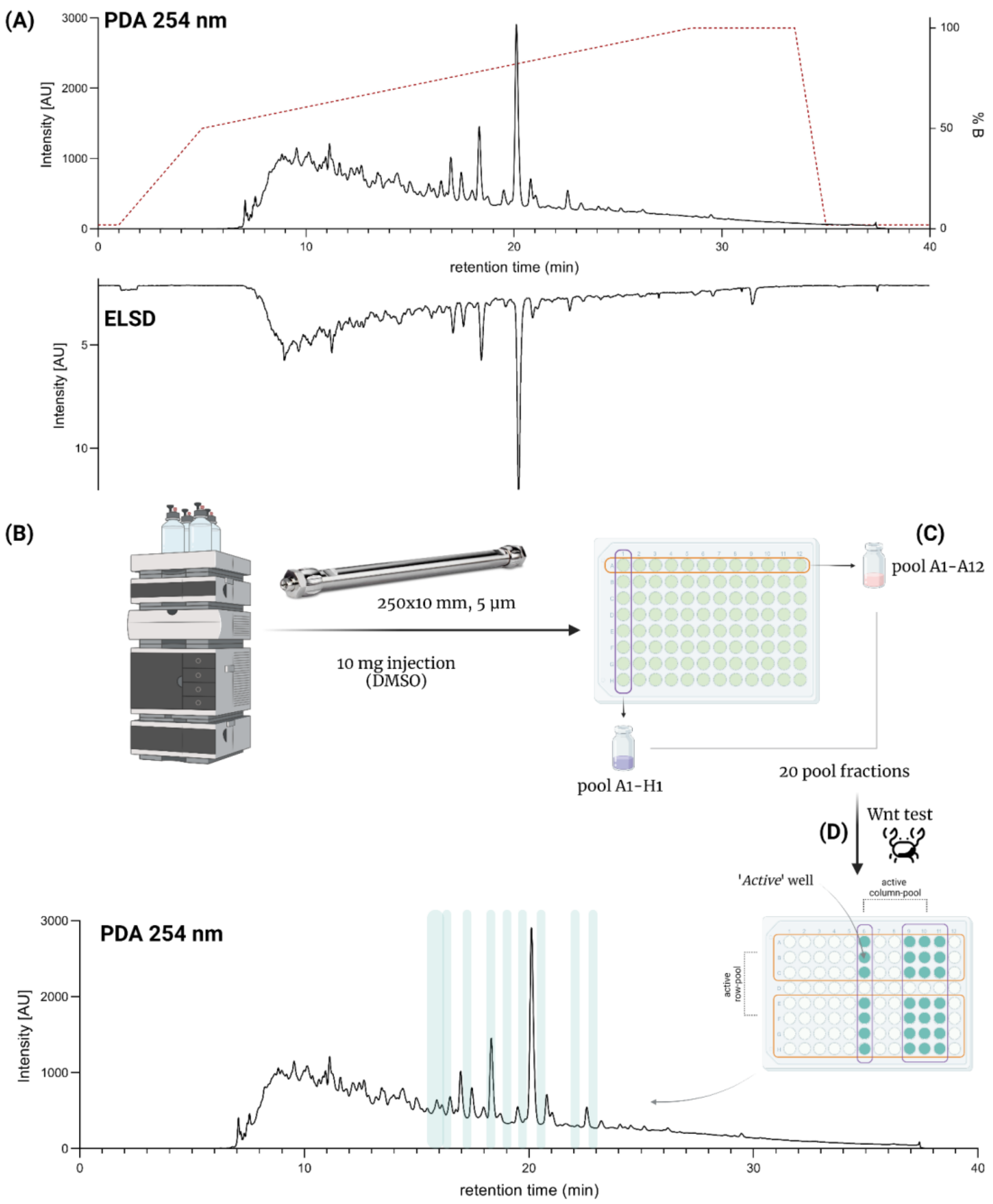

Supplementary Figure S4. General overview of the micro fractionation strategy used for locating the bioactivity in the extract of *Hymenocardia punctata*. A) First the chromatographic profile was optimized in a window of 40 min in an HPLC column (254x4.6 mm, 5µm); the positive trace shows the optimized chromatogram at 254 nm and the inverted trace corresponds to the Evaporative Light Scattering semiquantitative detector (ELSD). B) The chromatographic conditions were scaled up using a gradient transfer [22,23] to a semi-preparative column (254x10 mm, 5µm); a total of 10 mg were separated using this method, and a total of 96 fractions were collected (2 mL 96-well plate). C) The fractions were then pooled row-wise (pool

from A1 to A12, from B1 to B12, etc.) and column-wise (pool from A1 to H1, from B1 to B12, etc.), dried, and dissolved to a 5 mg/mL concentration in DMSO for bioactivity assessment. Only 20 fractions need to be tested by pooling the fractions instead of the 96 wells. By crossing the results of the active samples, intersections of the active row-wise pools and columns-wise pools led to the direct identification of the wells containing possible active compounds (See Supplementary Table S2). The collection time of the active wells was used to highlight the chromatogram regions where isolation efforts should be concentrated. D) The bioactivity results of the pooled fractions were used to highlight the region of the chromatogram where the bioactive compounds are possibly present (the green region in the 254 nm chromatographic trace).

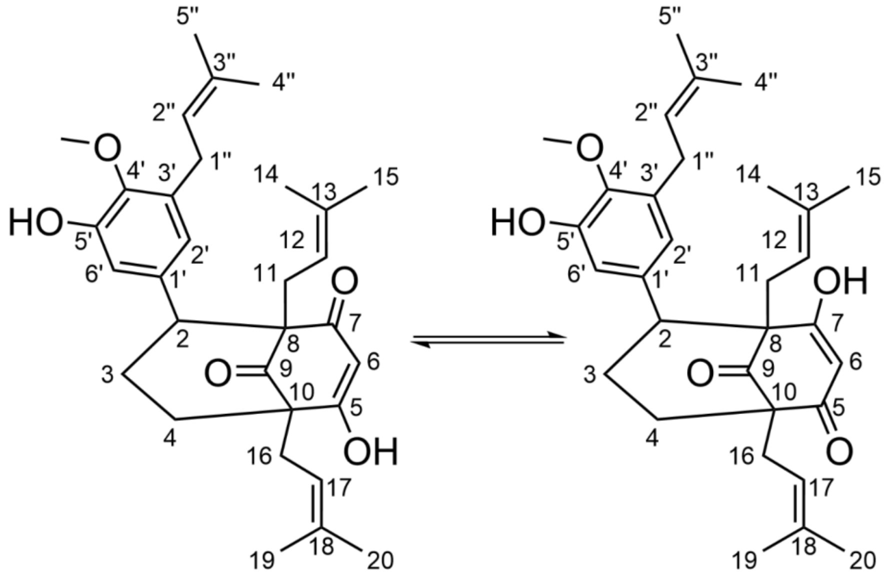

Supplementary Figure S5. Tautomeric equilibrium representation

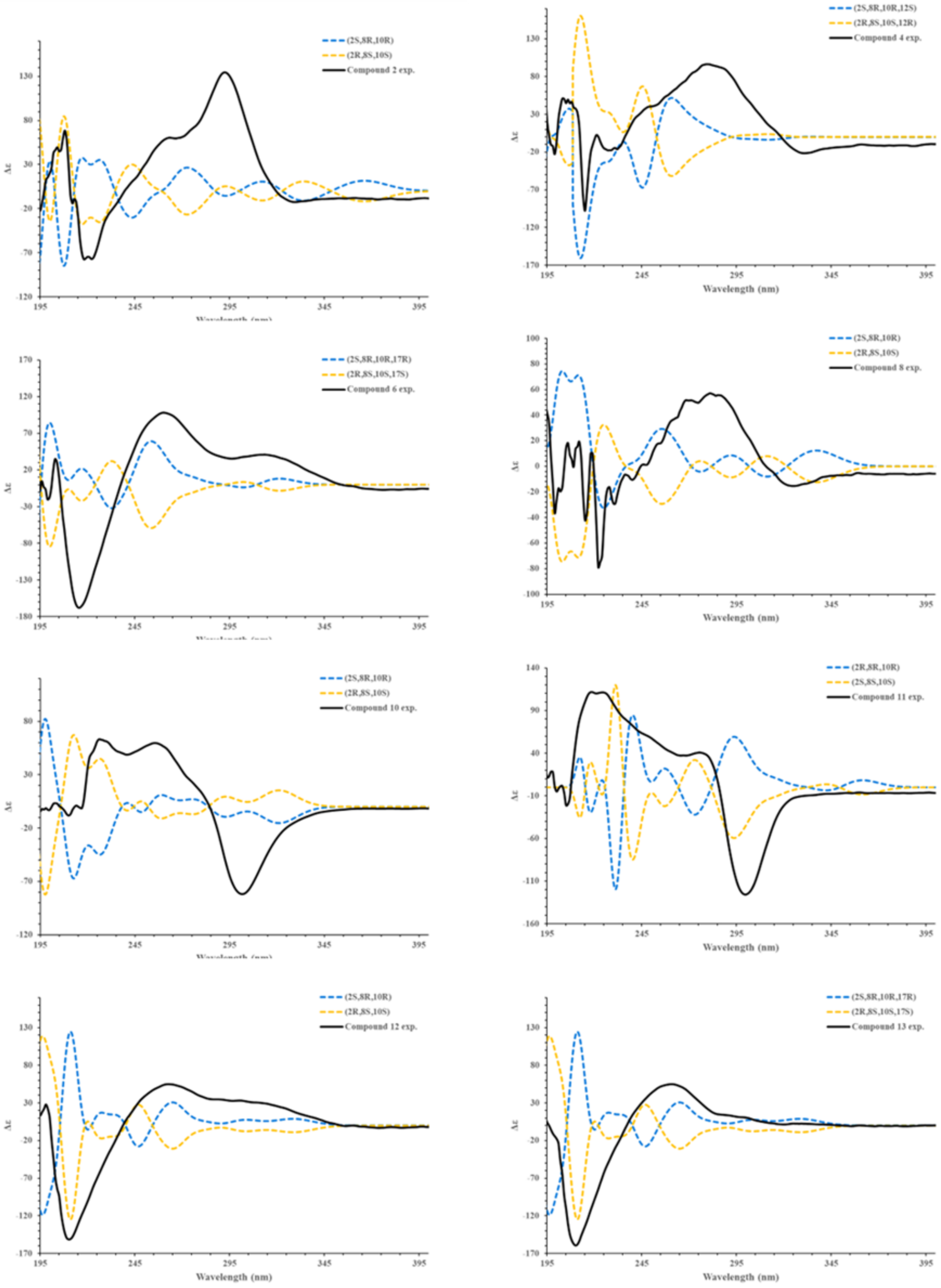

Supplementary Figure S6. Experimental and B3LYP/def2svp//B3LYP/l-31G(d,p) calculate Electronic Circular Dichroism spectra for compounds 1-13 in methanol

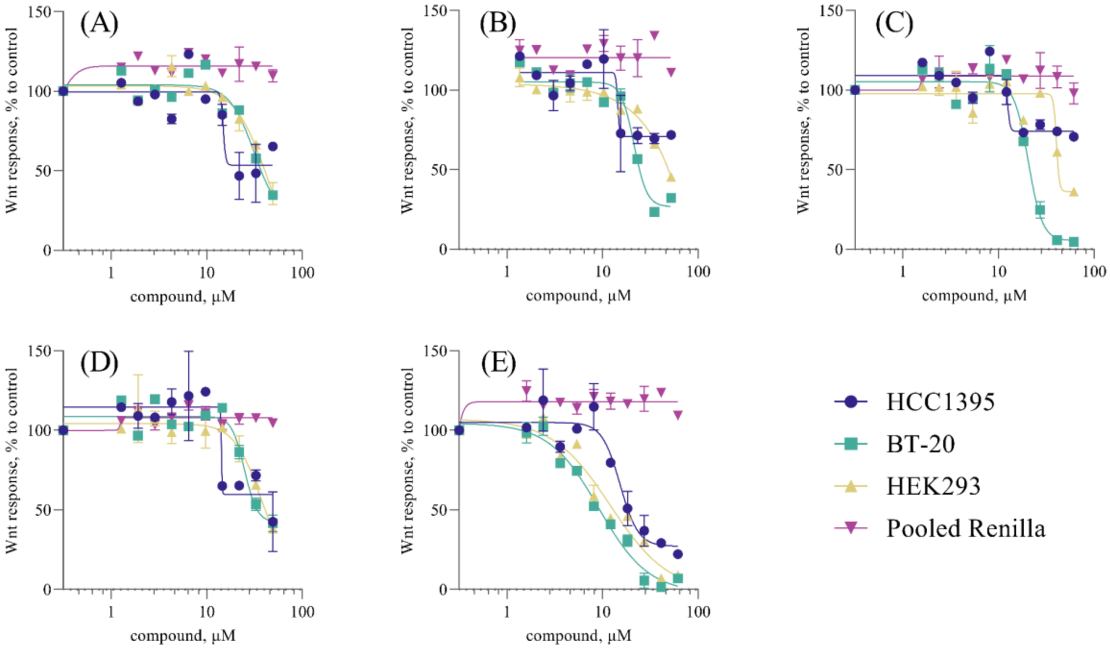

Supplementary Figure S7. Wnt response to Wnt3a stimulation, in % of control, plots for A) compound **1**, B) compound **2**, C) compound **3**, D) compound **4**, and E) compound **7**. The plots show the dose-response effect for each cell line (HCC1395, BT-20, HEK293) and the response of Renilla luciferase (cytotoxicity).

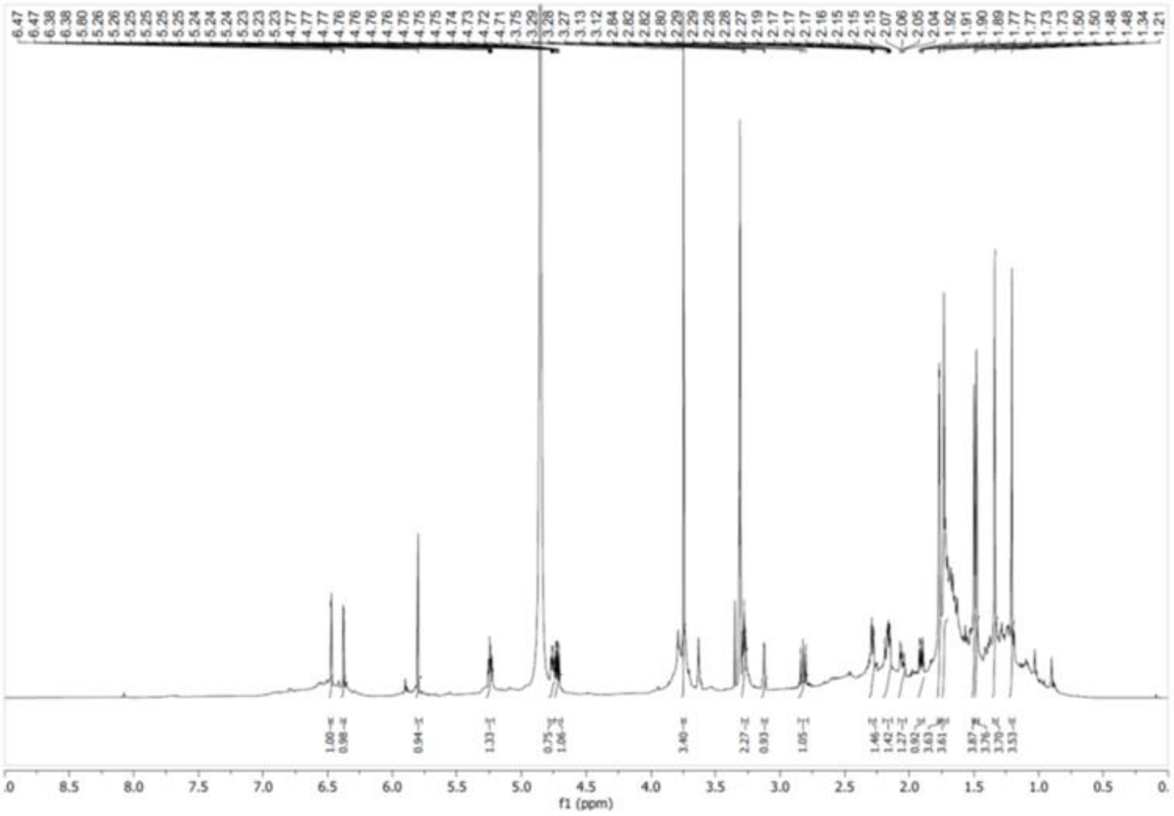

Supplementary Figure S8. ^{1}H NMR spectrum of compound **1** in CD_3OD at 600 MHz.

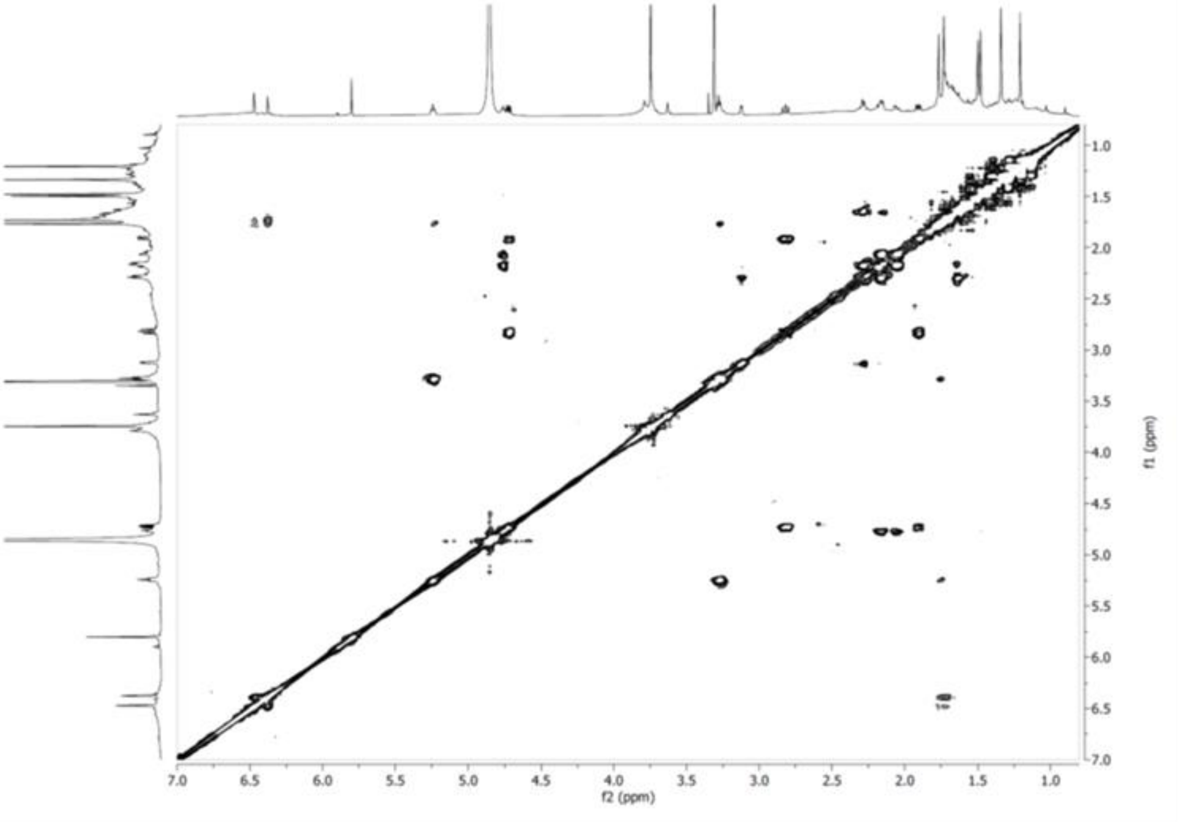

Supplementary Figure S9. COSY NMR spectrum of compound **1** in CD_3OD.

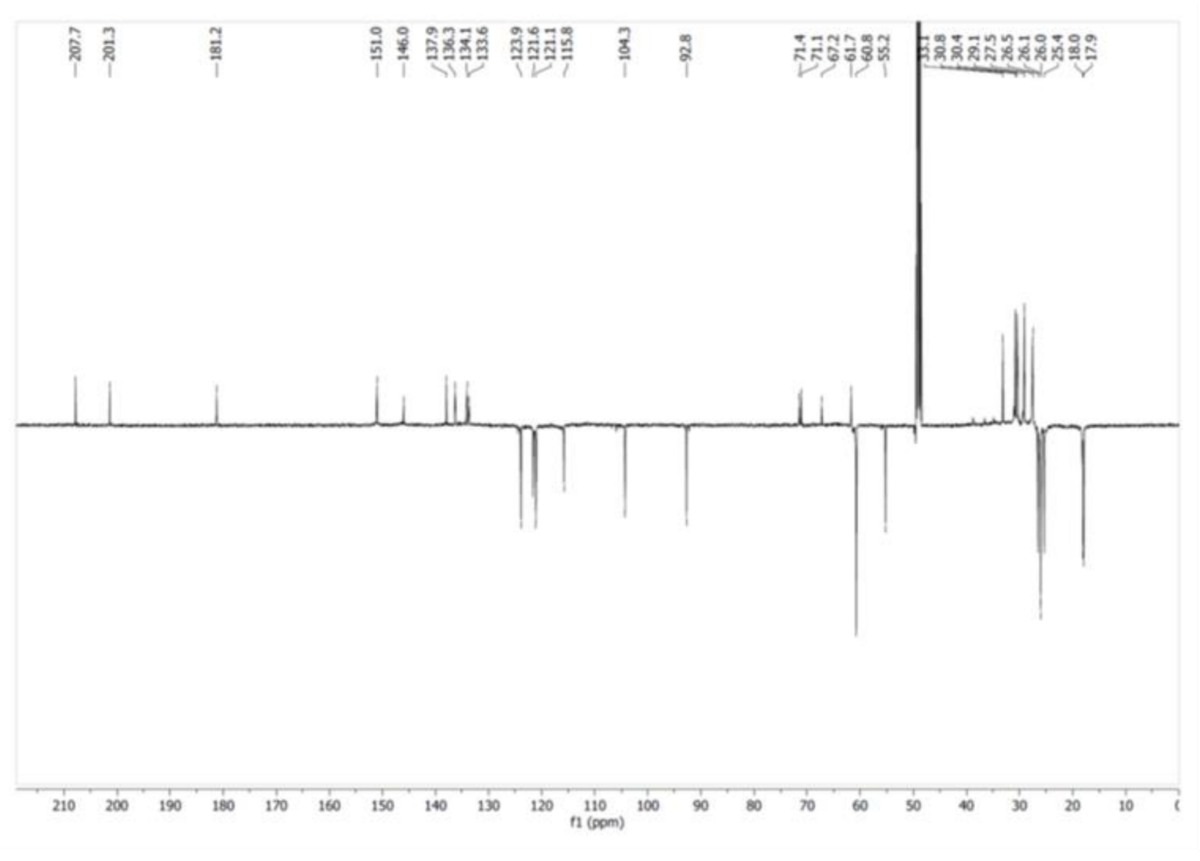

Supplementary Figure S10. ^{13}C-DEPTQ NMR spectrum of compound **1** in CD_3OD at 151 MHz

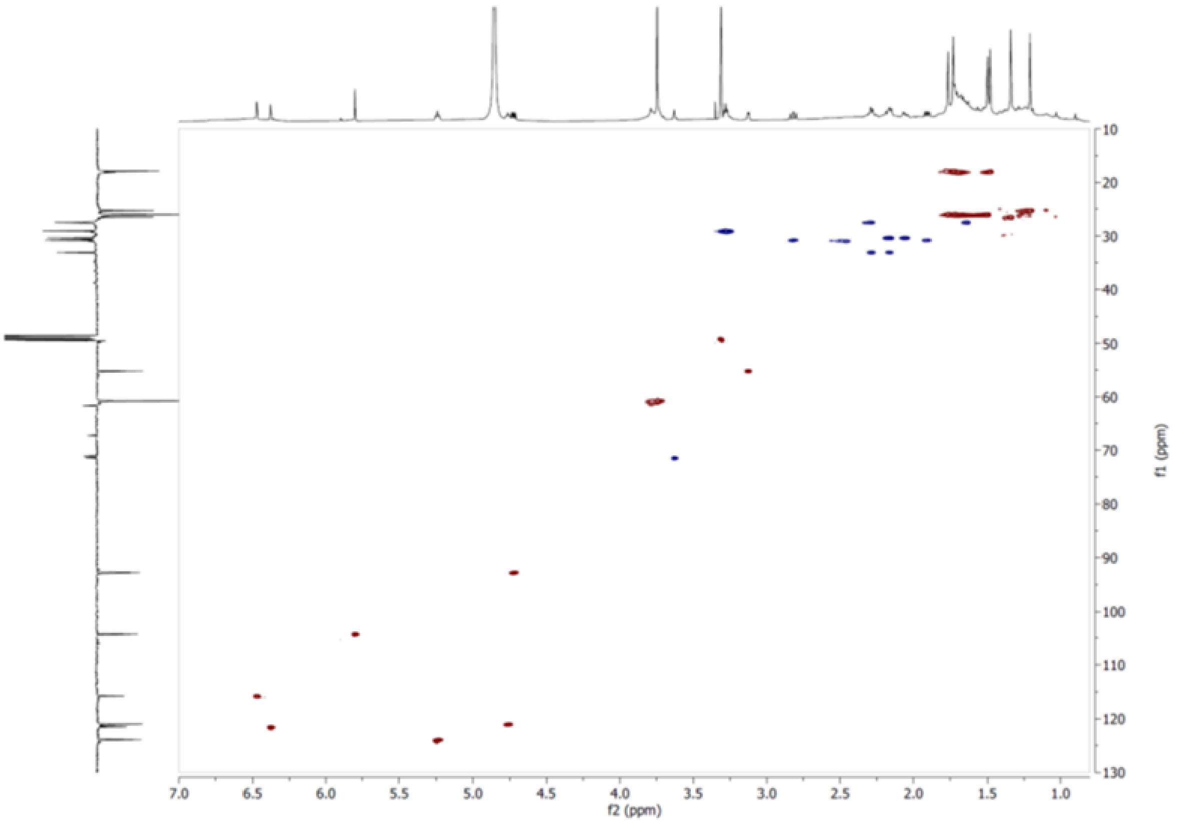

Supplementary Figure S11. Edited HSQC NMR spectrum of compound **1** in CD_3OD.

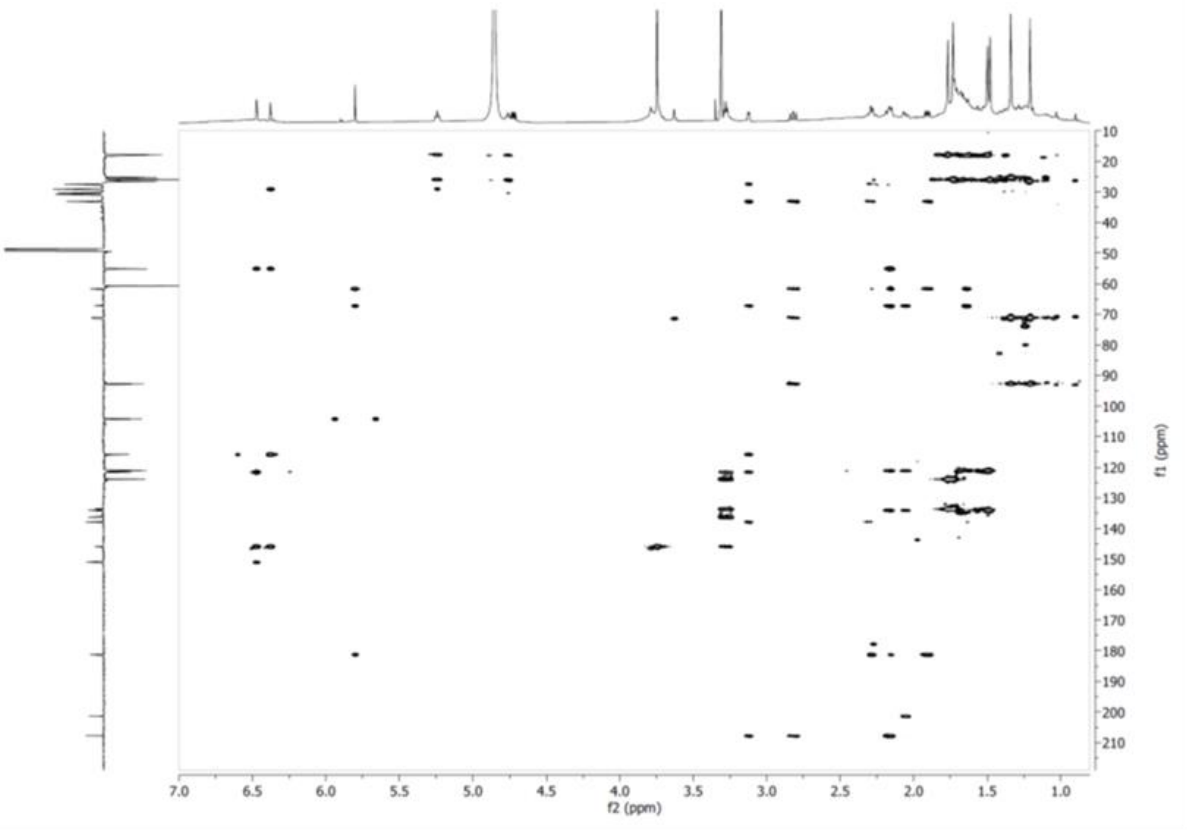

Supplementary Figure S12. HMBC NMR spectrum of compound **1** in CD_3OD.

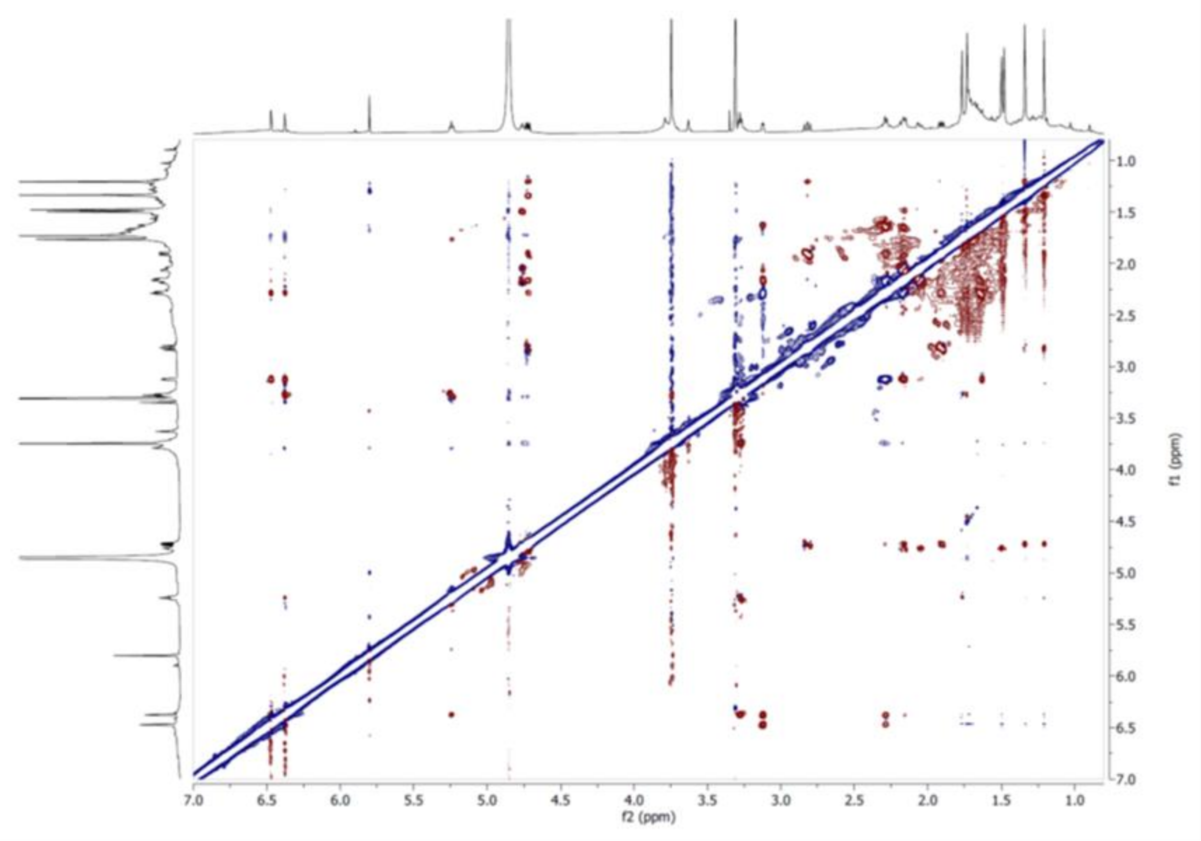

Supplementary Figure S13. ROESY NMR spectrum of compound **1** in CD_3OD.

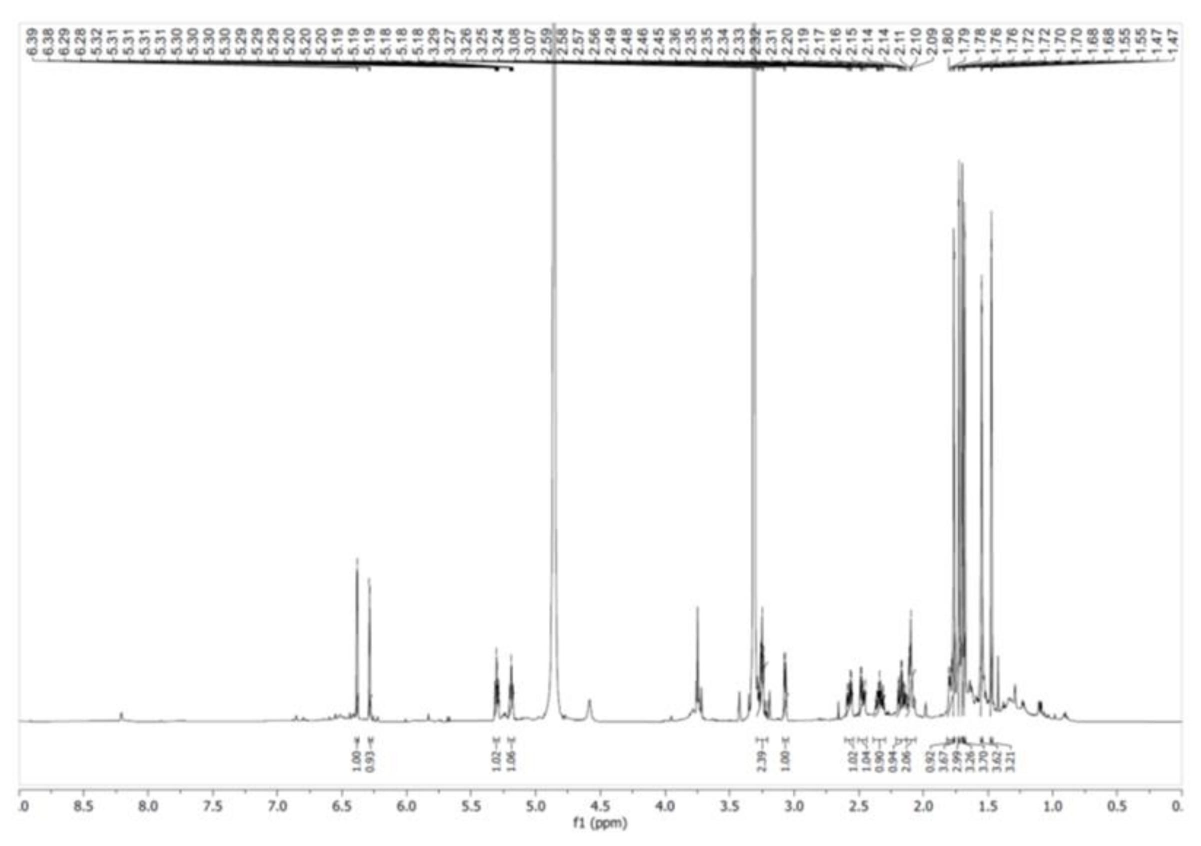

Supplementary Figure S14. ^1H NMR spectrum of compound **2** in CD_3OD at 600 MHz.

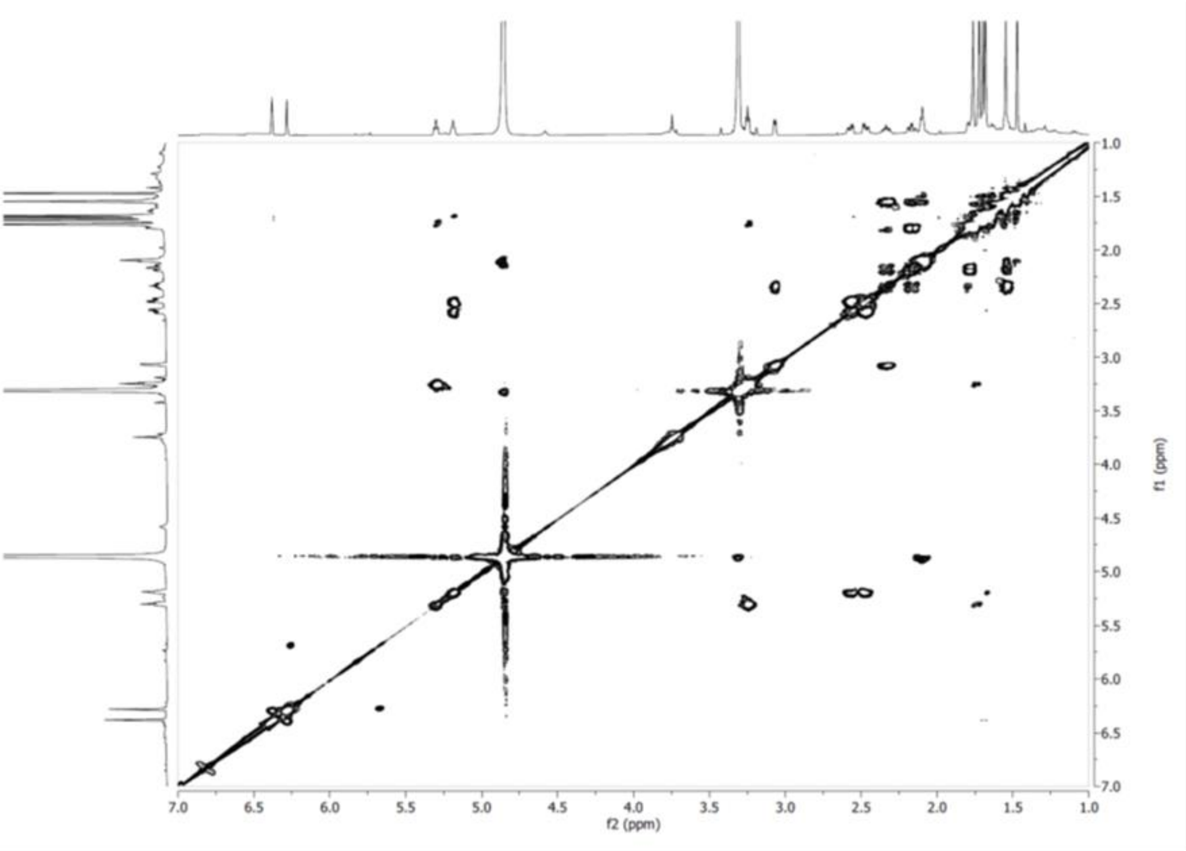

Supplementary Figure S15. COSY NMR spectrum of compound **2** in CD_3OD.

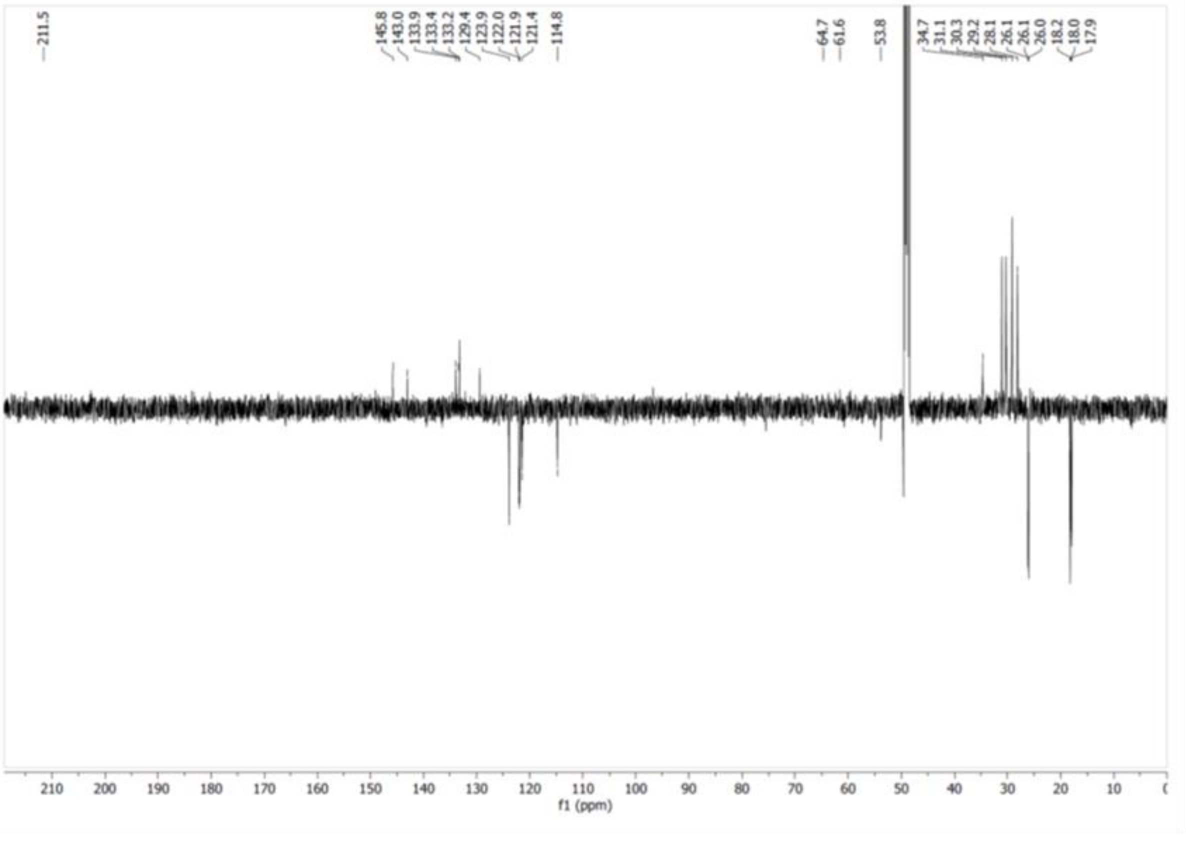

Supplementary Figure S16. ^{13}C-DEPTQ NMR spectrum of compound **2** in CD_3OD at 151 MHz.

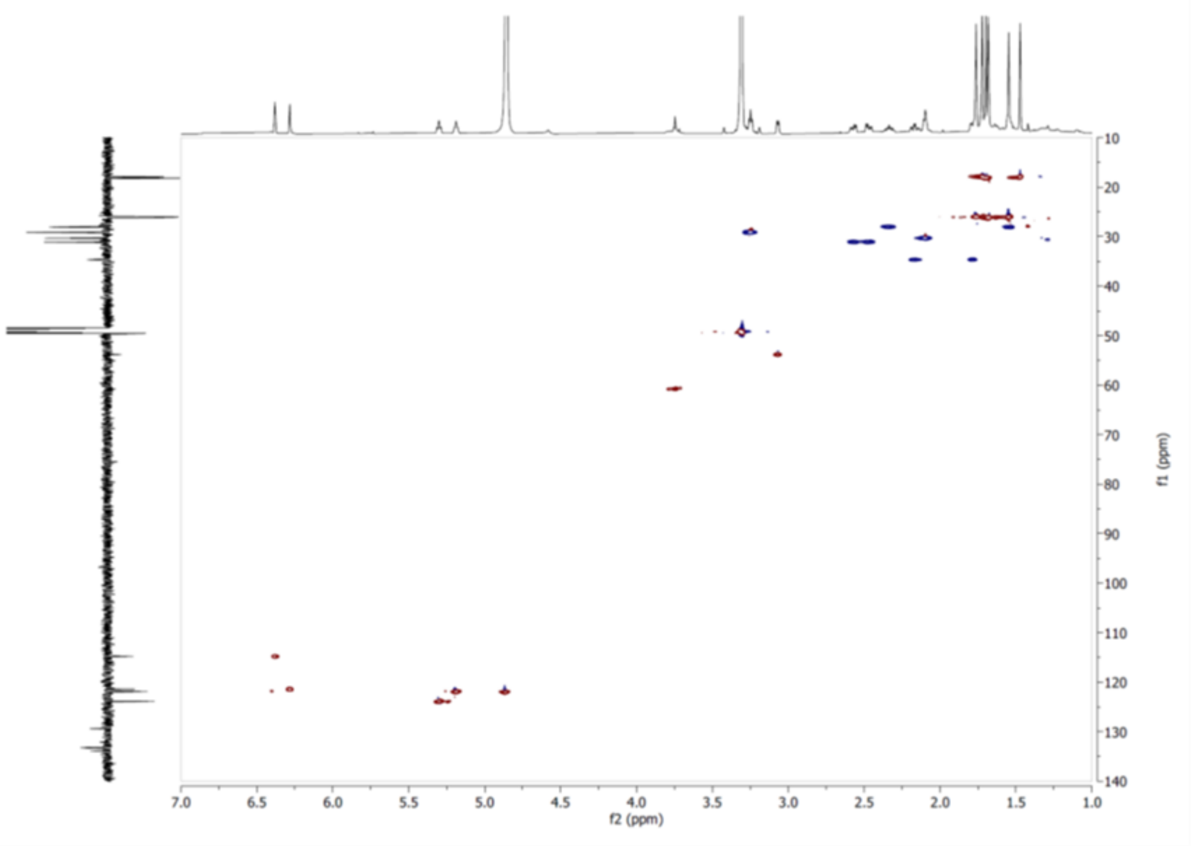

Supplementary Figure S17. Edited HSQC NMR spectrum of compound **2** in CD_3OD.

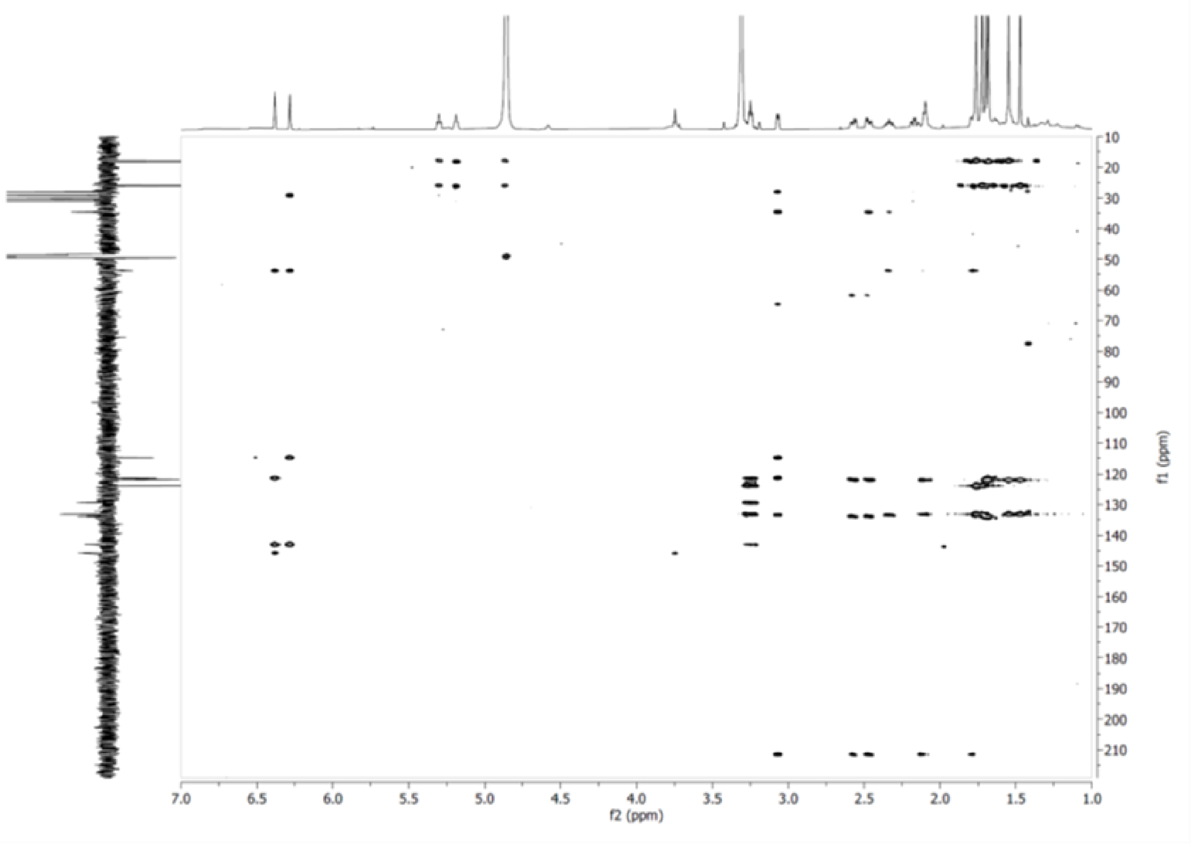

Supplementary Figure S18. HMBC NMR spectrum of compound **2** in CD_3OD.

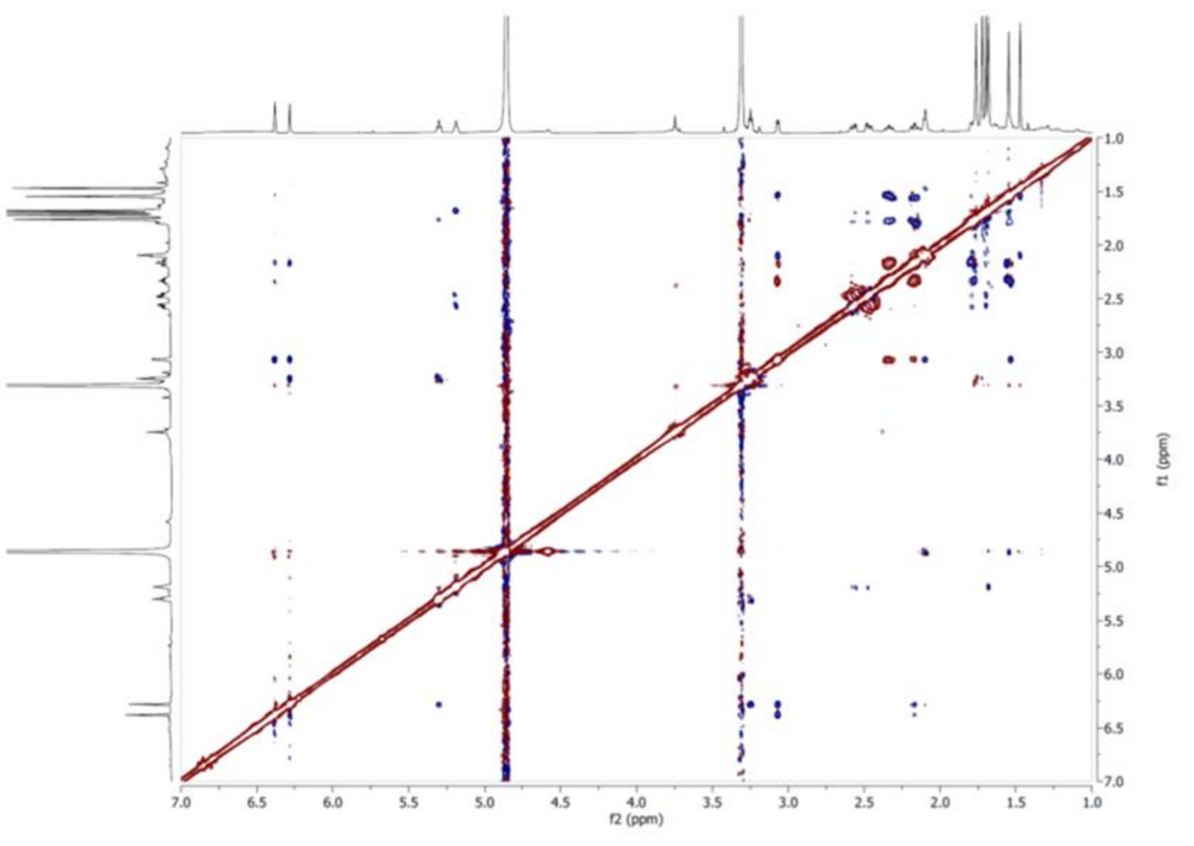

Supplementary Figure S19. ROESY NMR spectrum of compound **2** in CD_3OD.

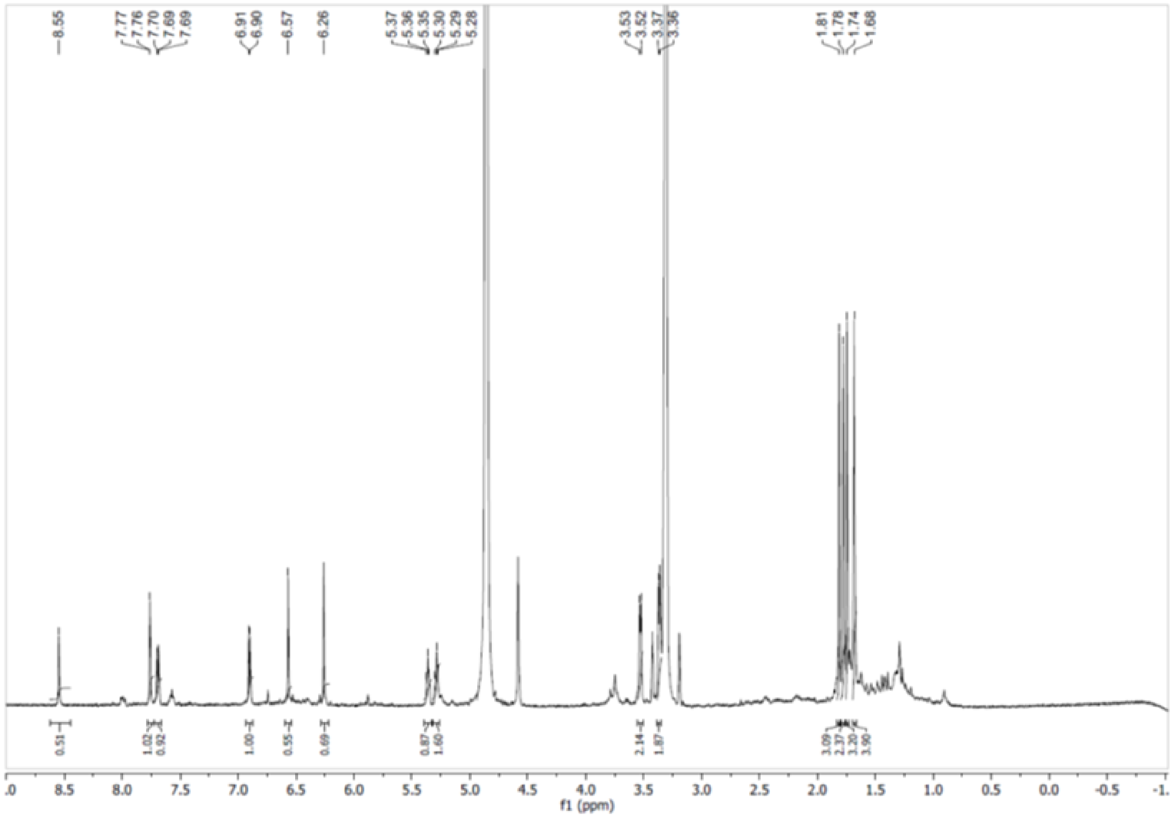

Supplementary Figure S20. ^1H NMR spectrum of compound **3** in CD_3OD at 600 MHz.

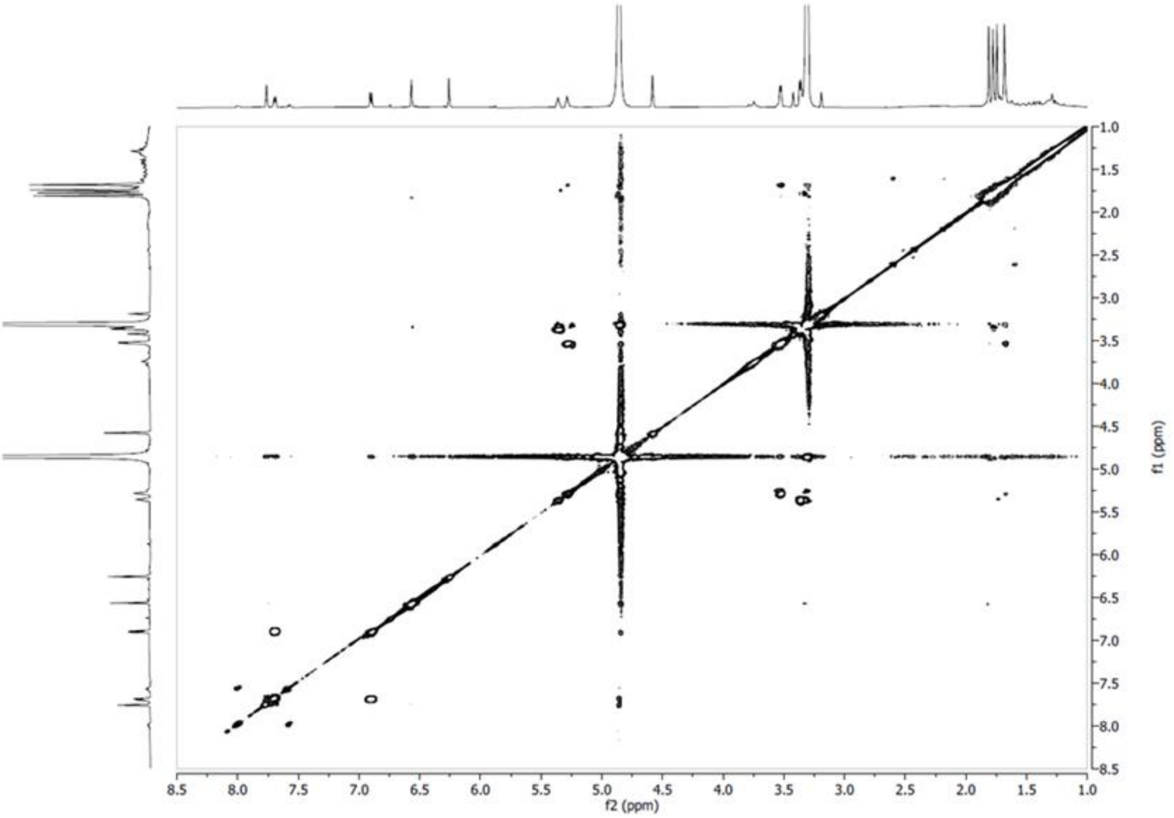

Supplementary Figure S21. COSY NMR spectrum of compound **3** in CD_3OD.

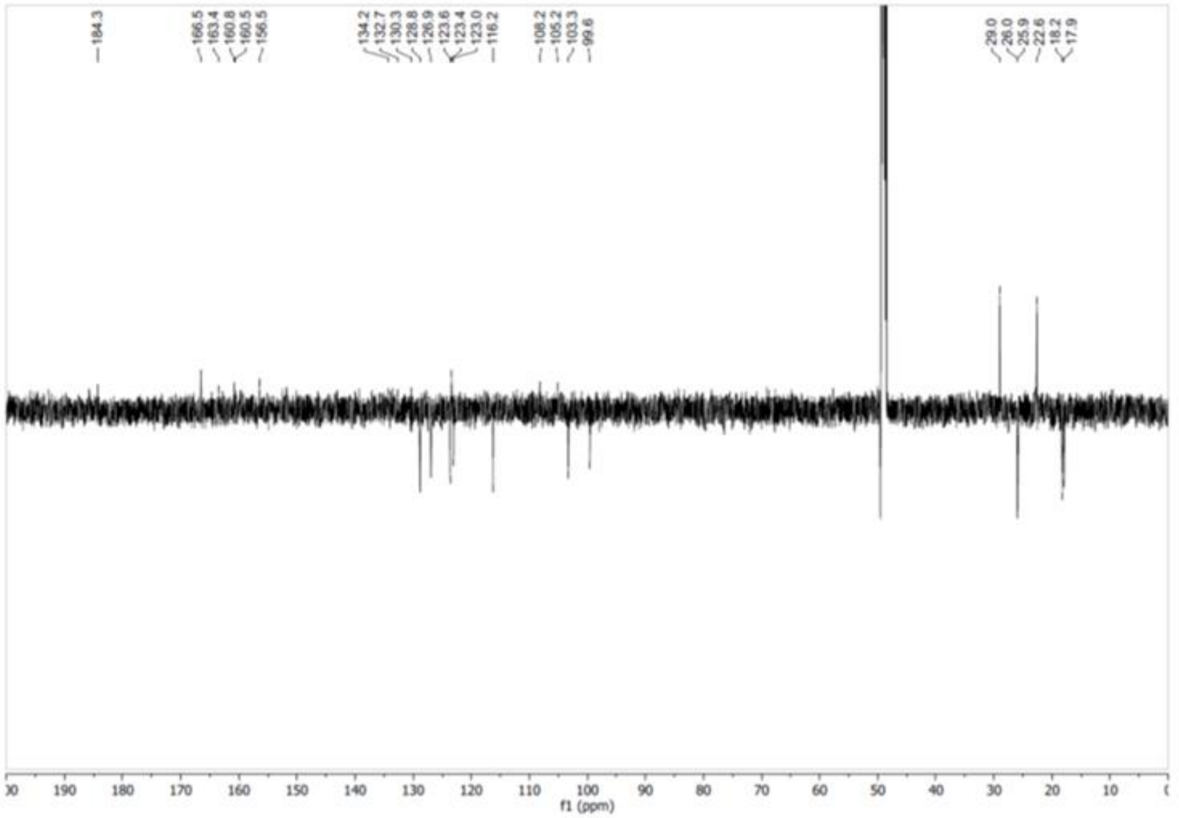

Supplementary Figure S22. ^{13}C-DEPTQ NMR spectrum of compound **3** in CD_3OD at 151 MHz.

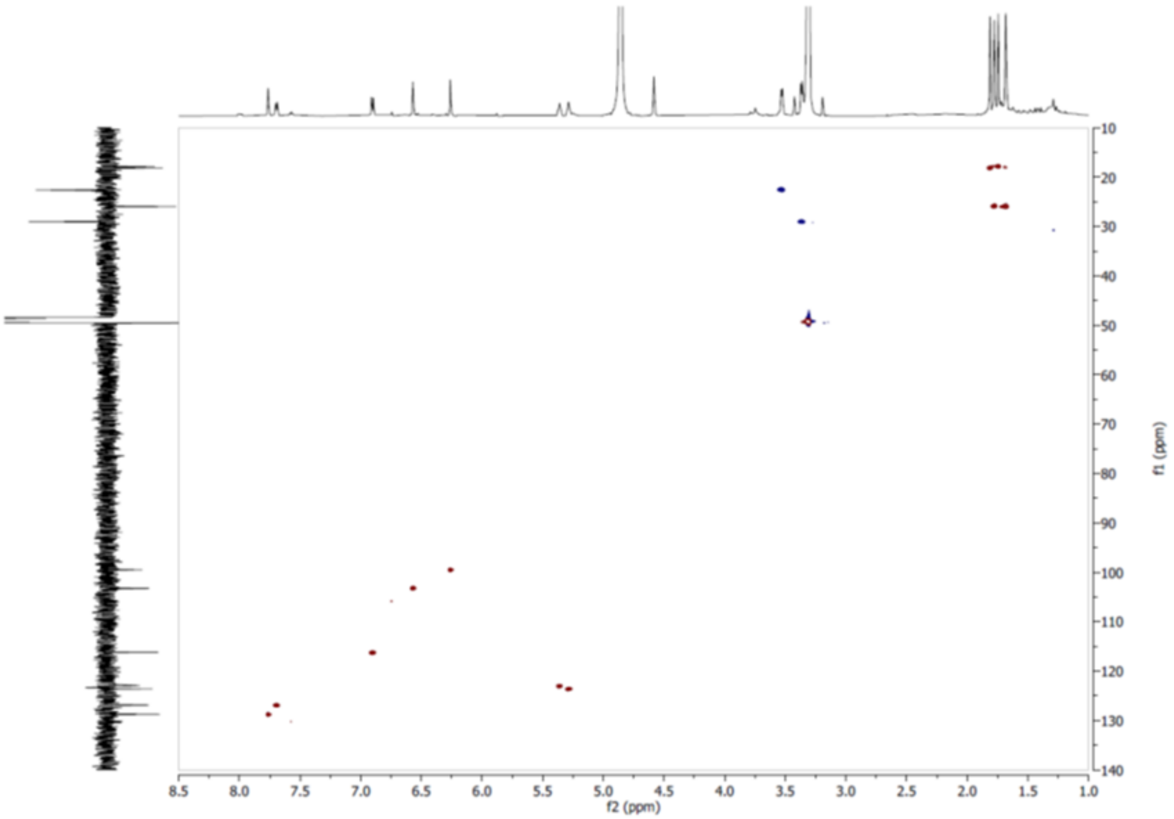

Supplementary Figure S23. Edited HSQC NMR spectrum of compound **3** in CD_3OD.

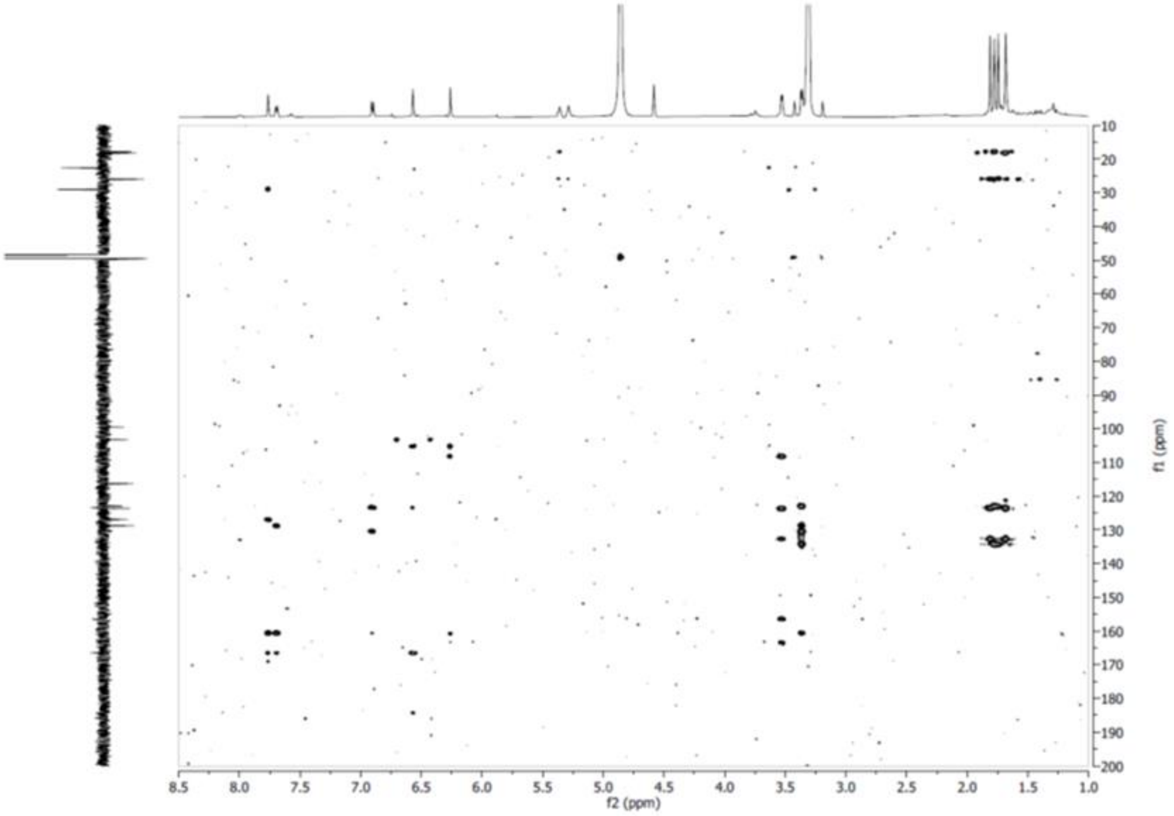

Supplementary Figure S24. HMBC NMR spectrum of compound **3** in CD_3OD.

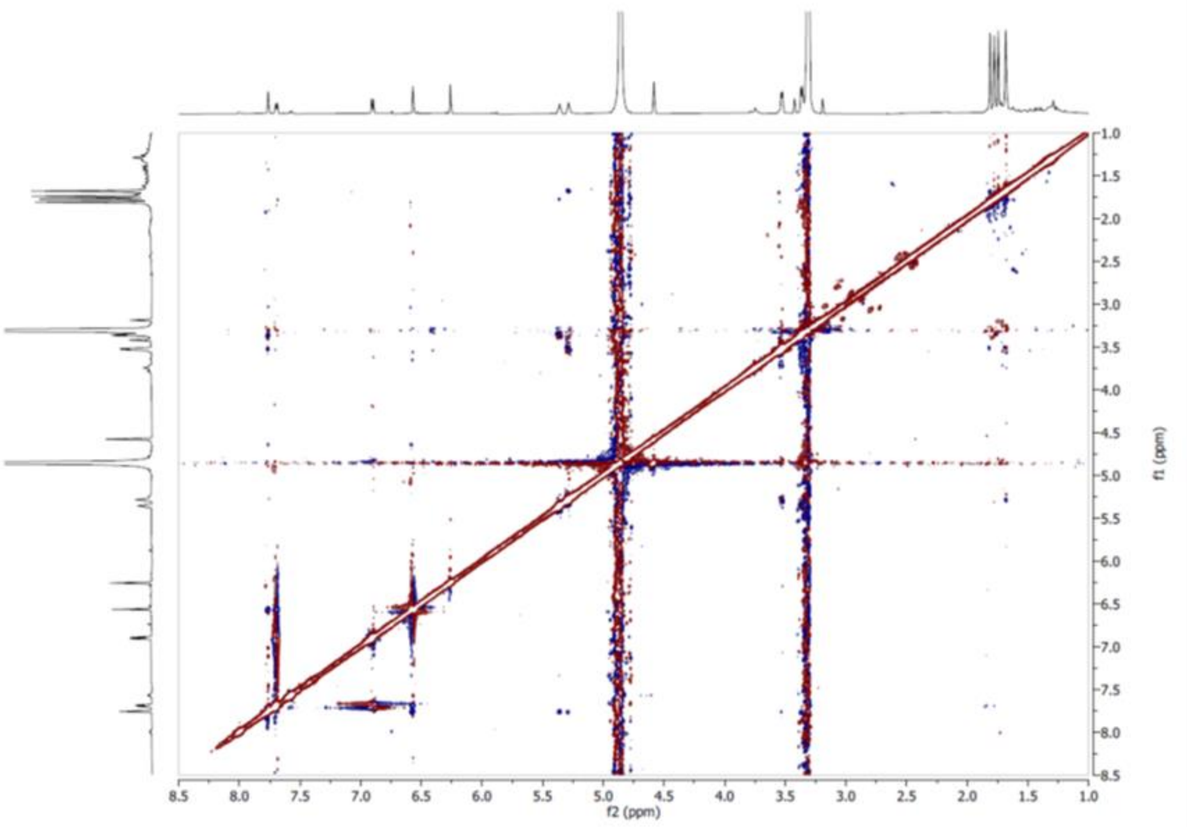

Supplementary Figure S25. ROESY NMR spectrum of compound **3** in CD_3OD.

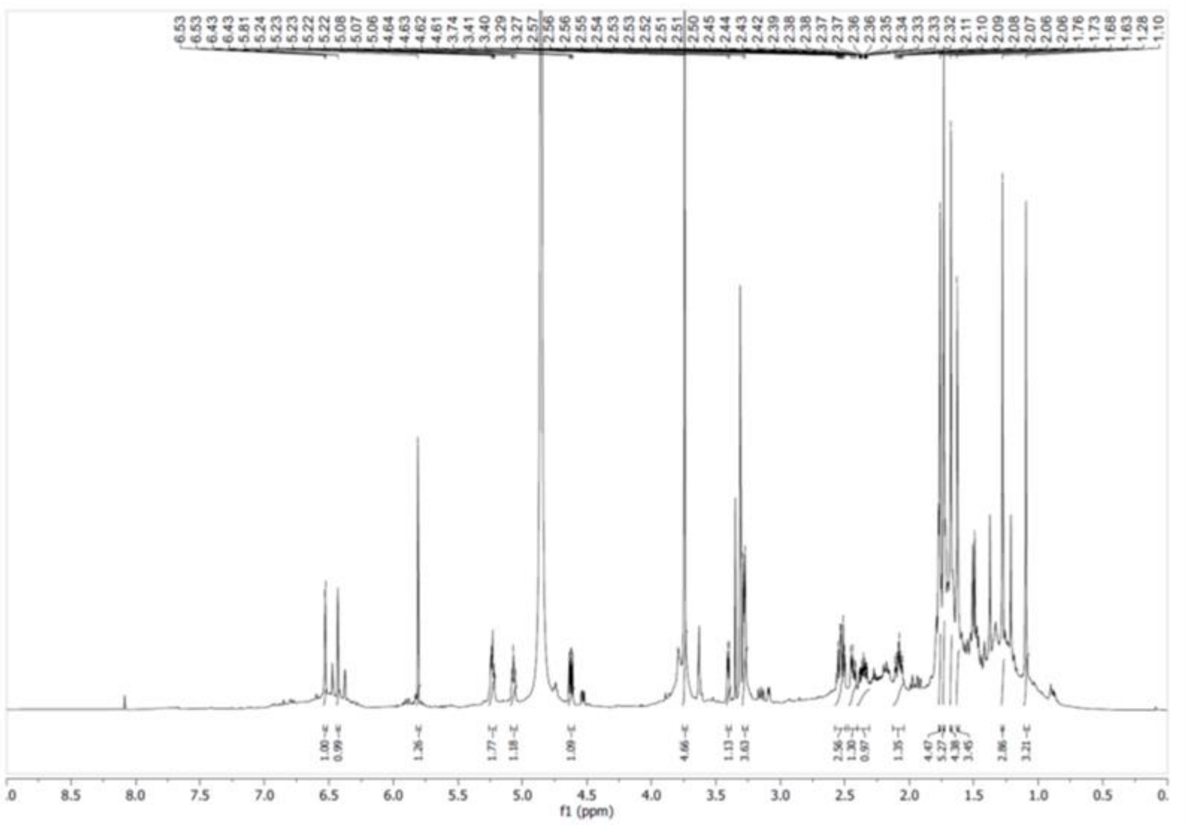

Supplementary Figure S26. ^1H NMR spectrum of compound **4** in CD_3OD at 600 MHz.

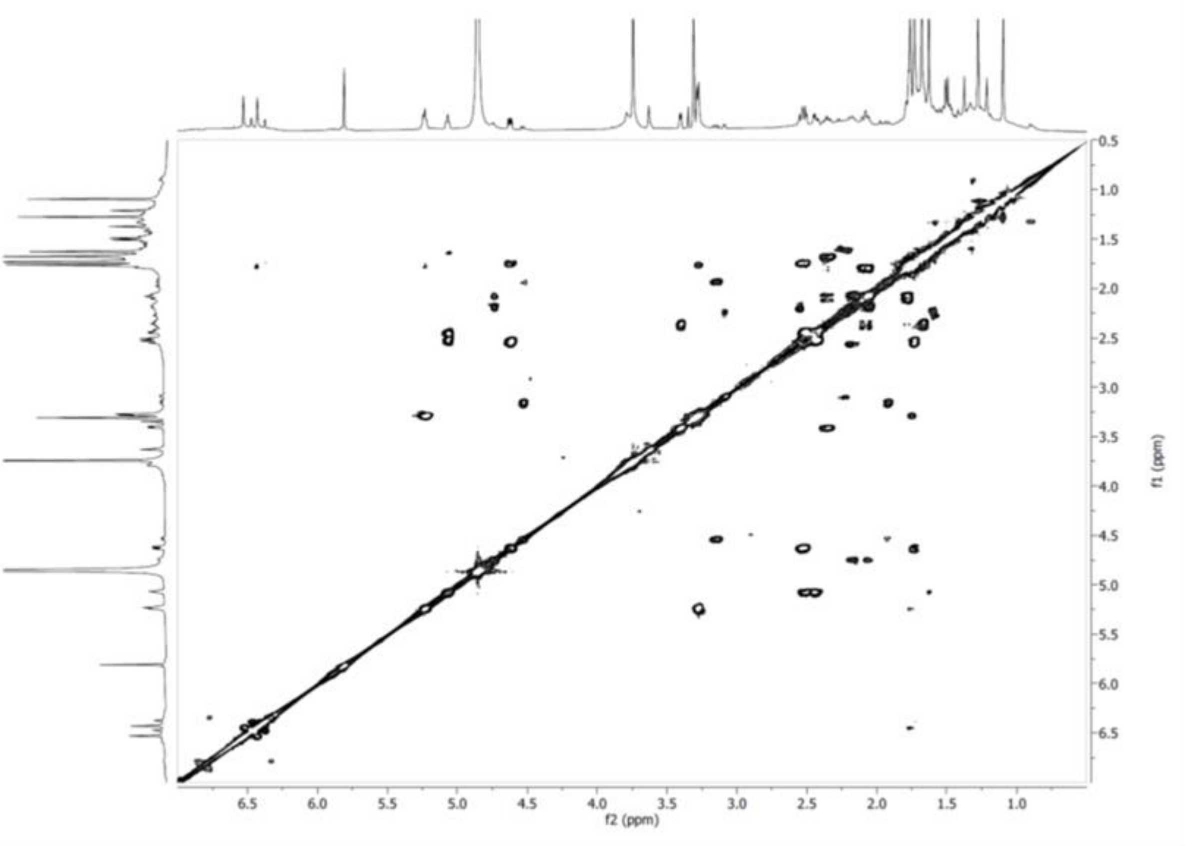

Supplementary Figure S27. COSY NMR spectrum of compound **4** in CD_3OD.

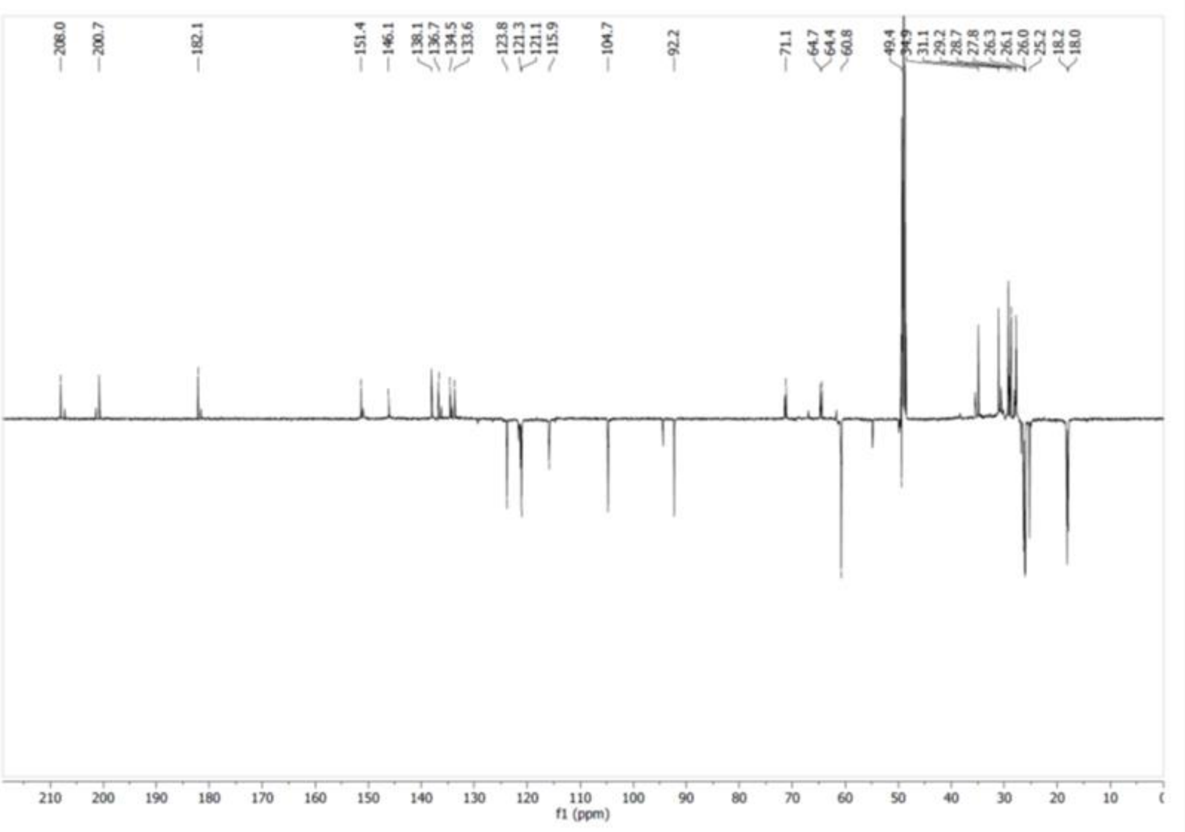

Supplementary Figure S28. ^{13}C-DEPTQ NMR spectrum of compound **4** in CD_3OD at 151 MHz.

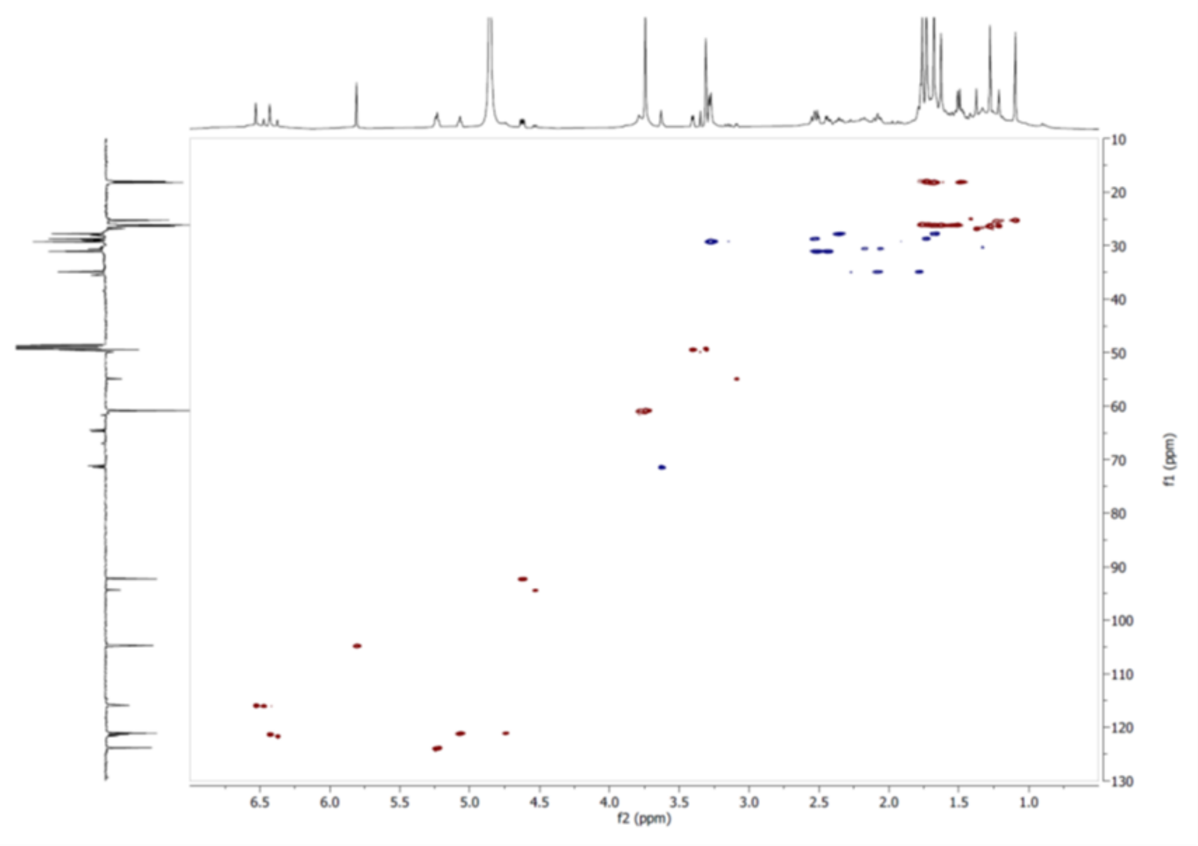

Supplementary Figure S29. Edited HSQC NMR spectrum of compound **4** in CD_3OD.

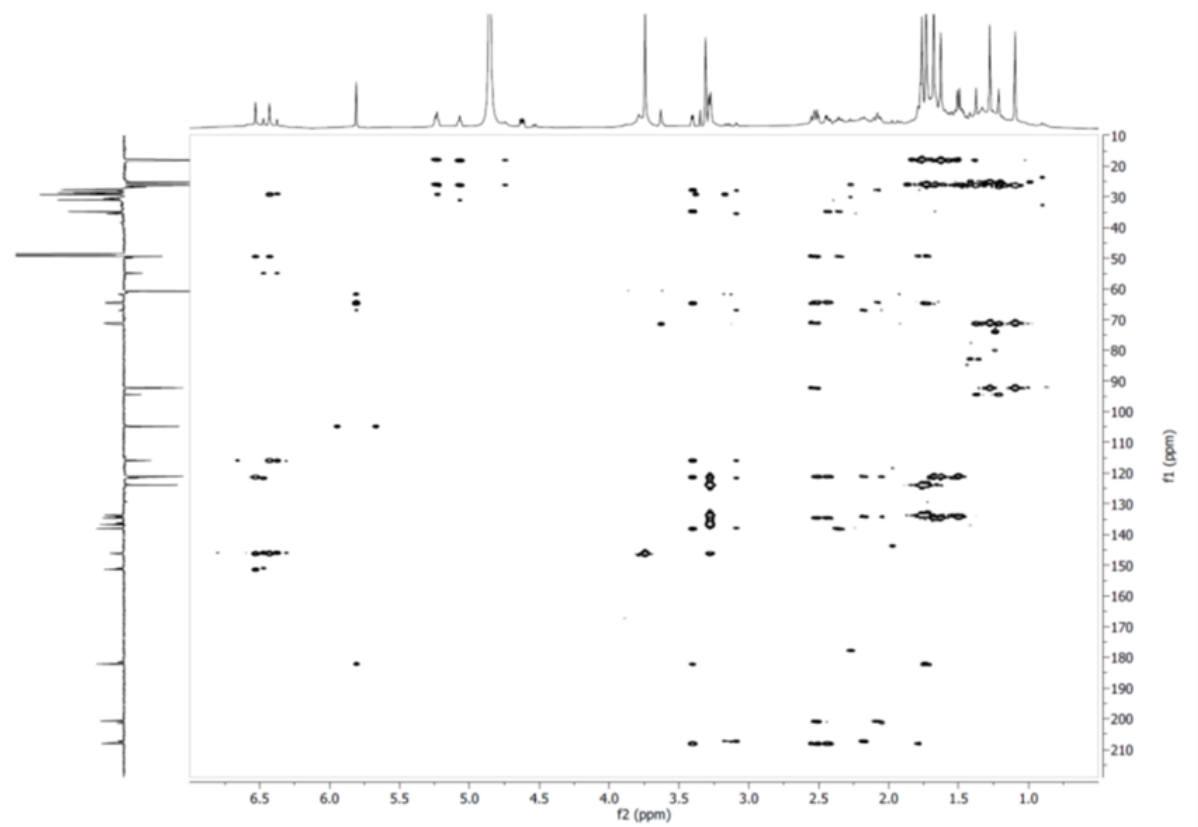

Supplementary Figure S30. HMBC NMR spectrum of compound **4** in CD_3OD.

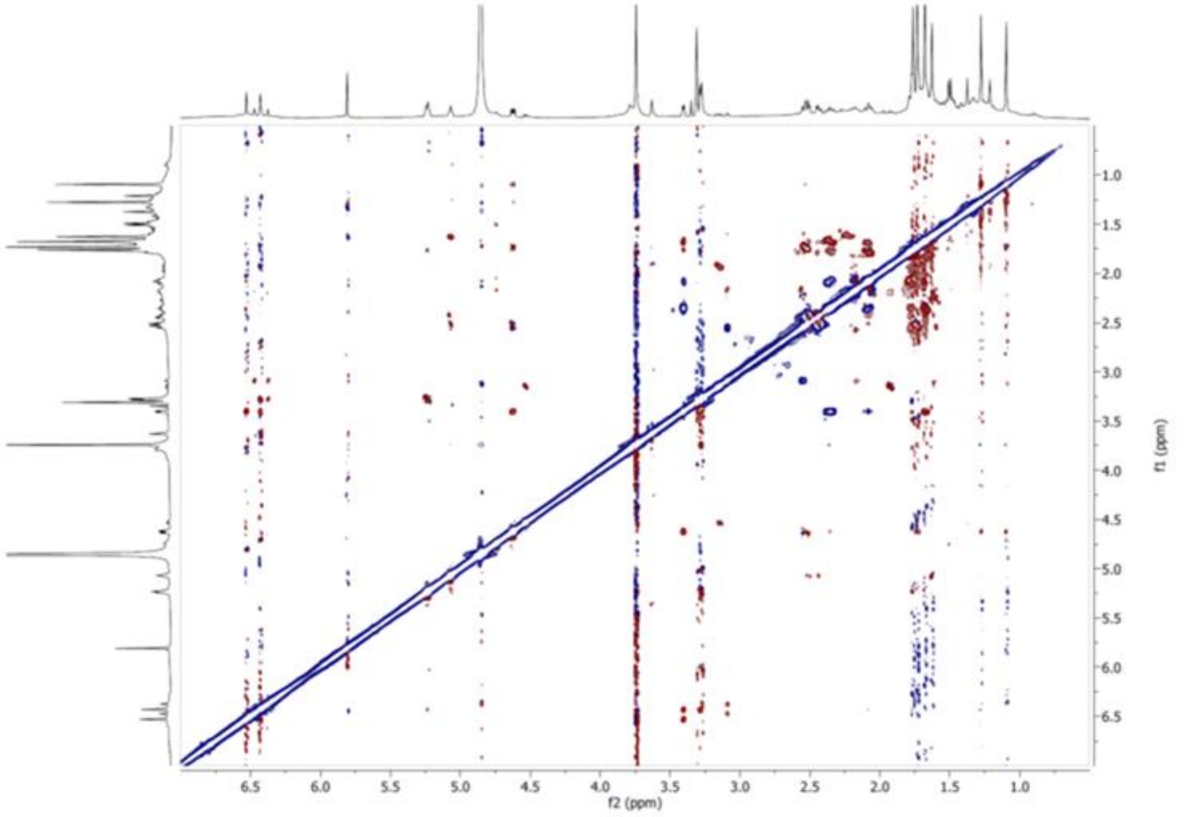

Supplementary Figure S31. ROESY NMR spectrum of compound **4** in CD_3OD.

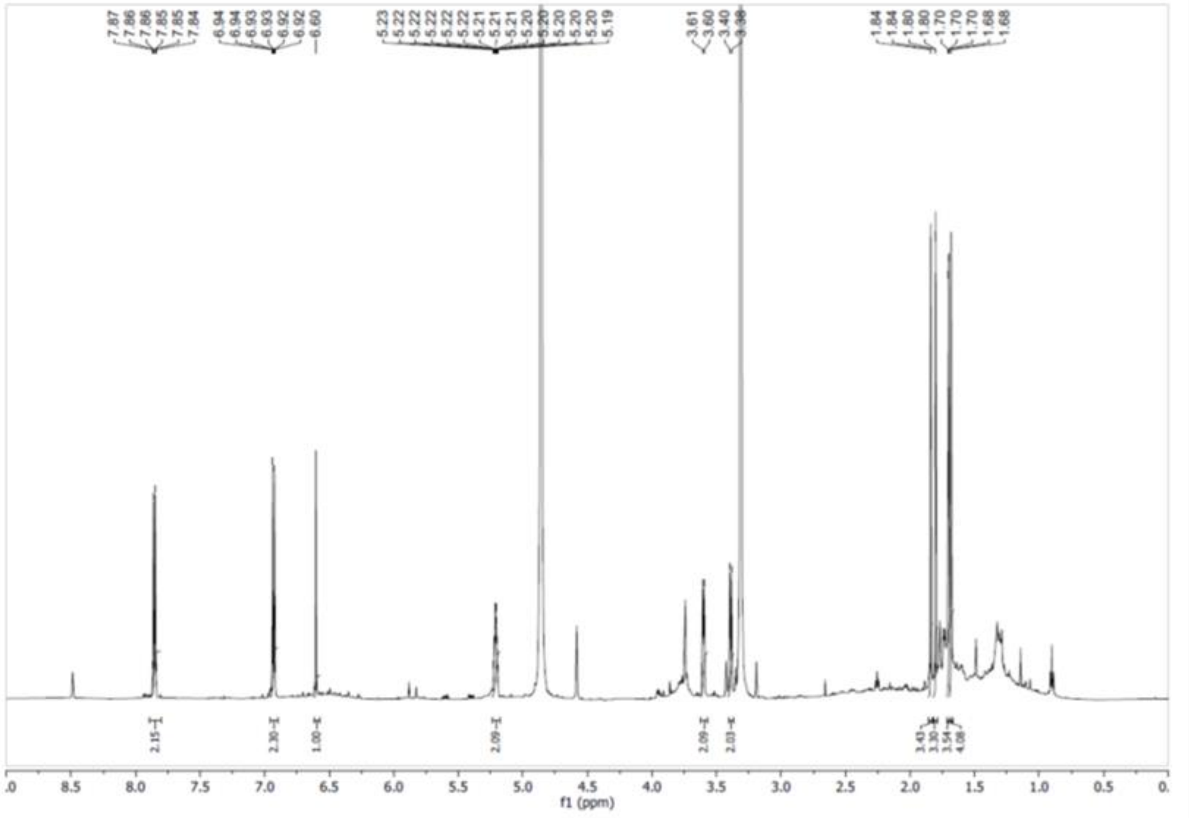

Supplementary Figure S32. ^1H NMR spectrum of compound **5** in CD_3OD at 600 MHz.

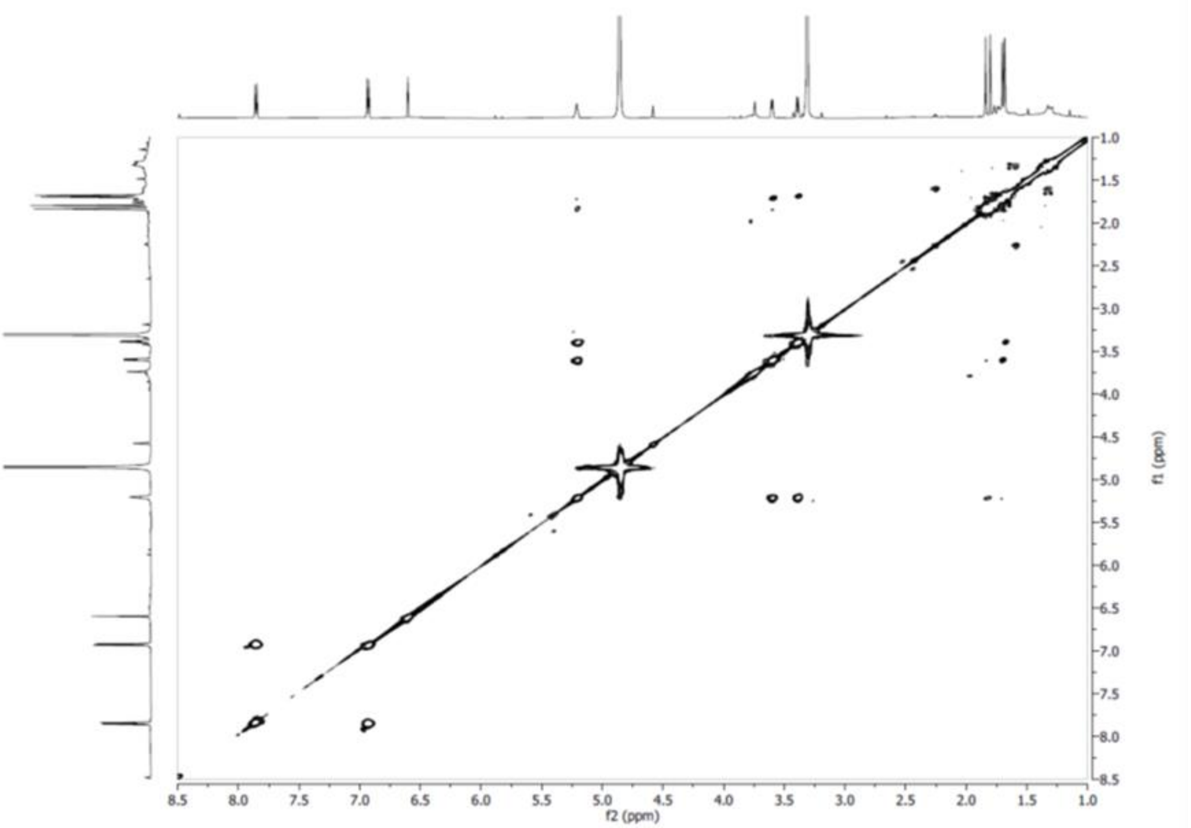

Supplementary Figure S33. COSY NMR spectrum of compound **5** in CD_3OD.

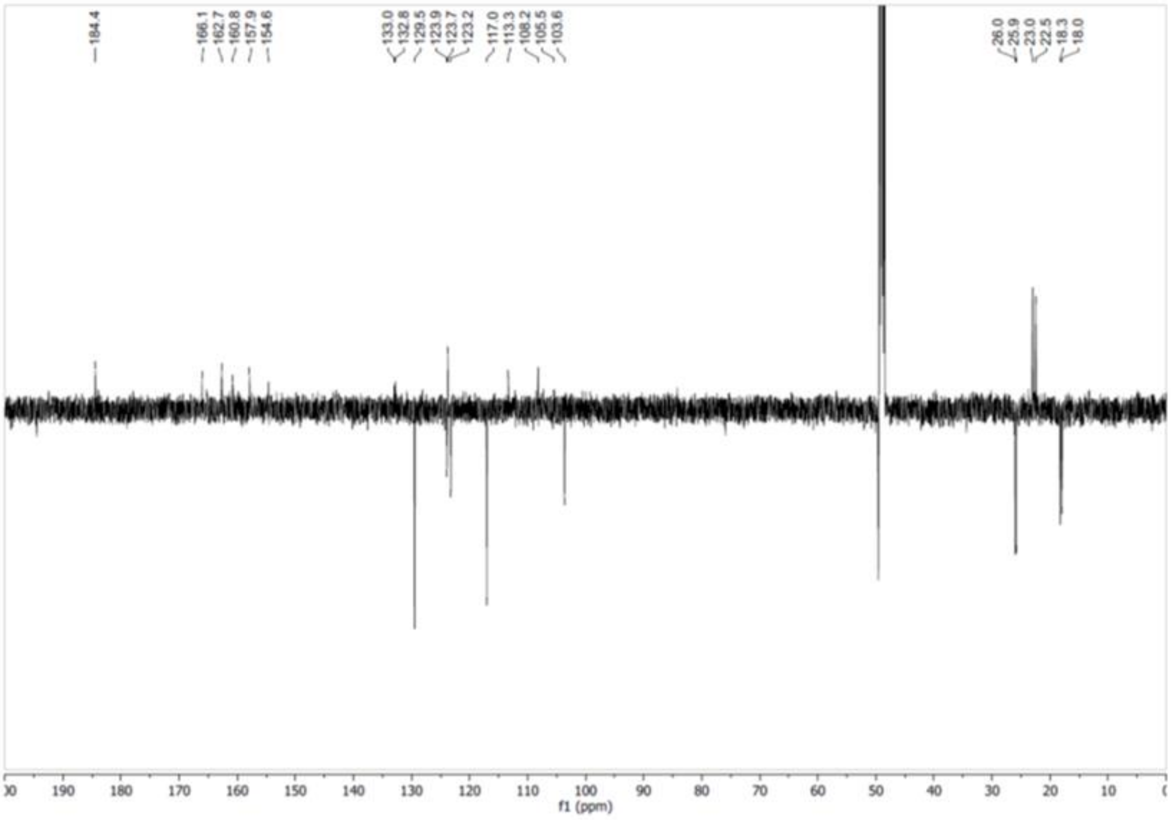

Supplementary Figure S34. ^{13}C-DEPTQ NMR spectrum of compound **5** in CD_3OD at 151 MHz.

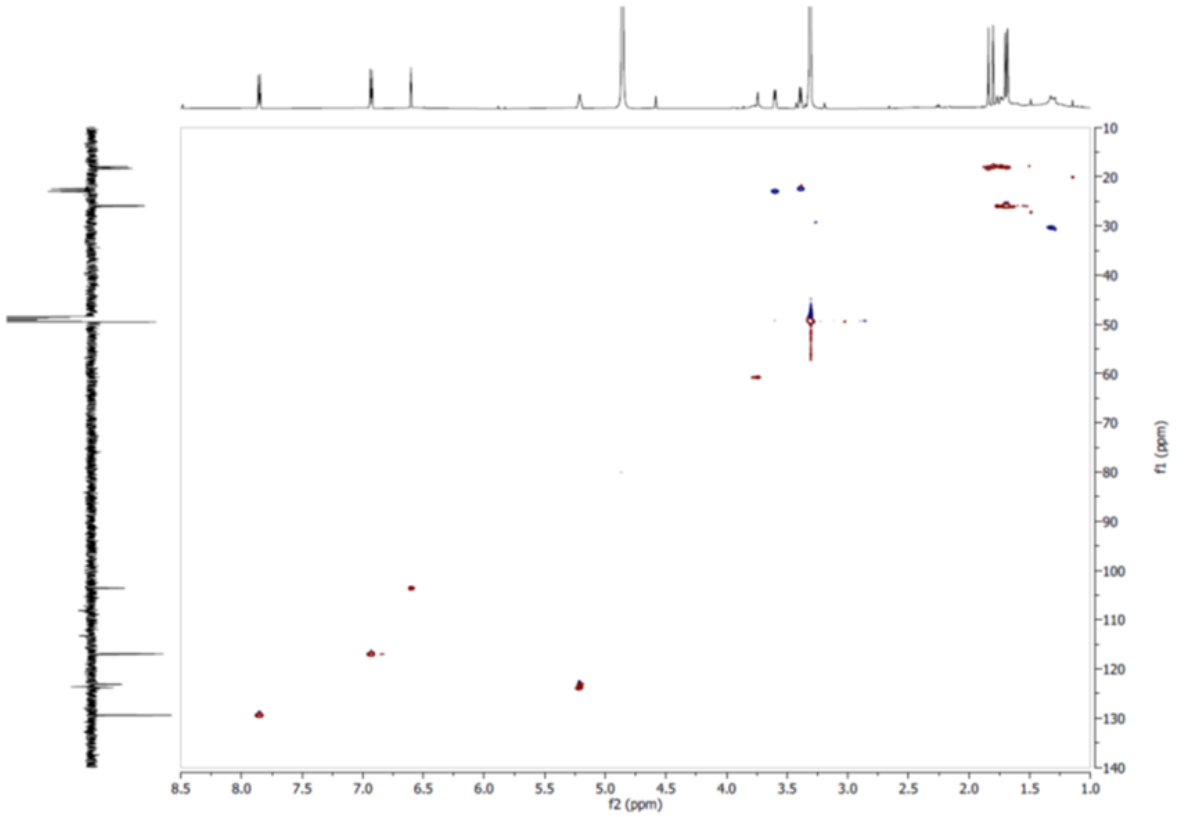

Supplementary Figure S35. Edited HSQC NMR spectrum of compound **5** in CD_3OD.

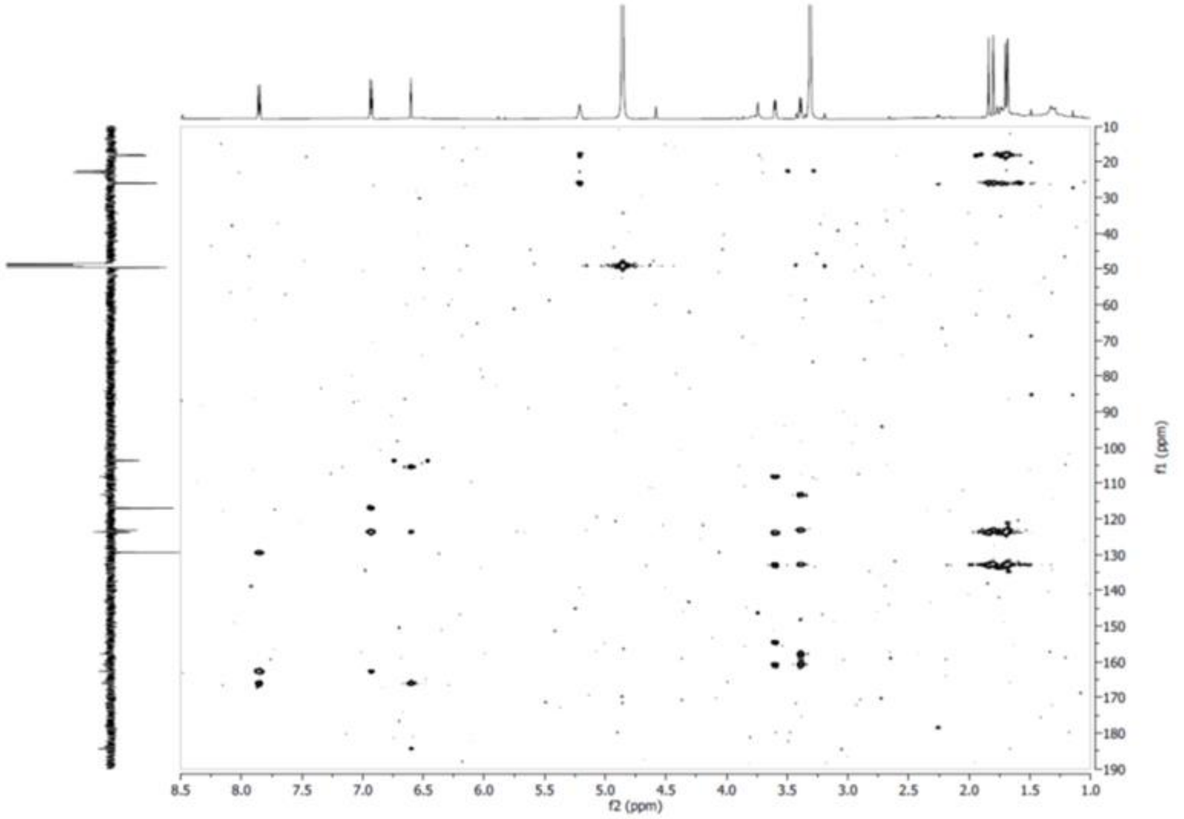

Supplementary Figure S36. HMBC NMR spectrum of compound **5** in CD_3OD.

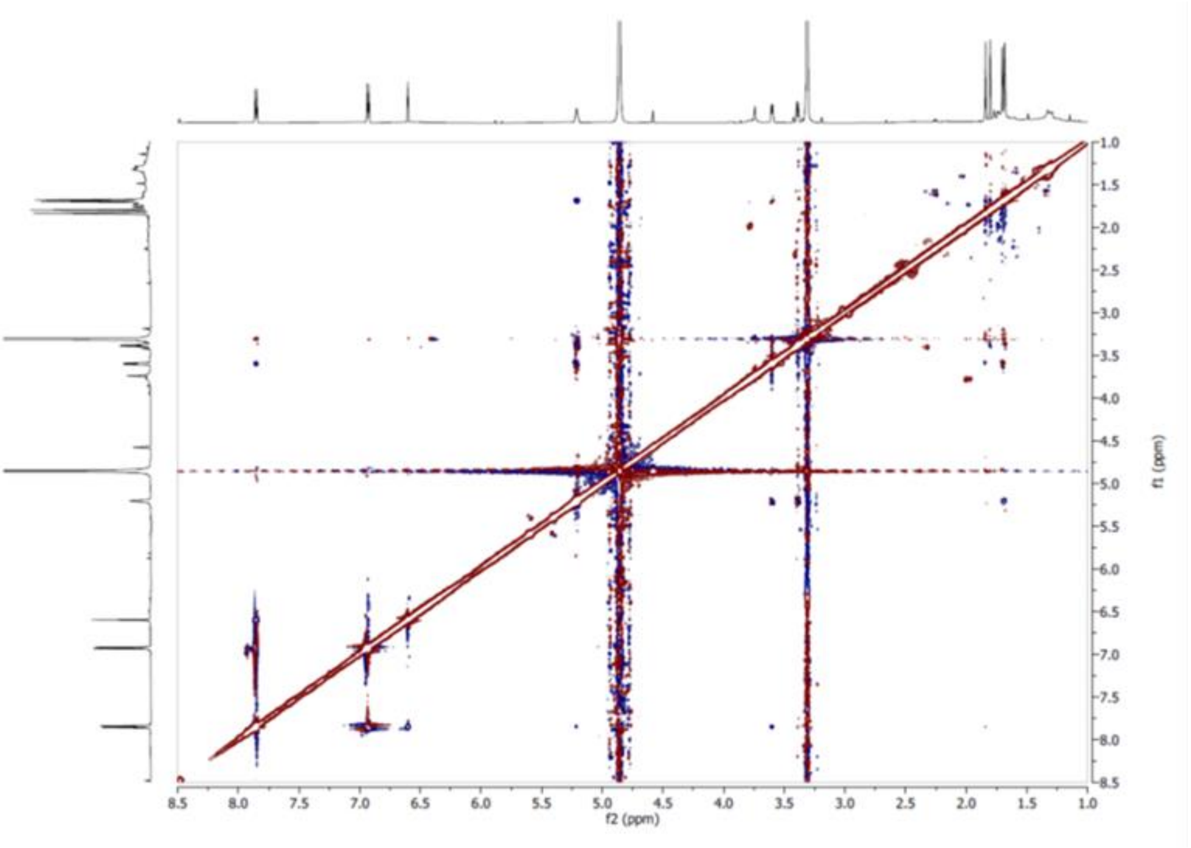

Supplementary Figure S37. ROESY NMR spectrum of compound **5** in CD_3OD.

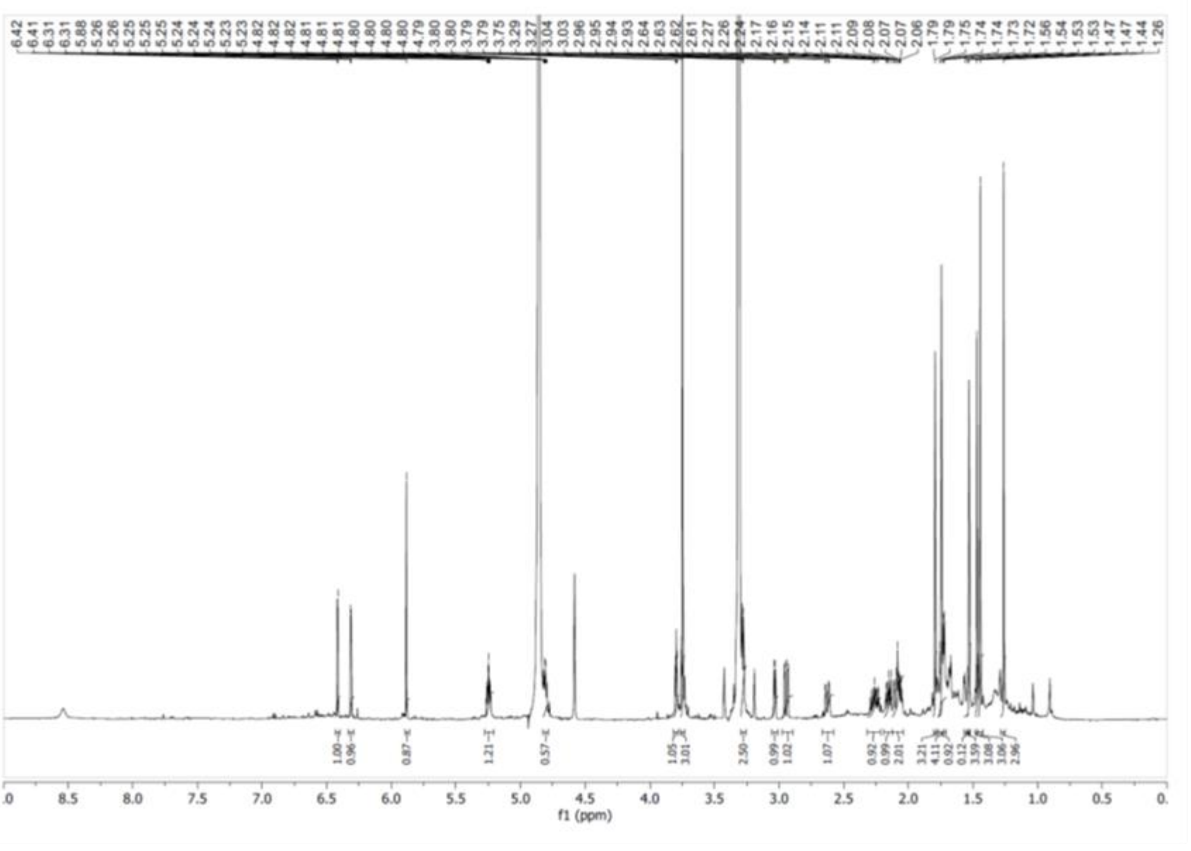

Supplementary Figure S38. ^1H NMR spectrum of compound **6** in CD_3OD at 600 MHz.

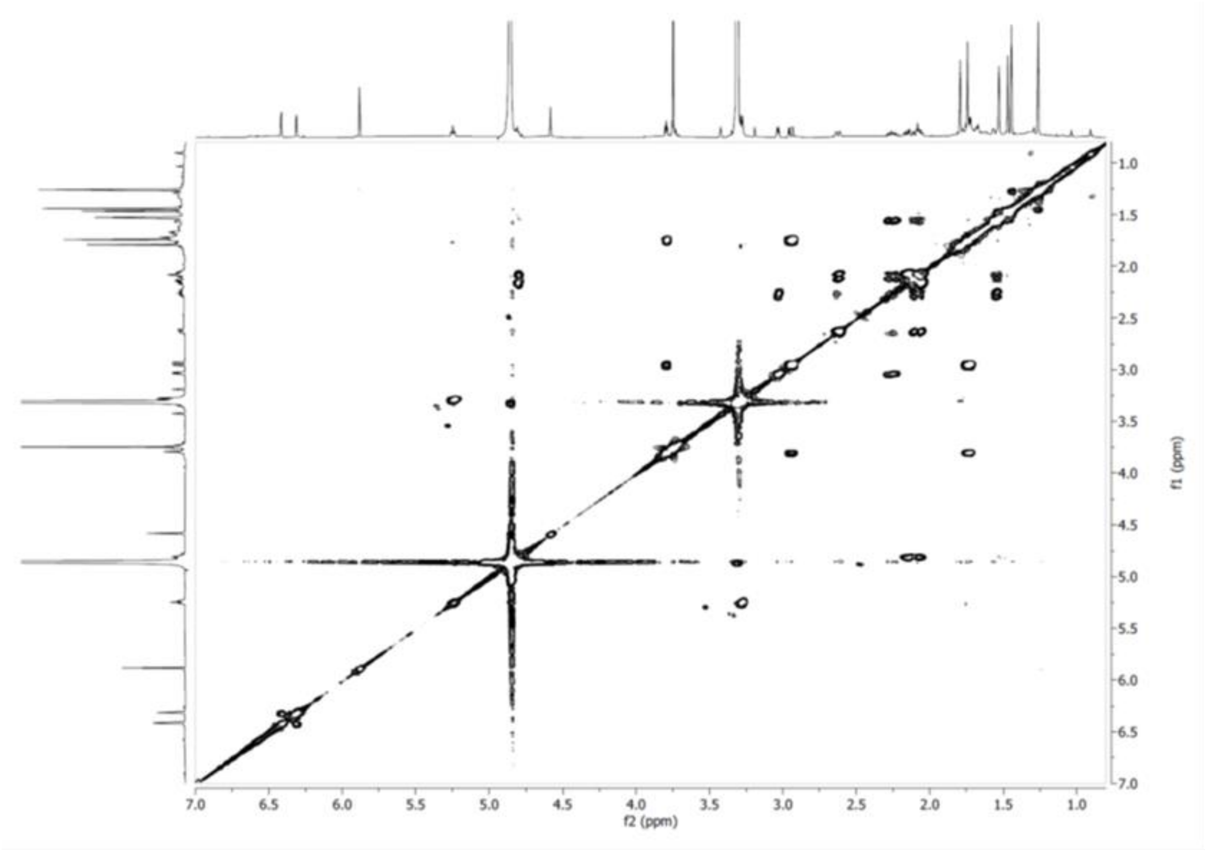

Supplementary Figure S39. COSY NMR spectrum of compound **6** in CD_3OD.

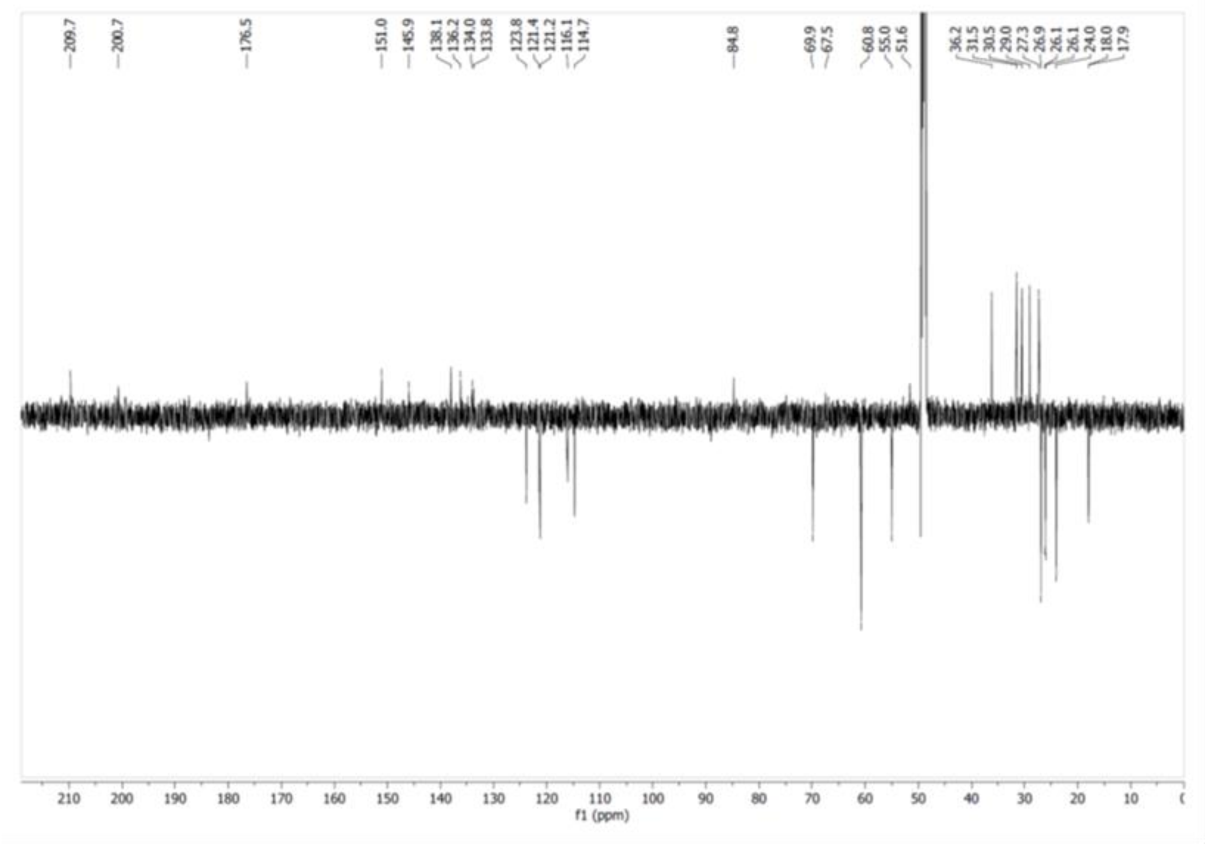

Supplementary Figure S40. ^{13}C-DEPTQ NMR spectrum of compound **6** in CD_3OD at 151 MHz.

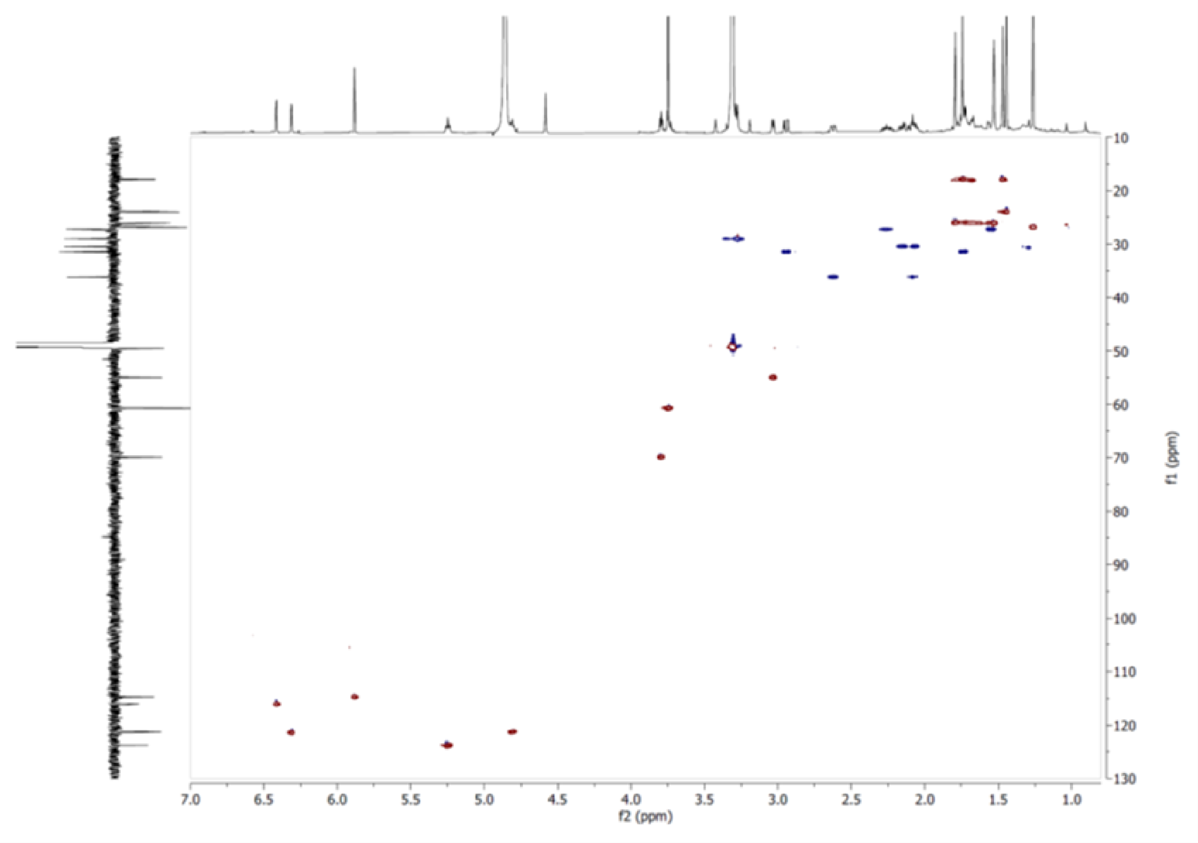

Supplementary Figure S41. Edited HSQC NMR spectrum of compound **6** in CD_3OD.

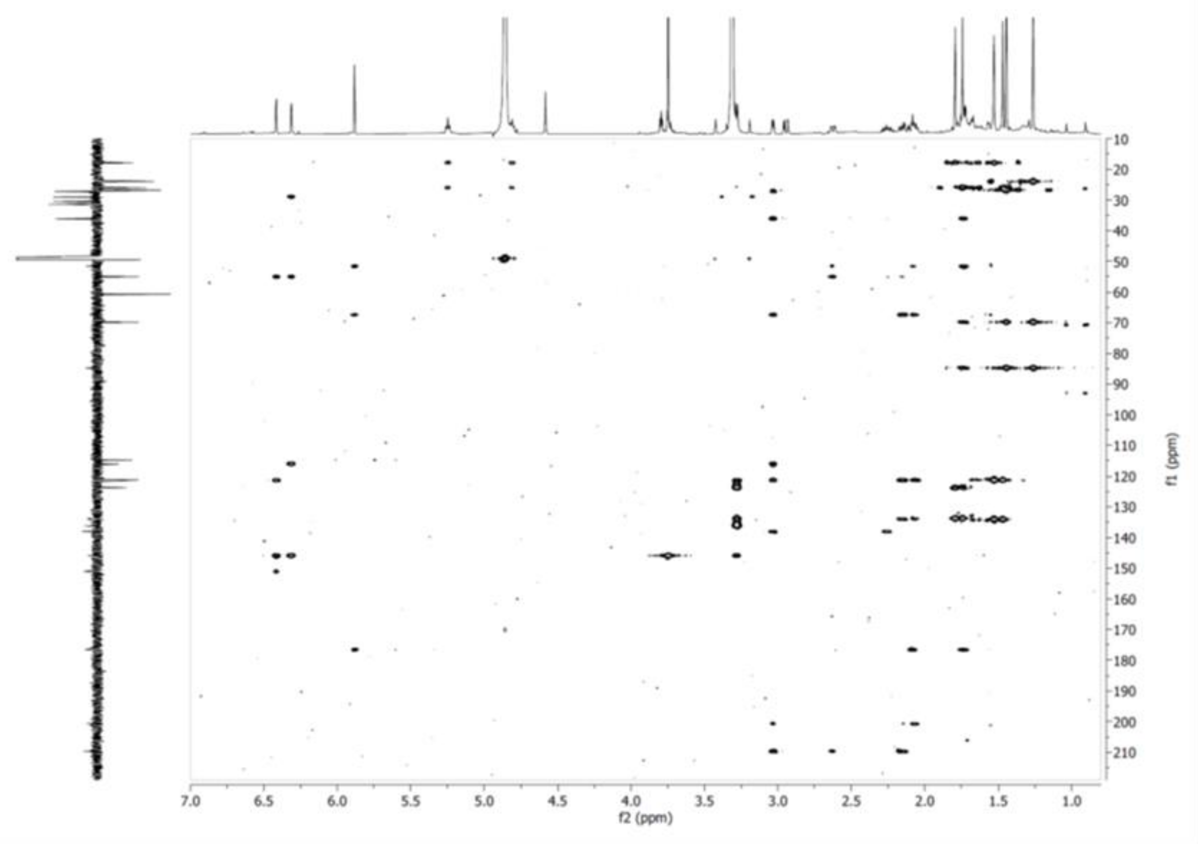

Supplementary Figure S42. HMBC NMR spectrum of compound **6** in CD_3OD.

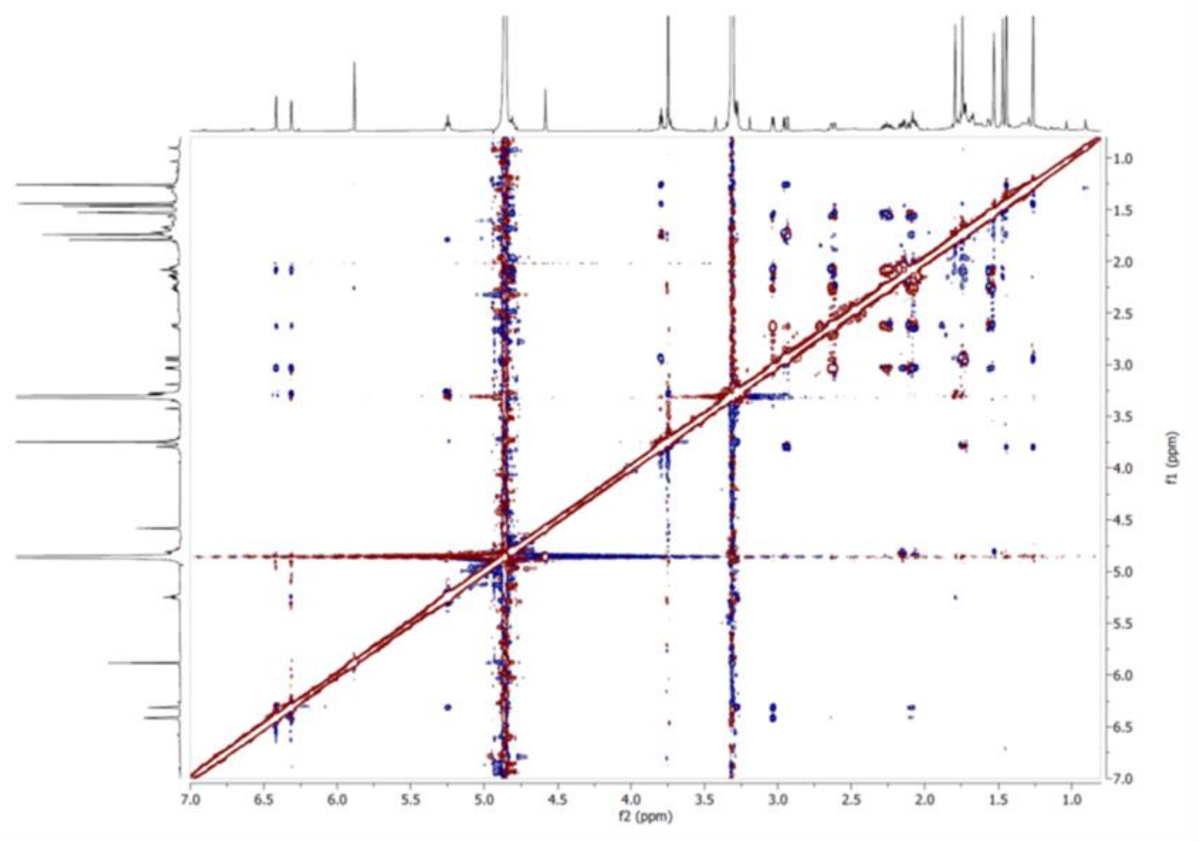

Supplementary Figure S43. ROESY NMR spectrum of compound **6** in CD_3OD.

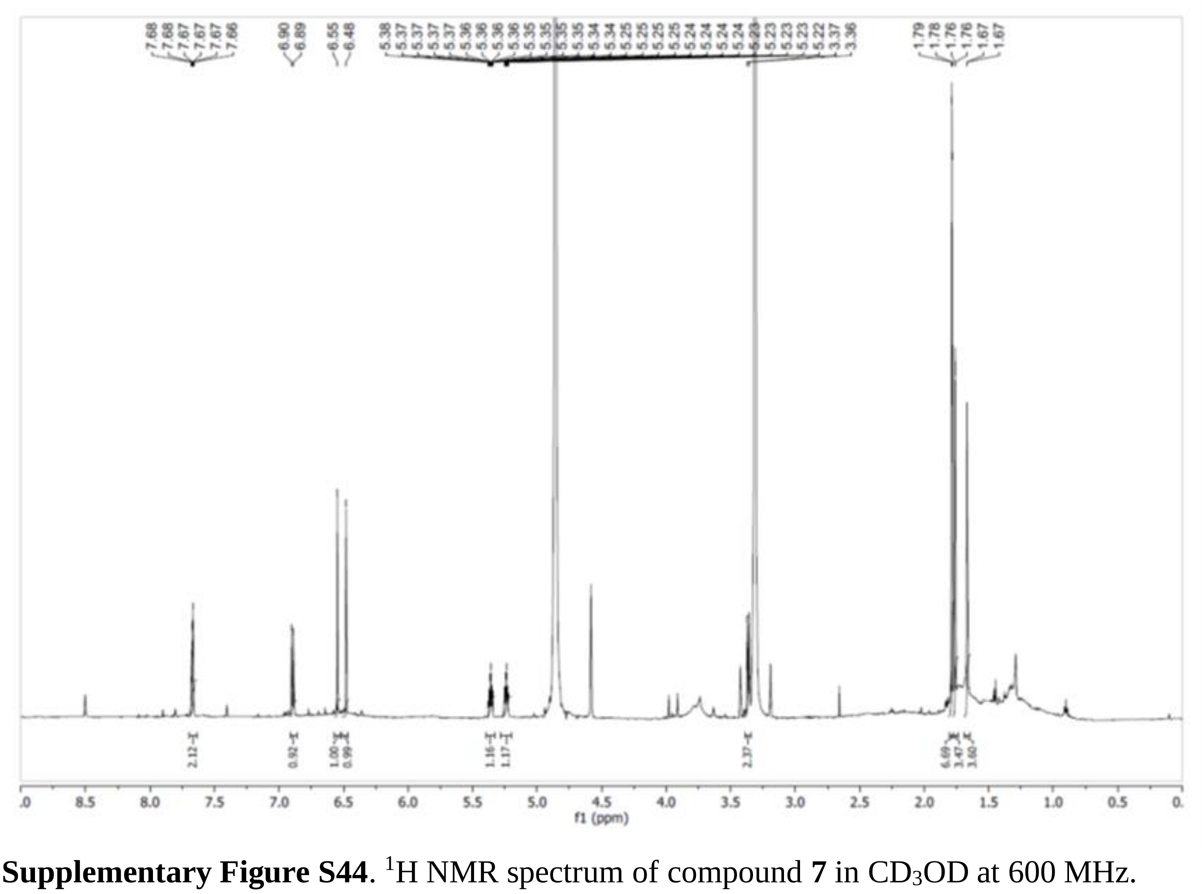

Supplementary Figure S44. ^{1}H NMR spectrum of compound **7** in CD_3OD at 600 MHz.

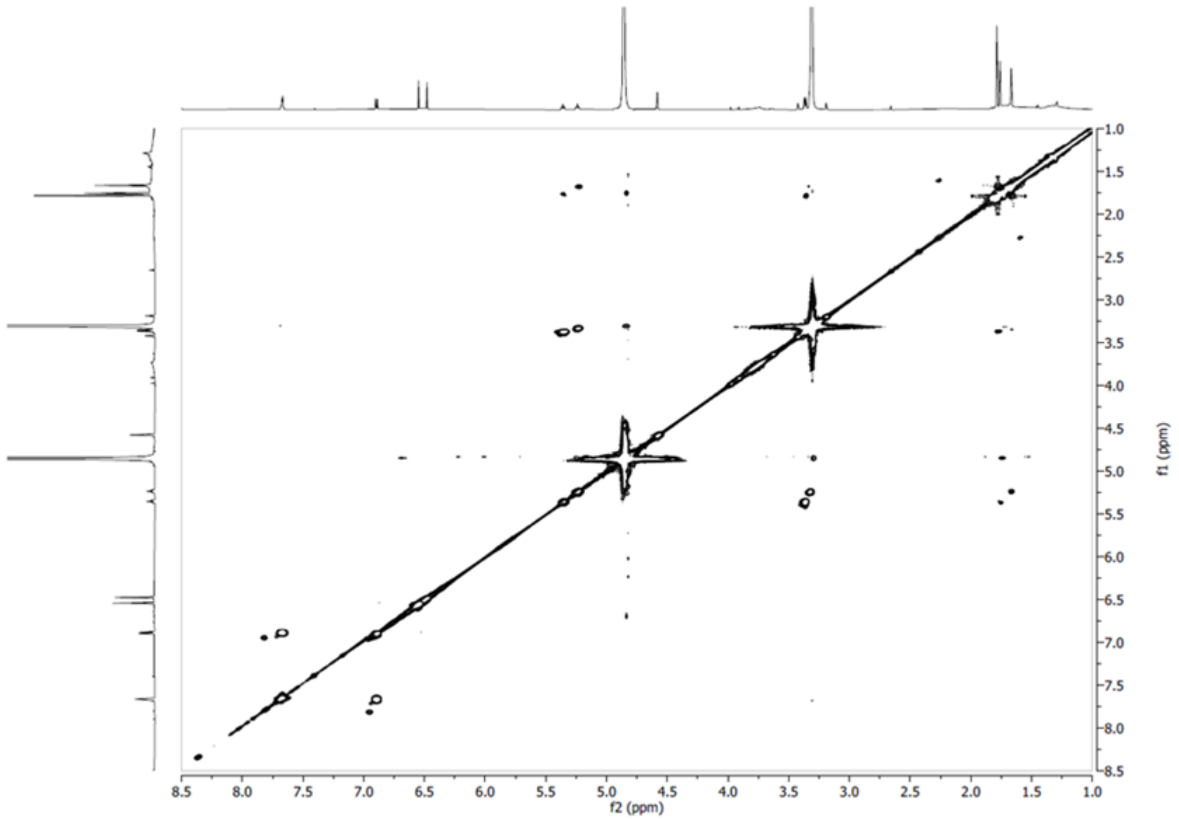

Supplementary Figure S45. COSY NMR spectrum of compound **7** in CD_3OD.

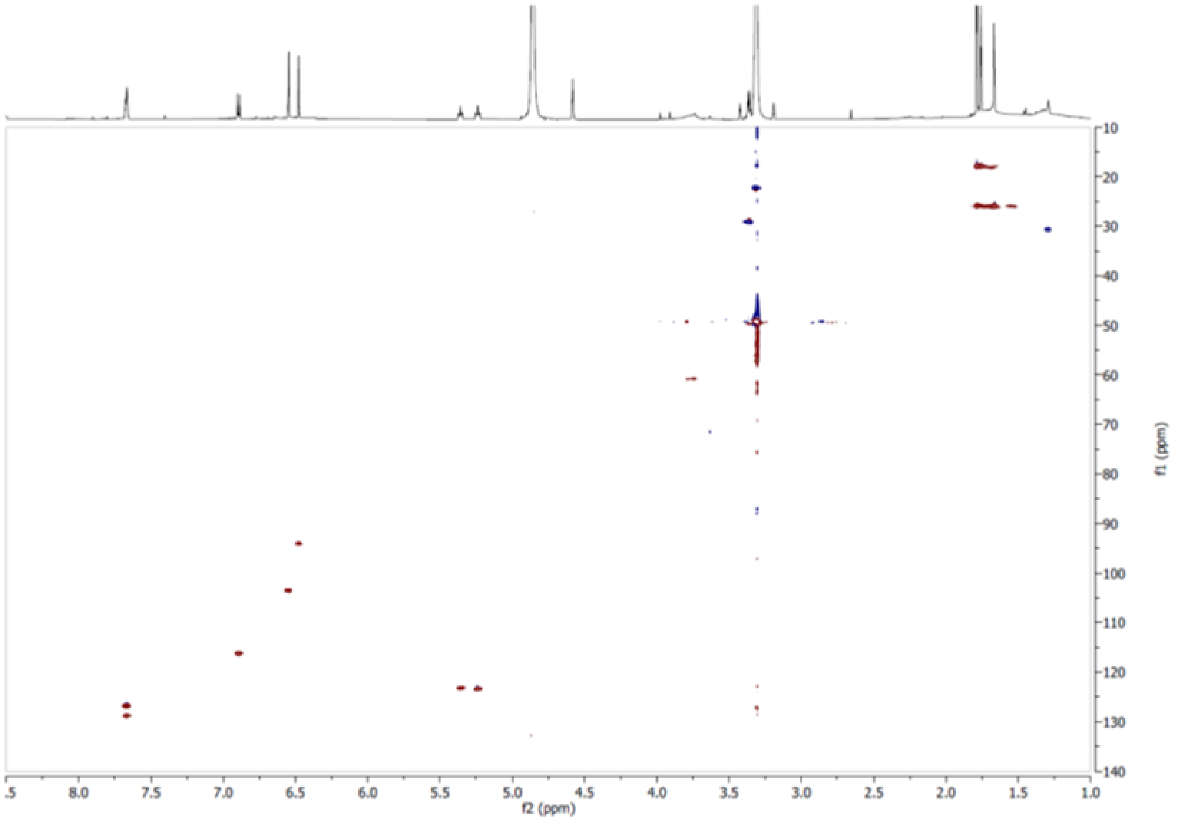

Supplementary Figure S46. Edited HSQC NMR spectrum of compound **7** in CD_3OD.

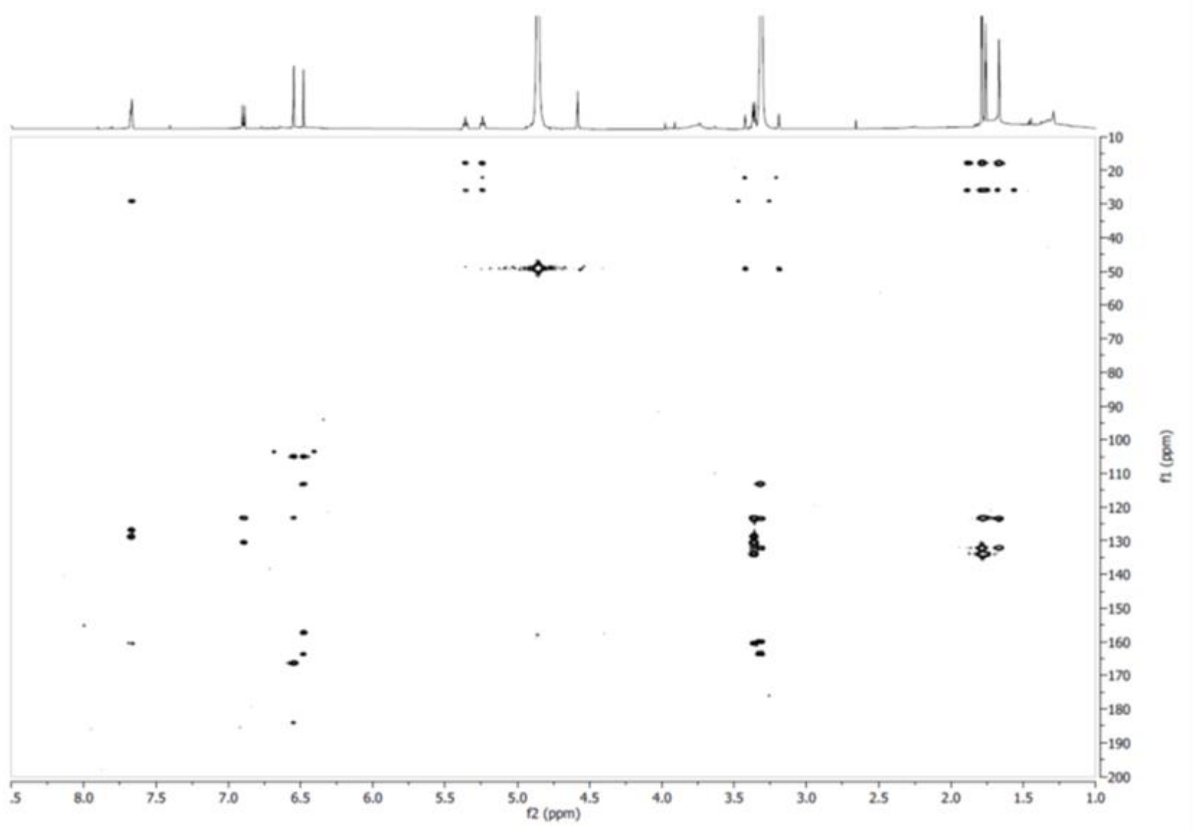

Supplementary Figure S47. HMBC NMR spectrum of compound **7** in CD_3OD.

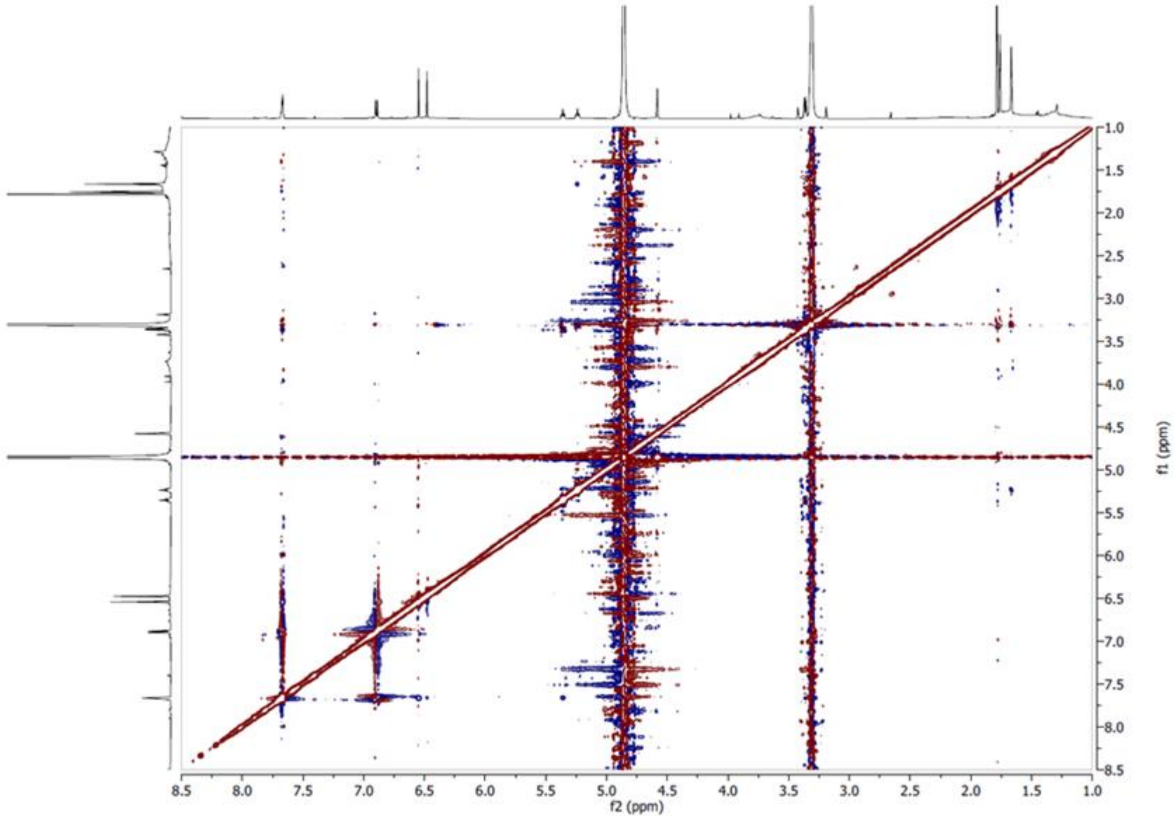

Supplementary Figure S48. ROESY NMR spectrum of compound **7** in CD_3OD.

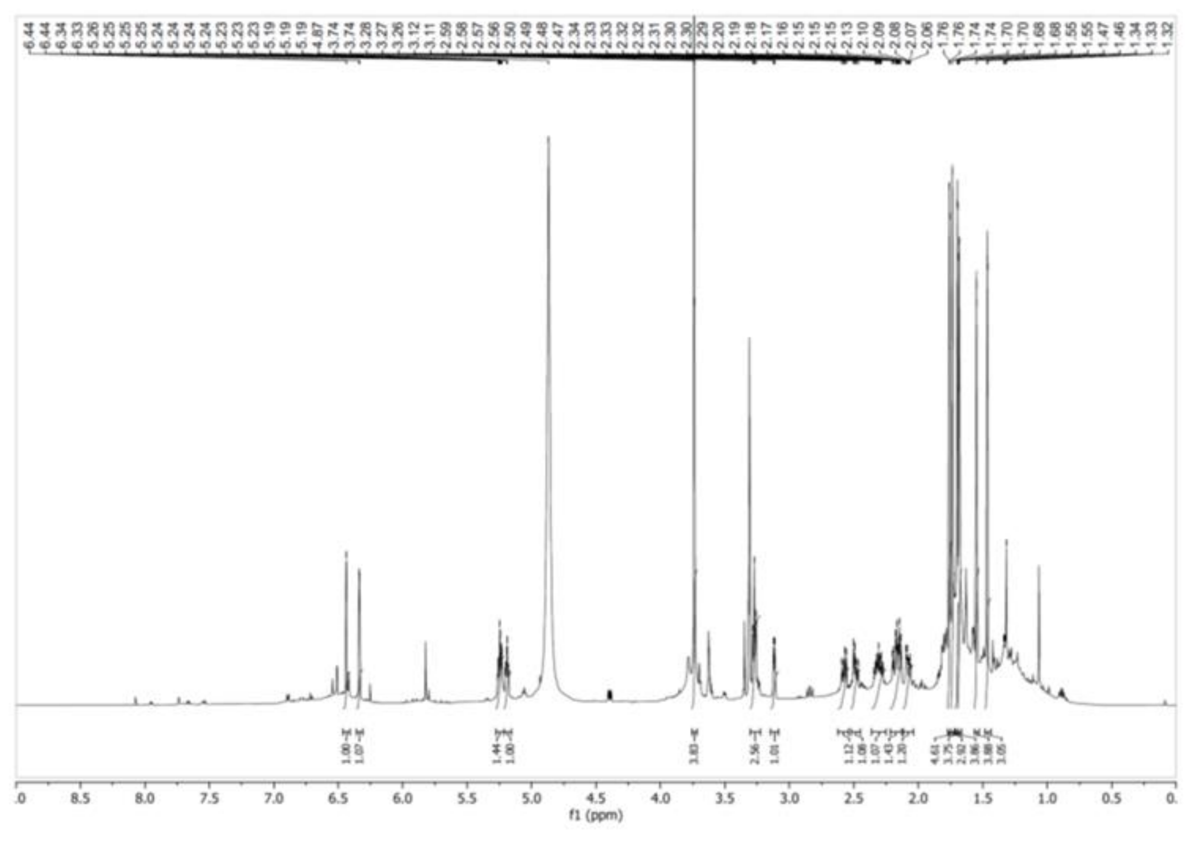

Supplementary Figure S49. ^{1}H NMR spectrum of compound **8** in CD_3OD at 600 MHz.

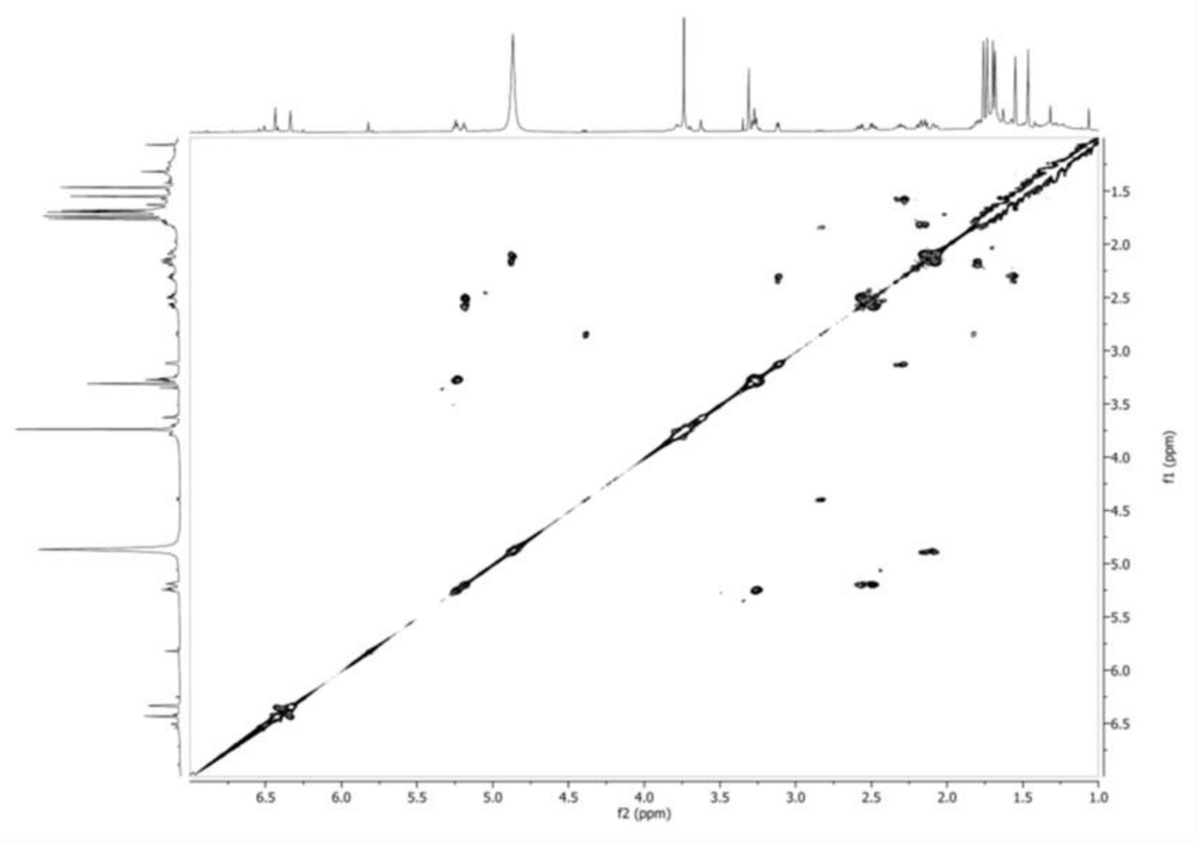

Supplementary Figure S50. COSY NMR spectrum of compound **8** in CD_3OD.

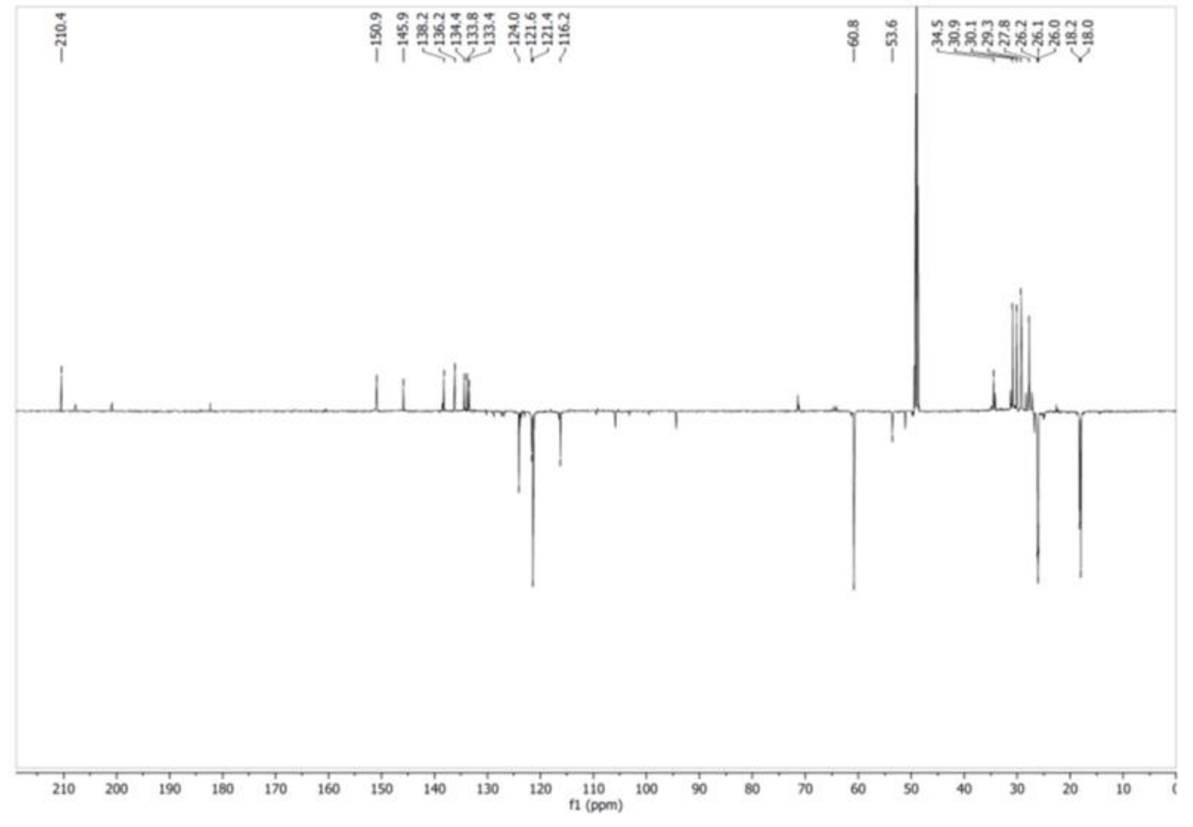

Supplementary Figure S51.^{13}C-DEPTQ NMR spectrum of compound **8** in CD_3OD at 151 MHz.

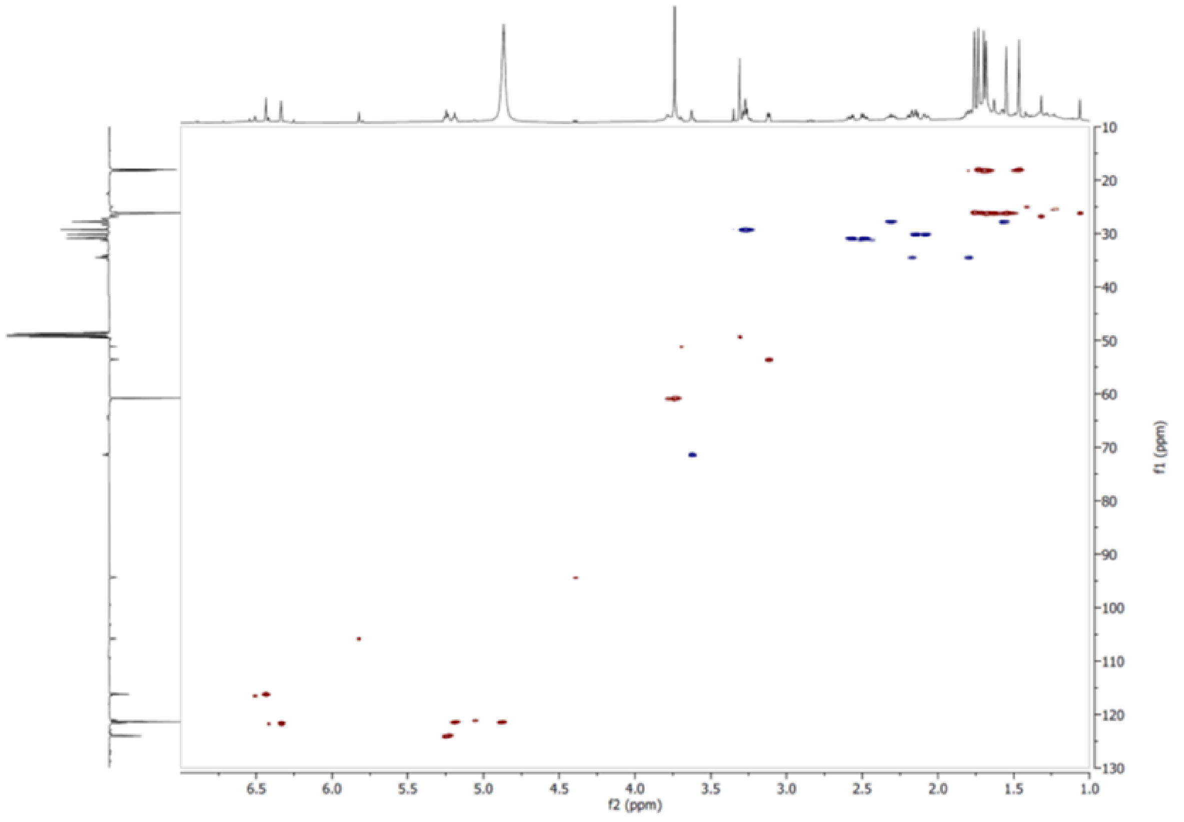

Supplementary Figure S52. Edited HSQC NMR spectrum of compound **8** in CD_3OD.

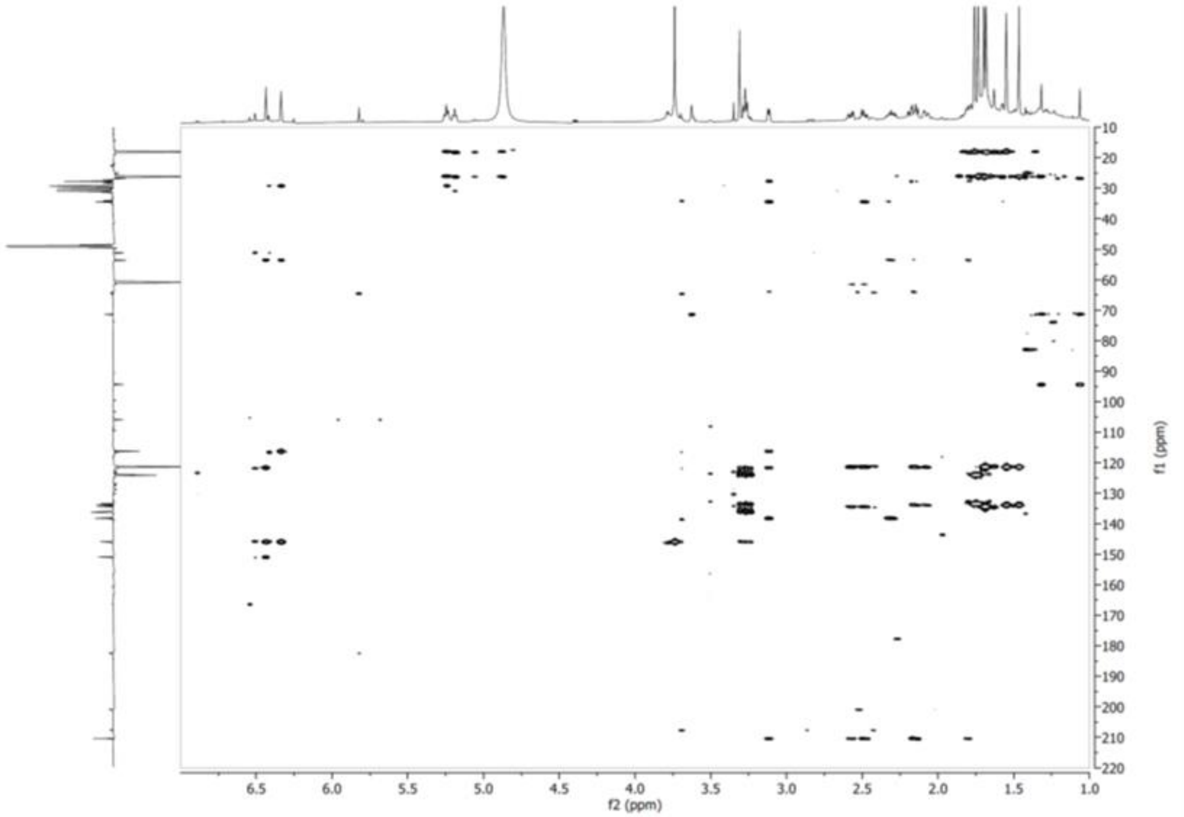

Supplementary Figure S53. HMBC NMR spectrum of compound **8** in CD_3OD.

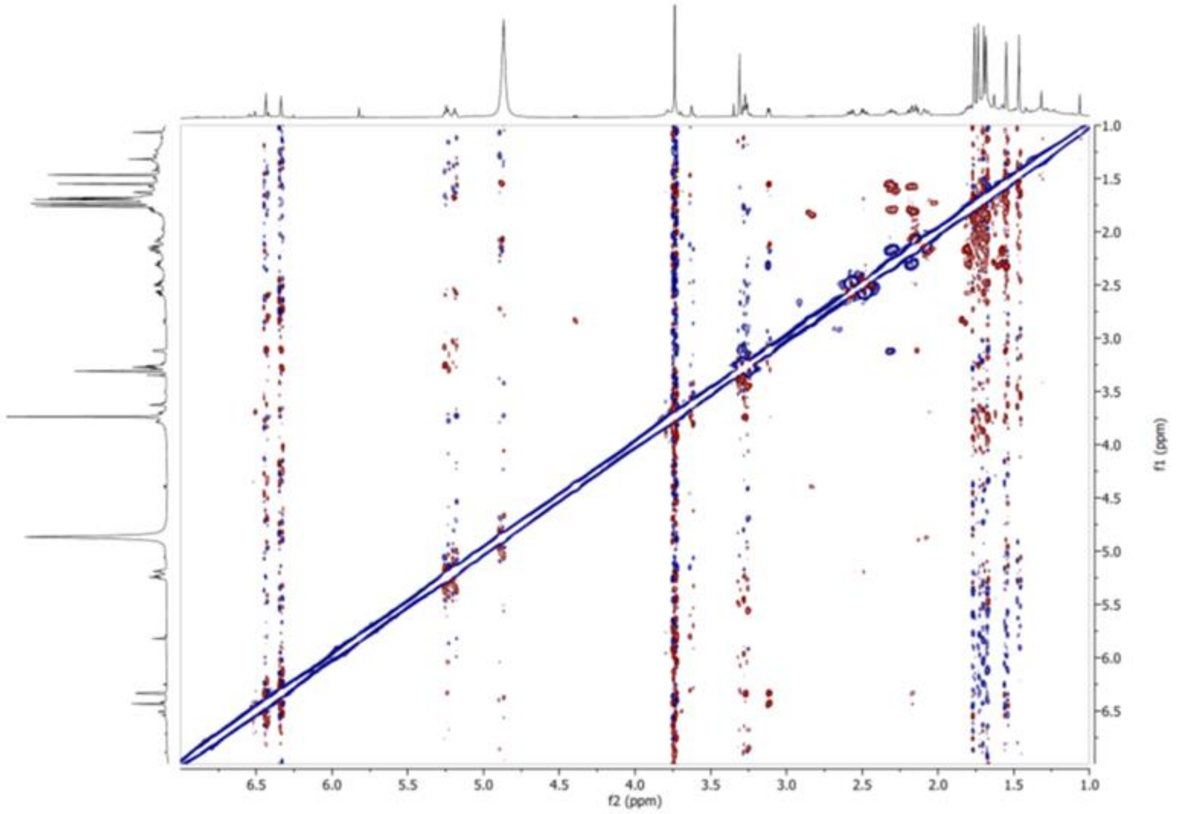

Supplementary Figure S54. ROESY NMR spectrum of compound **8** in CD_3OD.

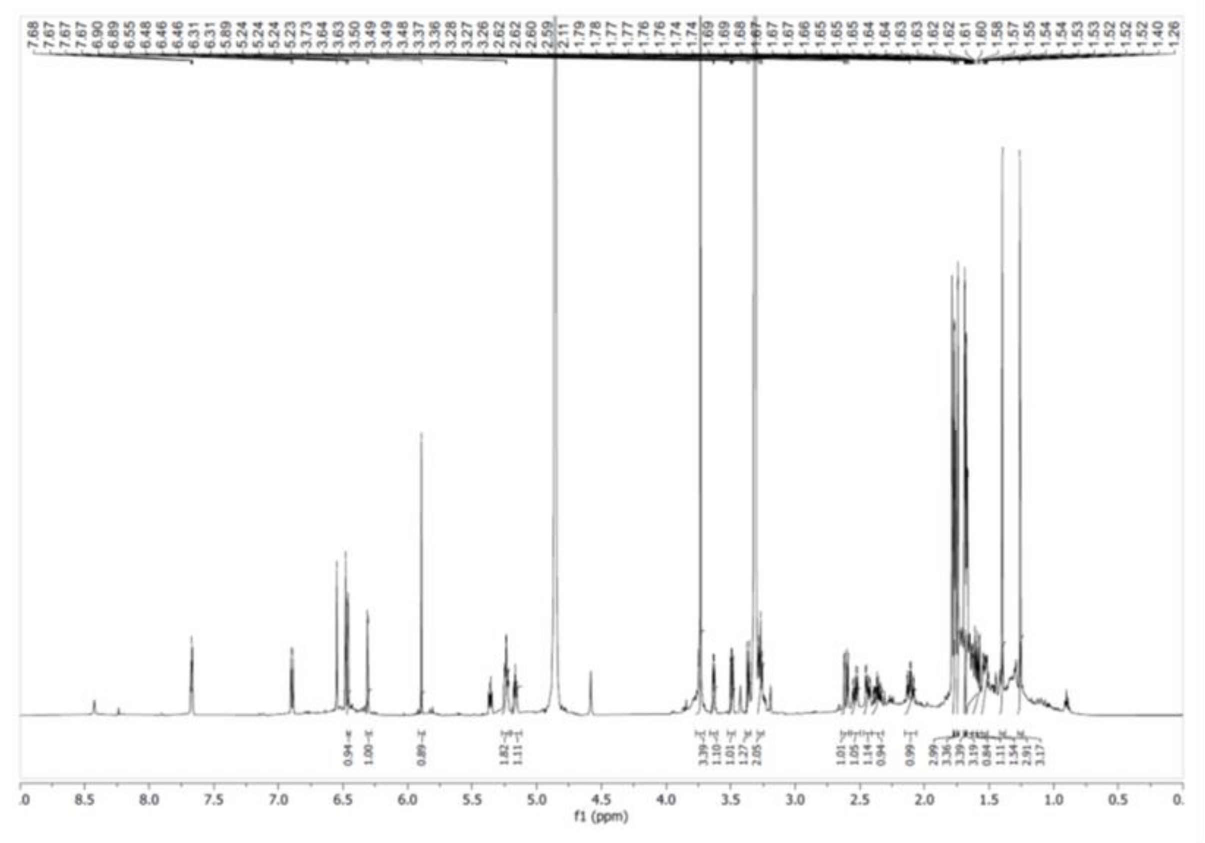

Supplementary Figure S55. ^{1}H NMR spectrum of compound **9** in CD_3OD at 600 MHz.

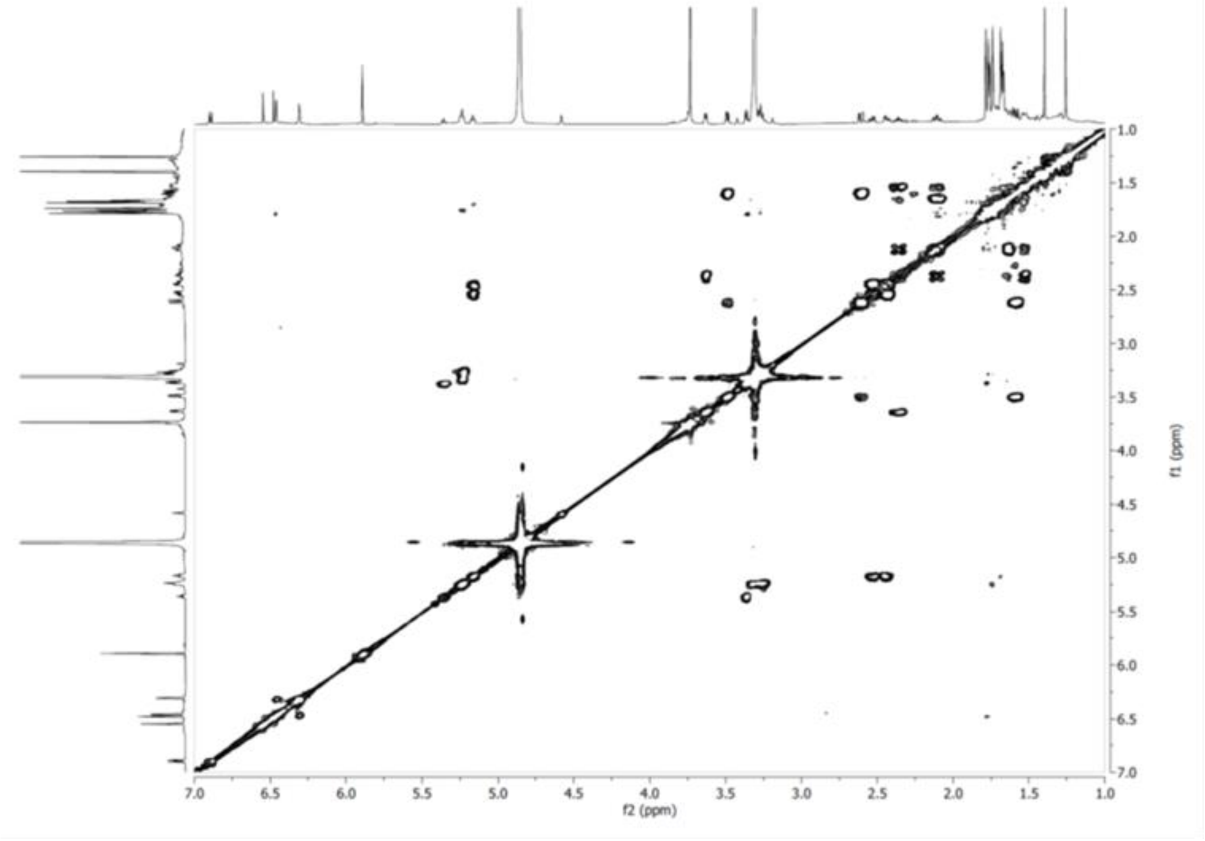

Supplementary Figure S56. COSY NMR spectrum of compound **9** in CD_3OD.

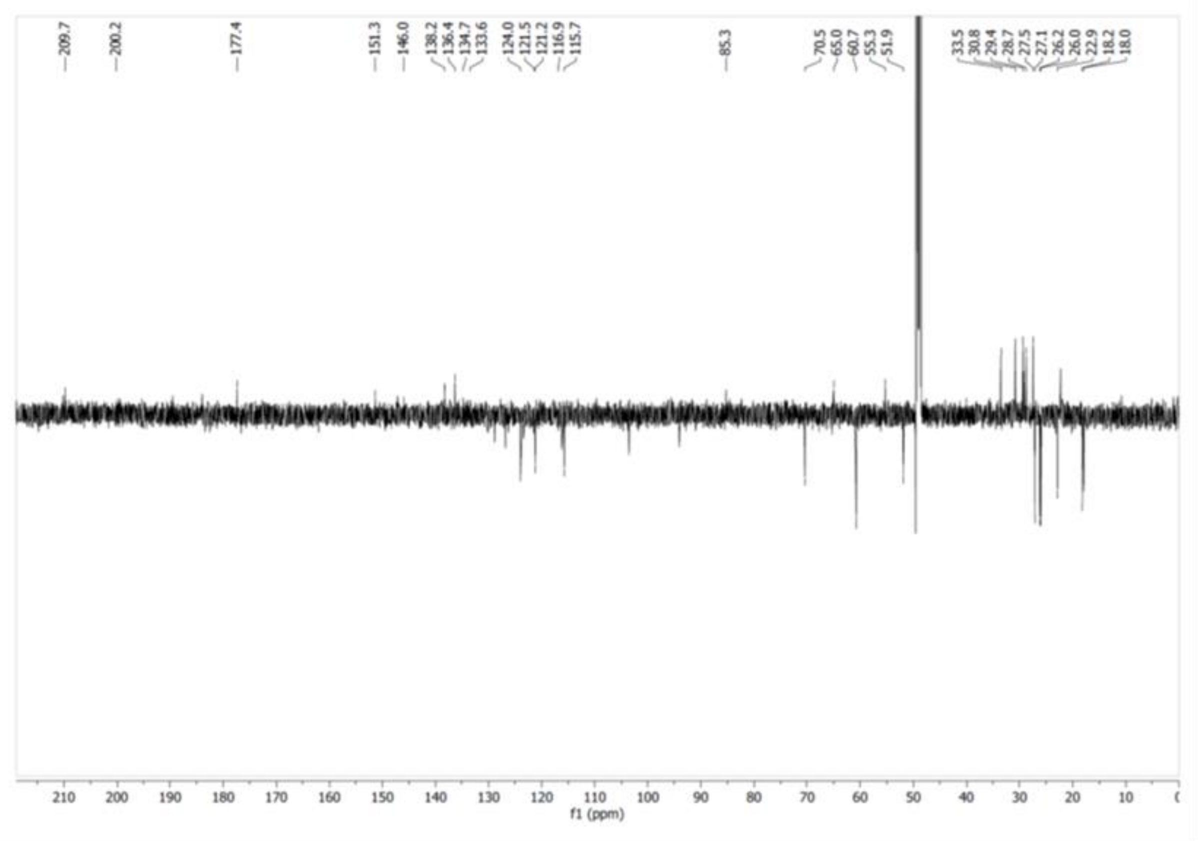

Supplementary Figure S57. ^{13}C-DEPTQ NMR spectrum of compound **9** in CD_3OD at 151 MHz.

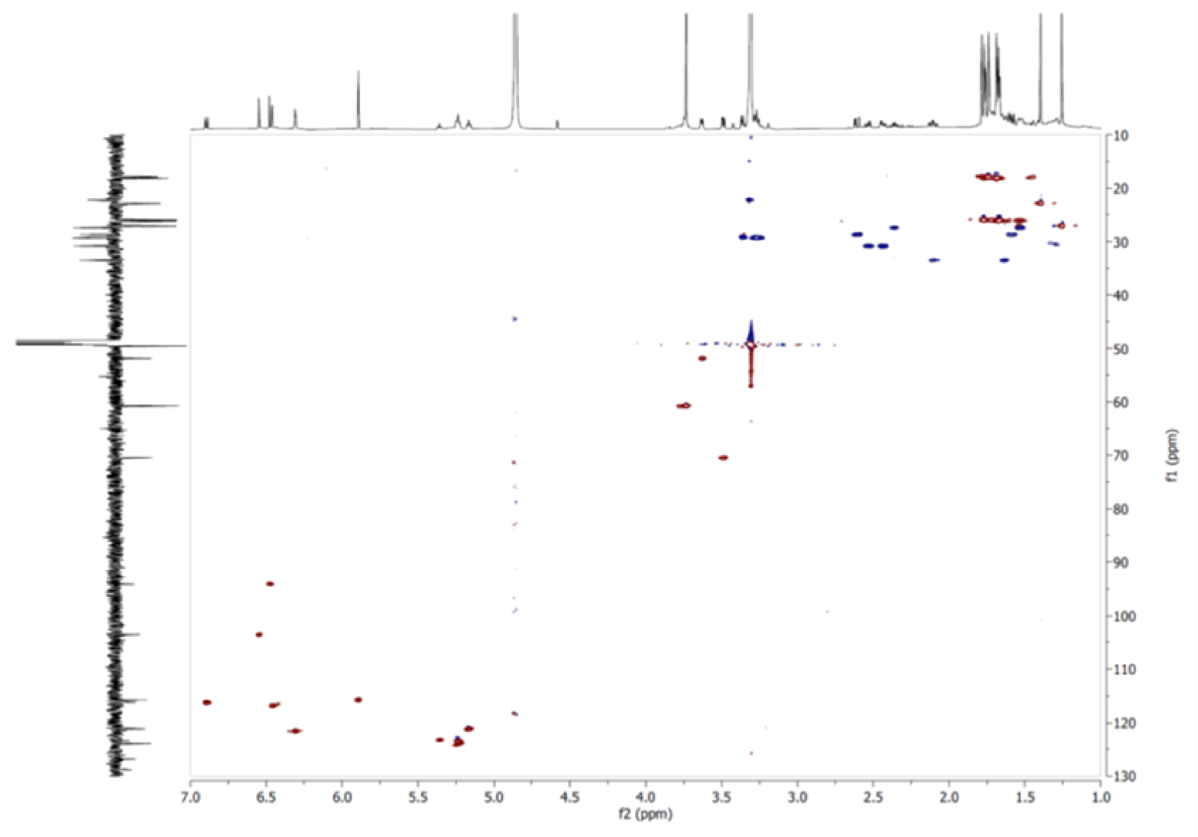

Supplementary Figure S58. Edited HSQC NMR spectrum of compound **9** in CD_3OD.

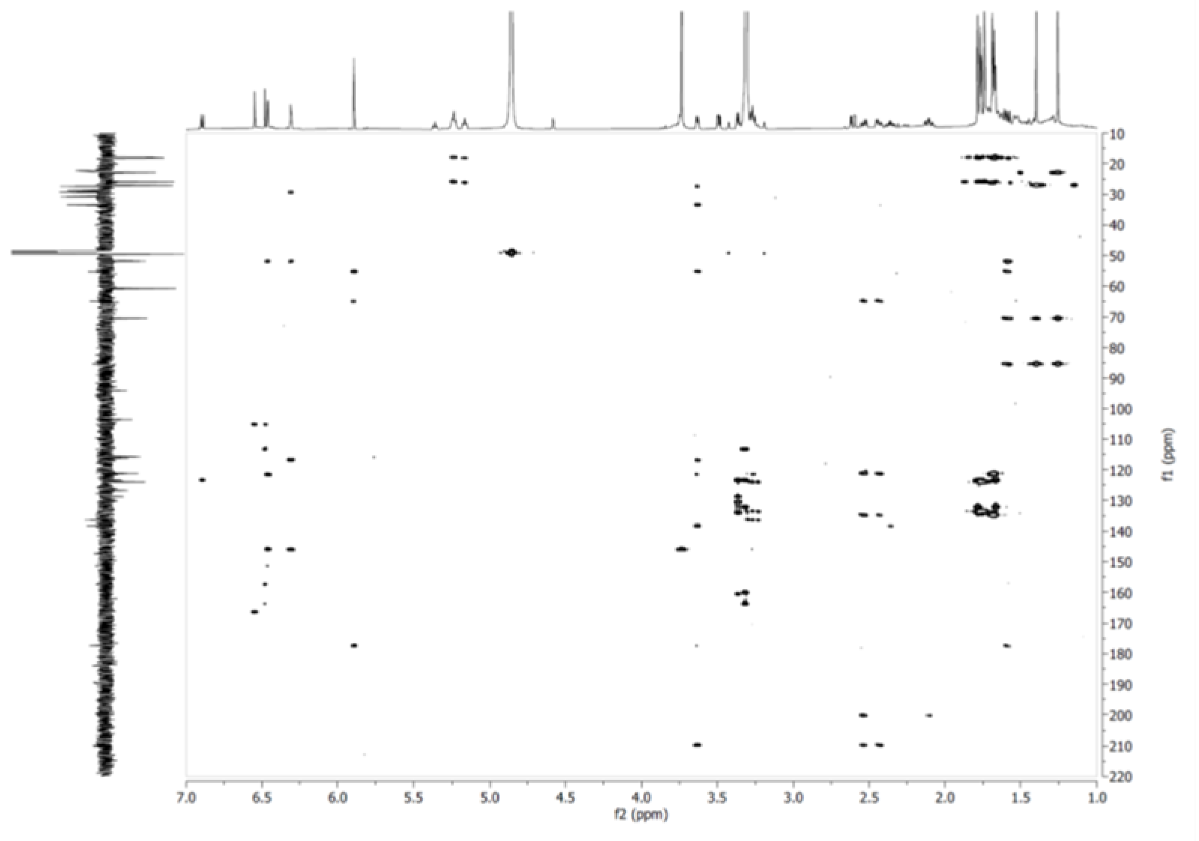

Supplementary Figure S59. HMBC NMR spectrum of compound **9** in CD_3OD.

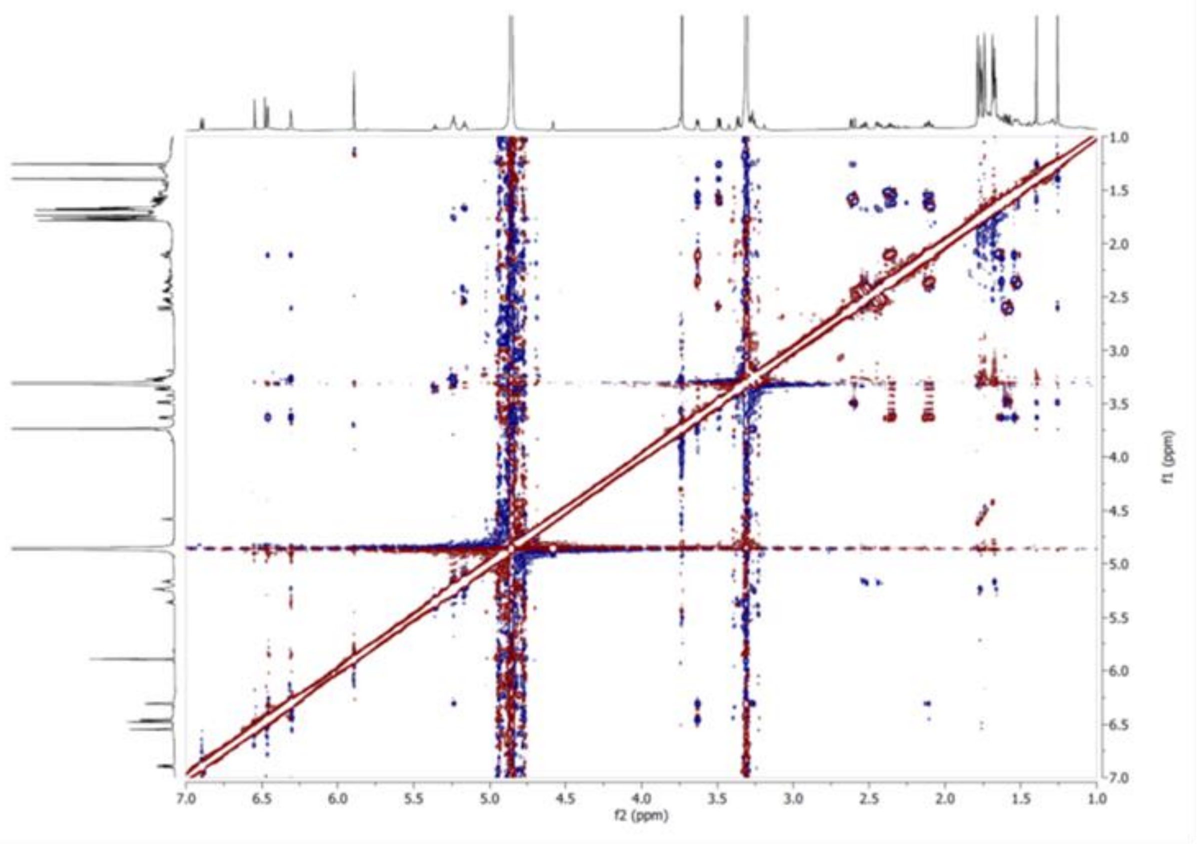

Supplementary Figure S60. ROESY NMR spectrum of compound **9** in CD_3OD.

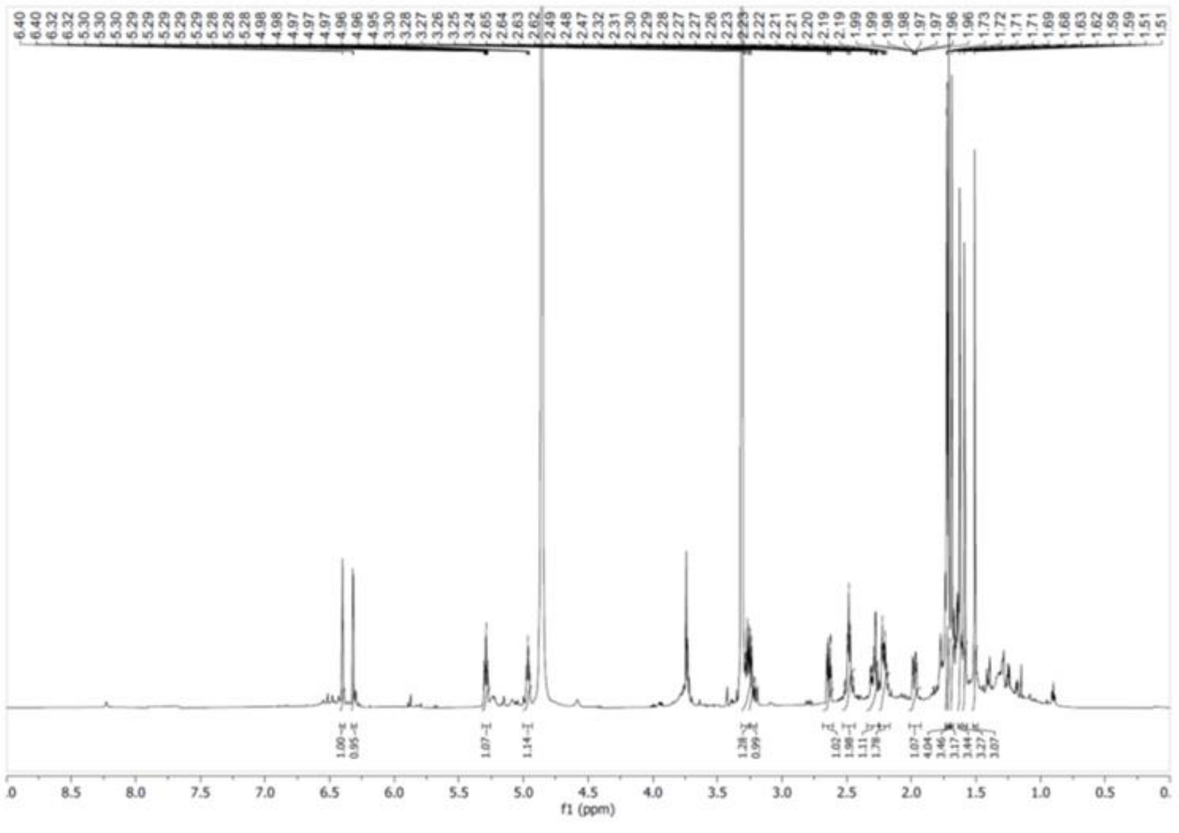

Supplementary Figure S61. ^{1}H NMR spectrum of compound **10** in CD_3OD at 600 MHz.

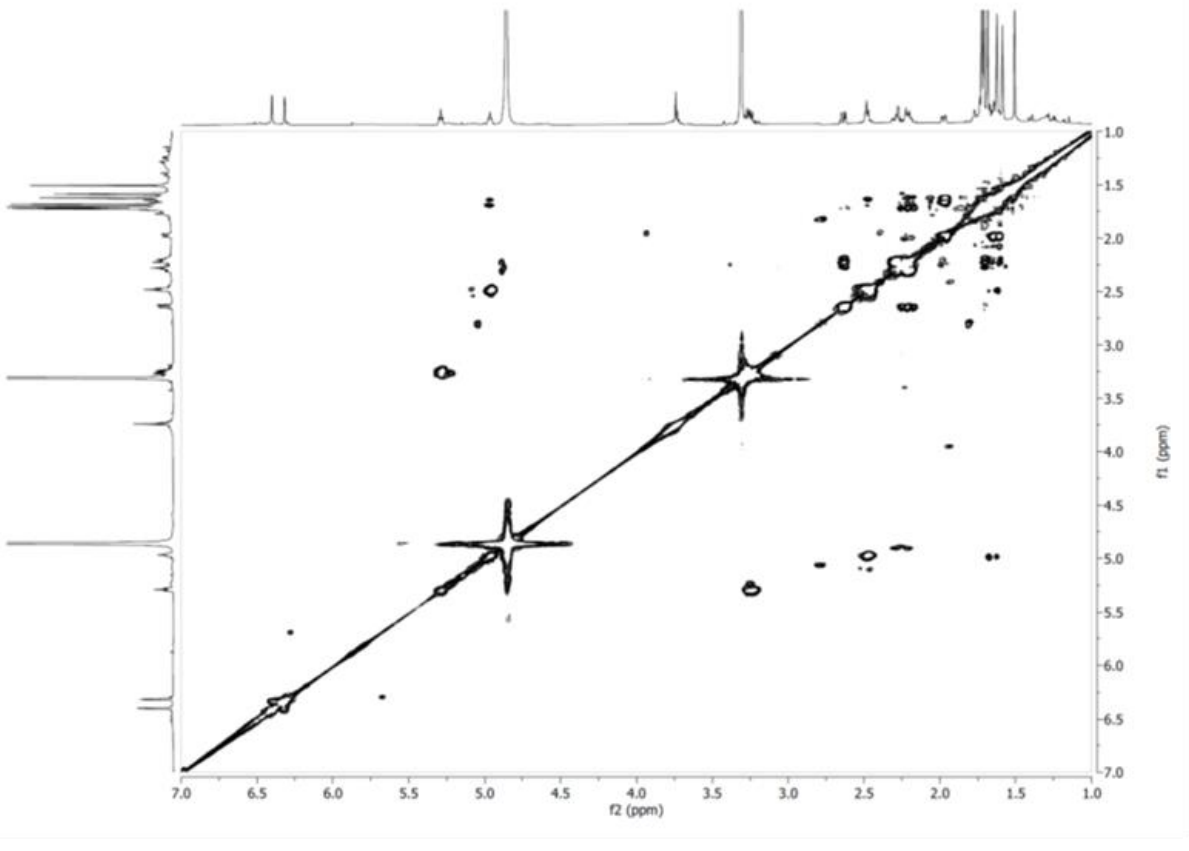

Supplementary Figure S62. COSY NMR spectrum of compound **10** in CD_3OD.

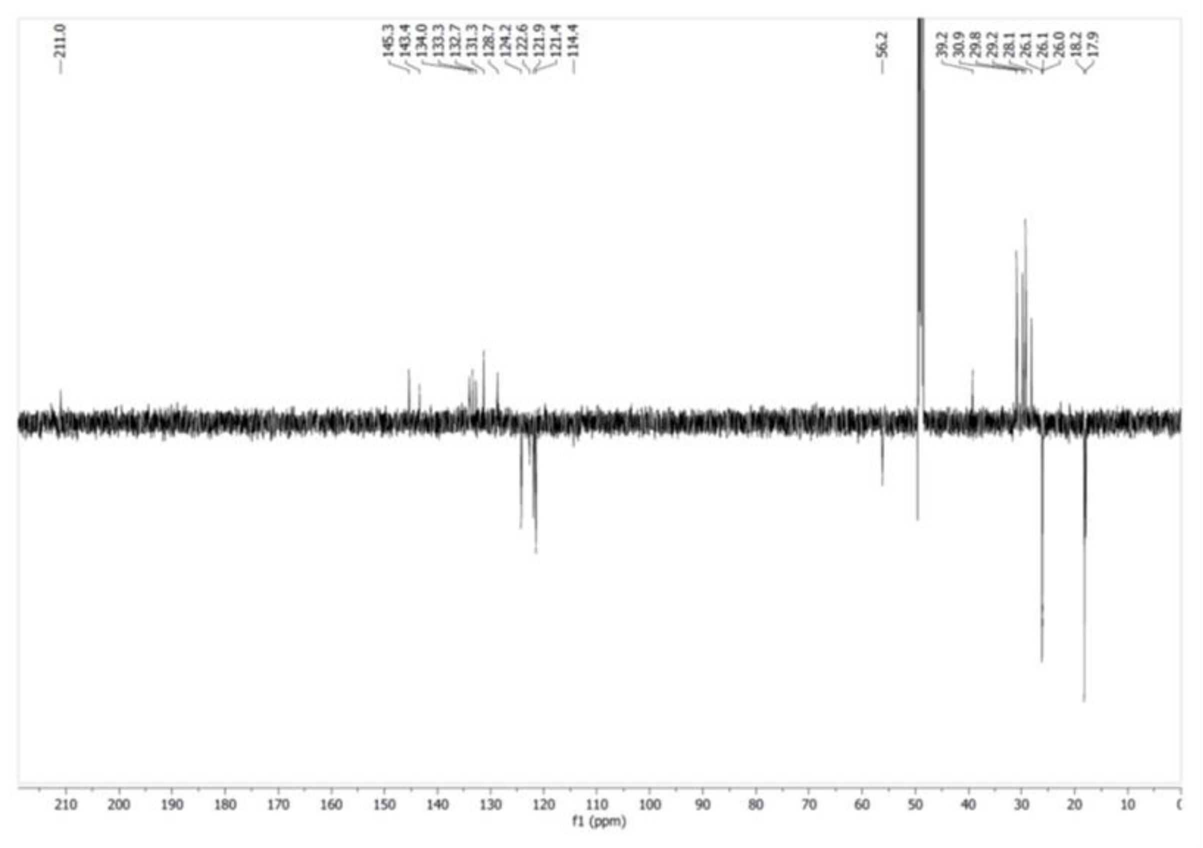

Supplementary Figure S63. ^{13}C-DEPTQ NMR spectrum of compound **10** in CD_3OD at 151 MHz.

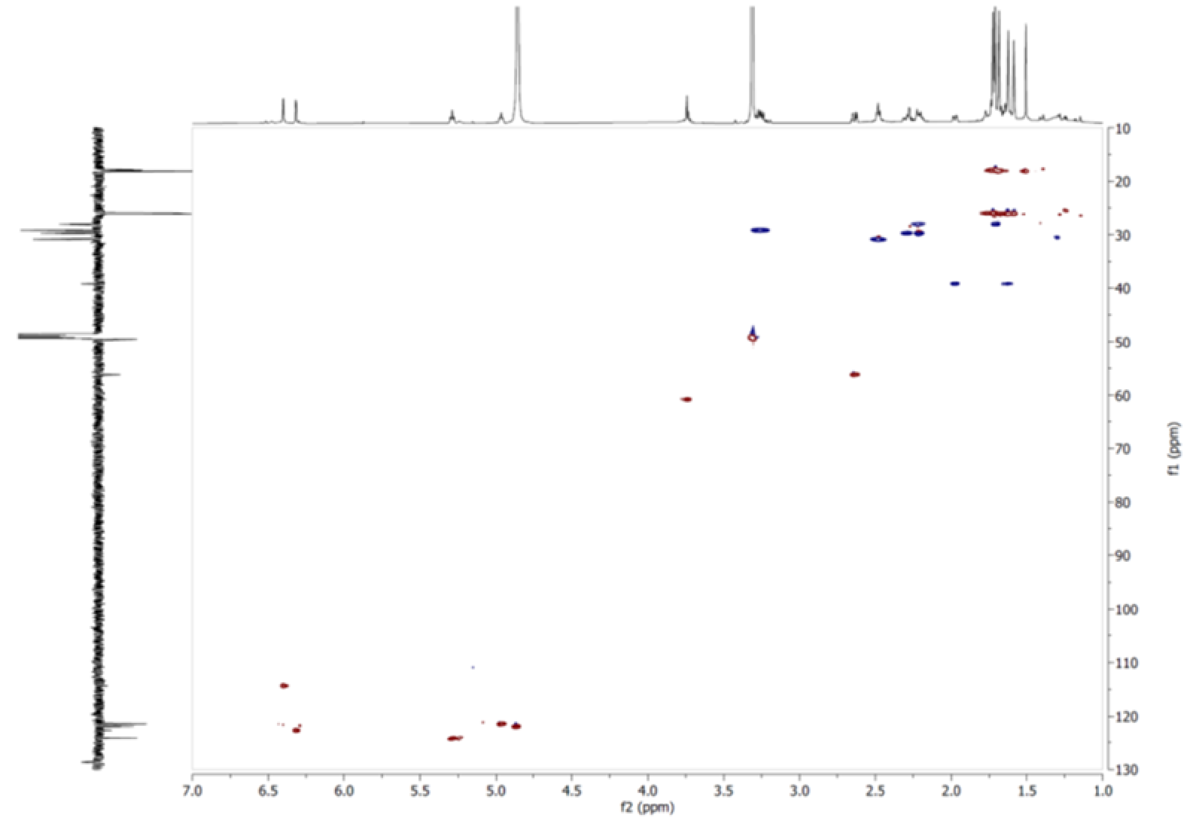

Supplementary Figure S64. Edited HSQC NMR spectrum of compound **10** in CD_3OD.

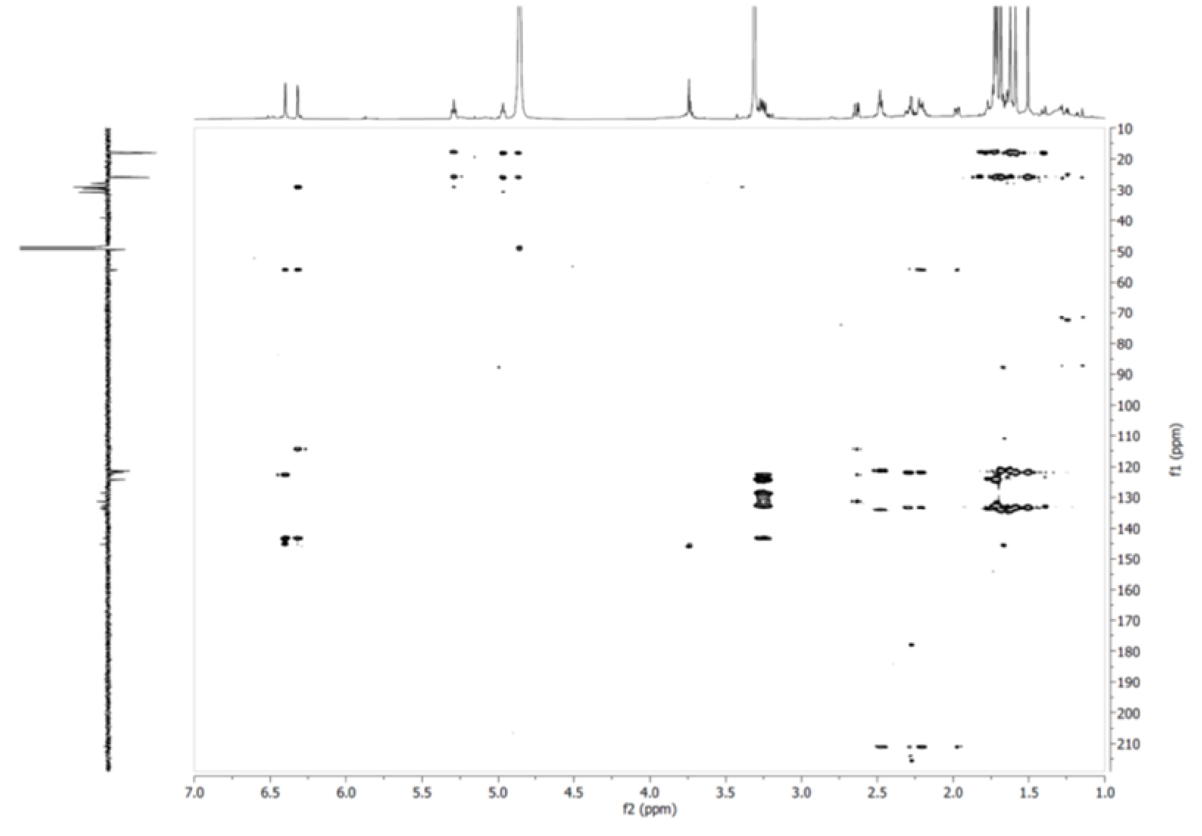

Supplementary Figure S65. HMBC NMR spectrum of compound **10** in CD_3OD.

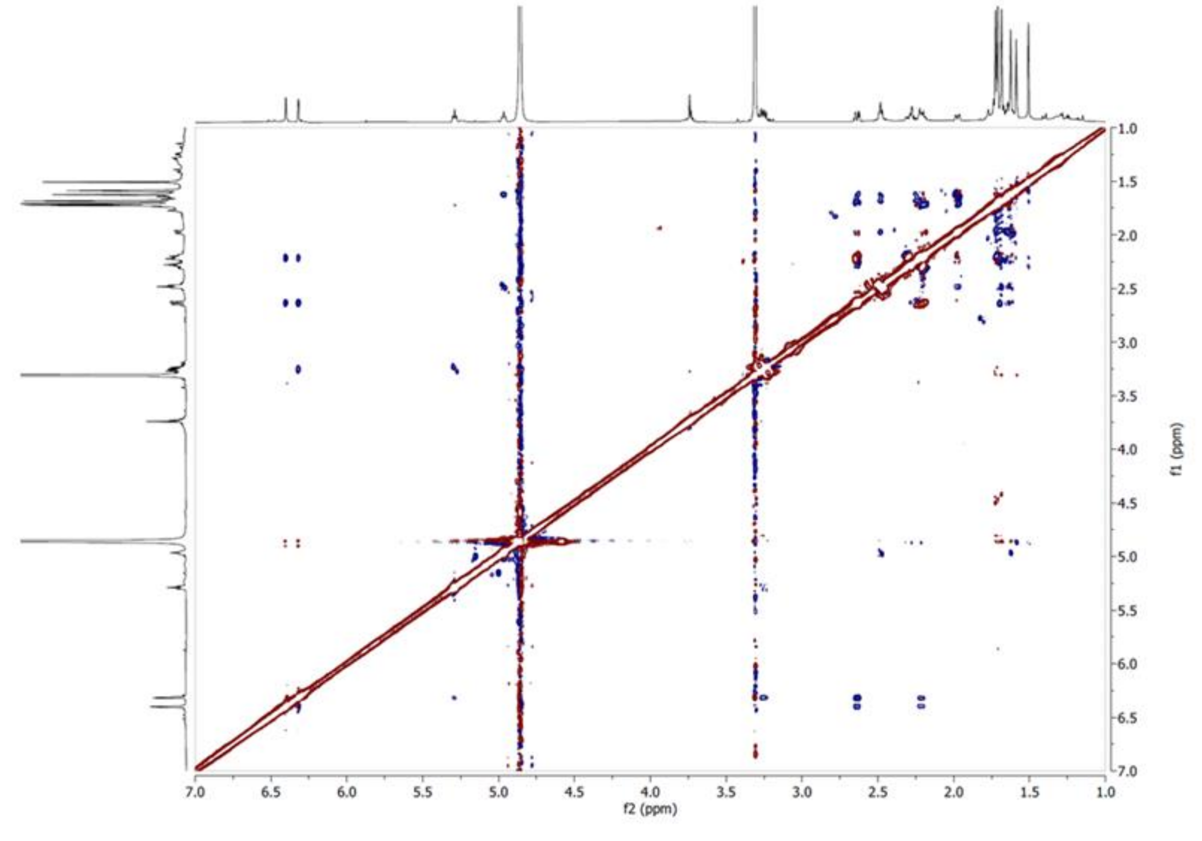

Supplementary Figure S66. ROESY NMR spectrum of compound **10** in CD_3OD.

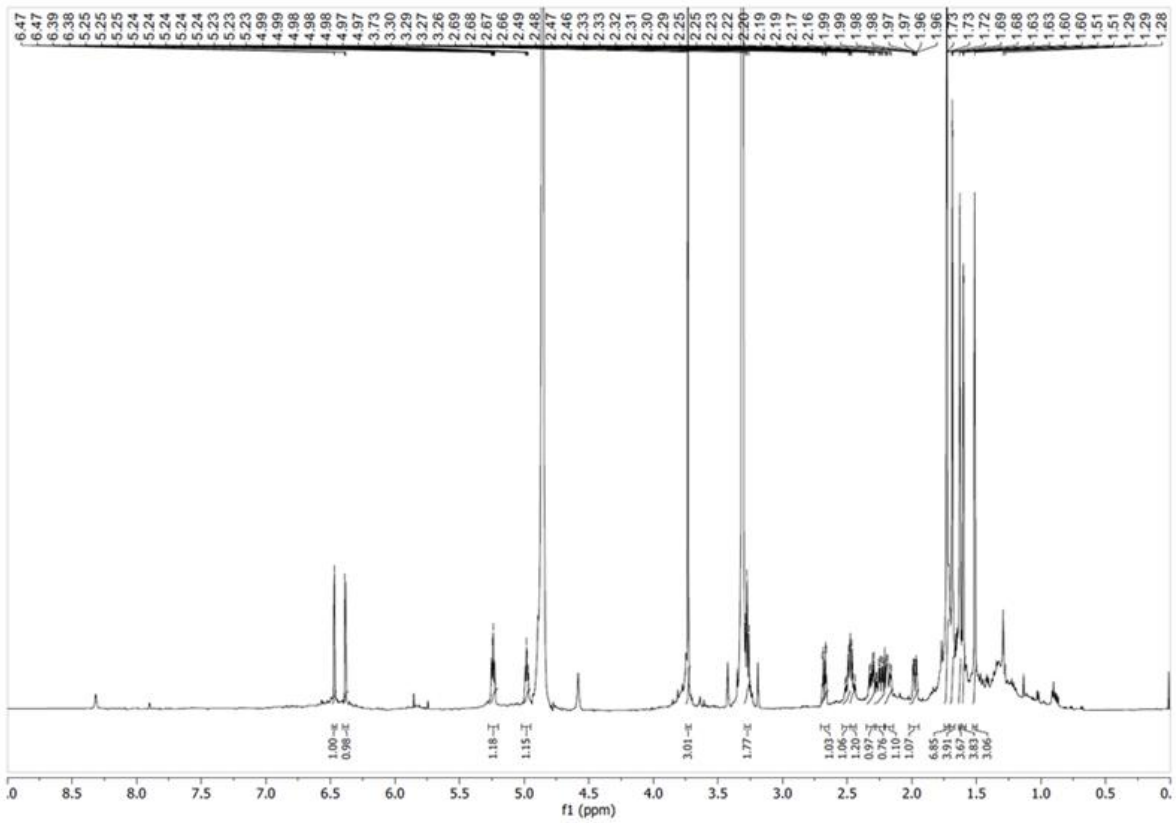

Supplementary Figure S67. ^{1}H NMR spectrum of compound **11** in CD_3OD at 600 MHz.

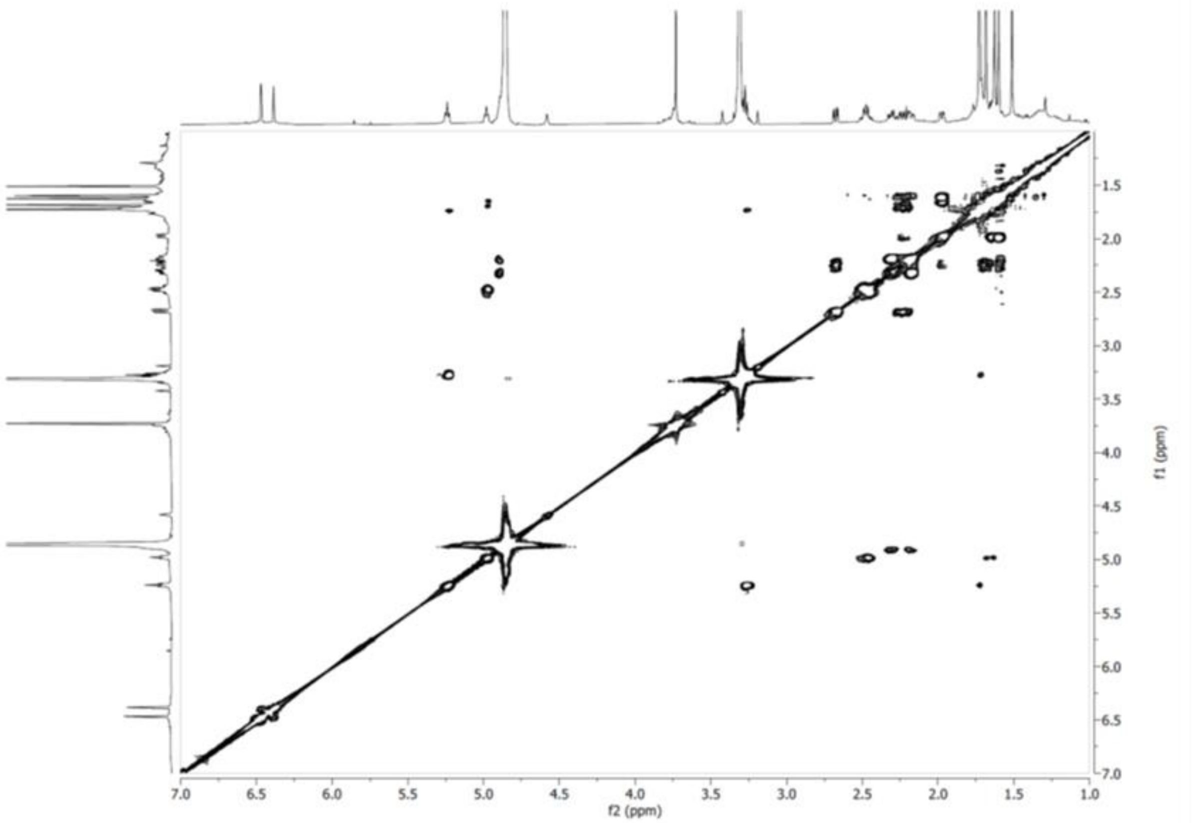

Supplementary Figure S68. COSY NMR spectrum of compound **11** in CD_3OD.

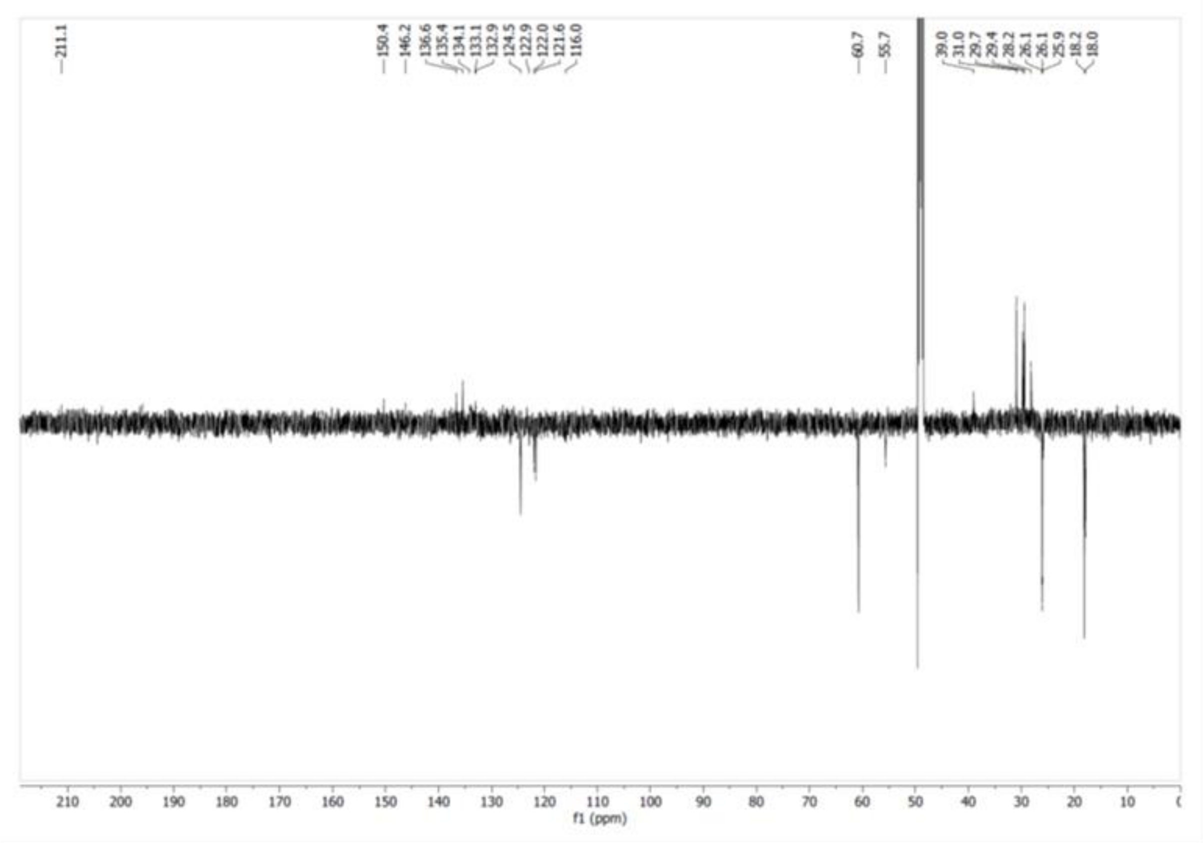

Supplementary Figure S69. ^{13}C-DEPTQ NMR spectrum of compound **1**1 in CD_3OD at 151 MHz.

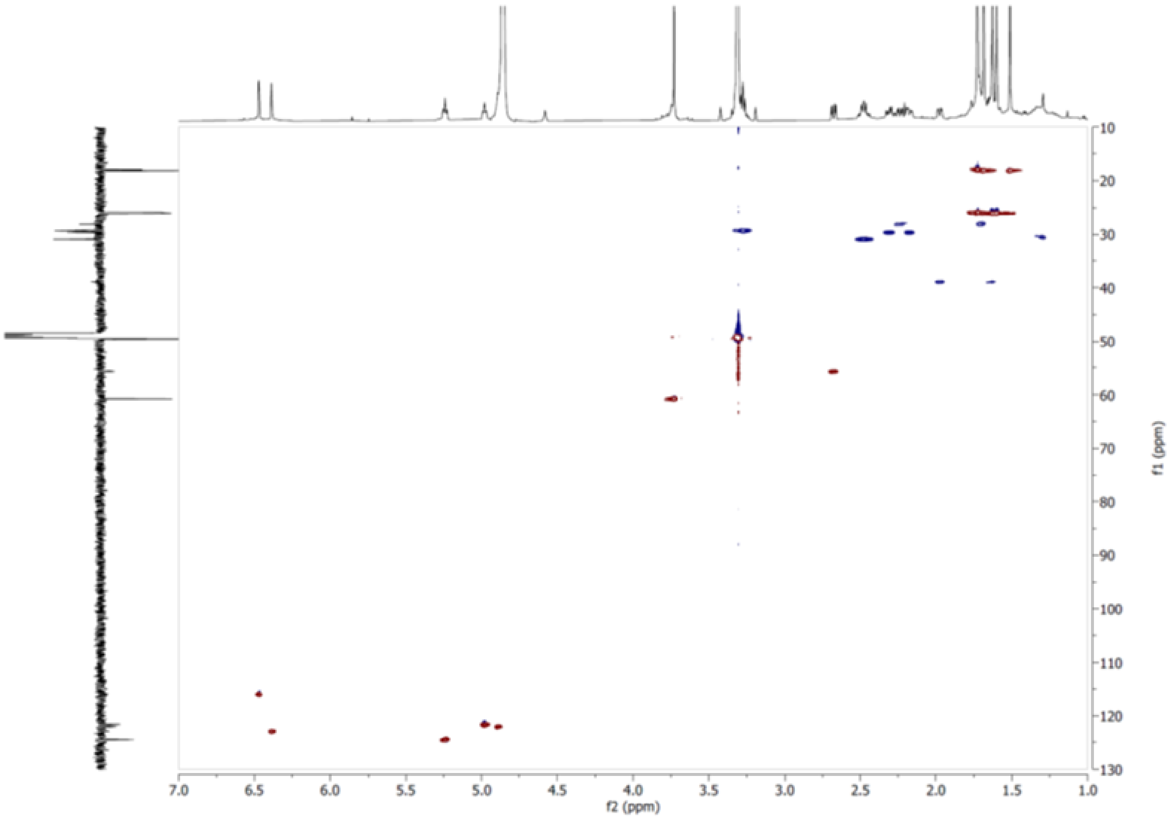

Supplementary Figure S70. Edited HSQC NMR spectrum of compound **1**1 in CD_3OD.

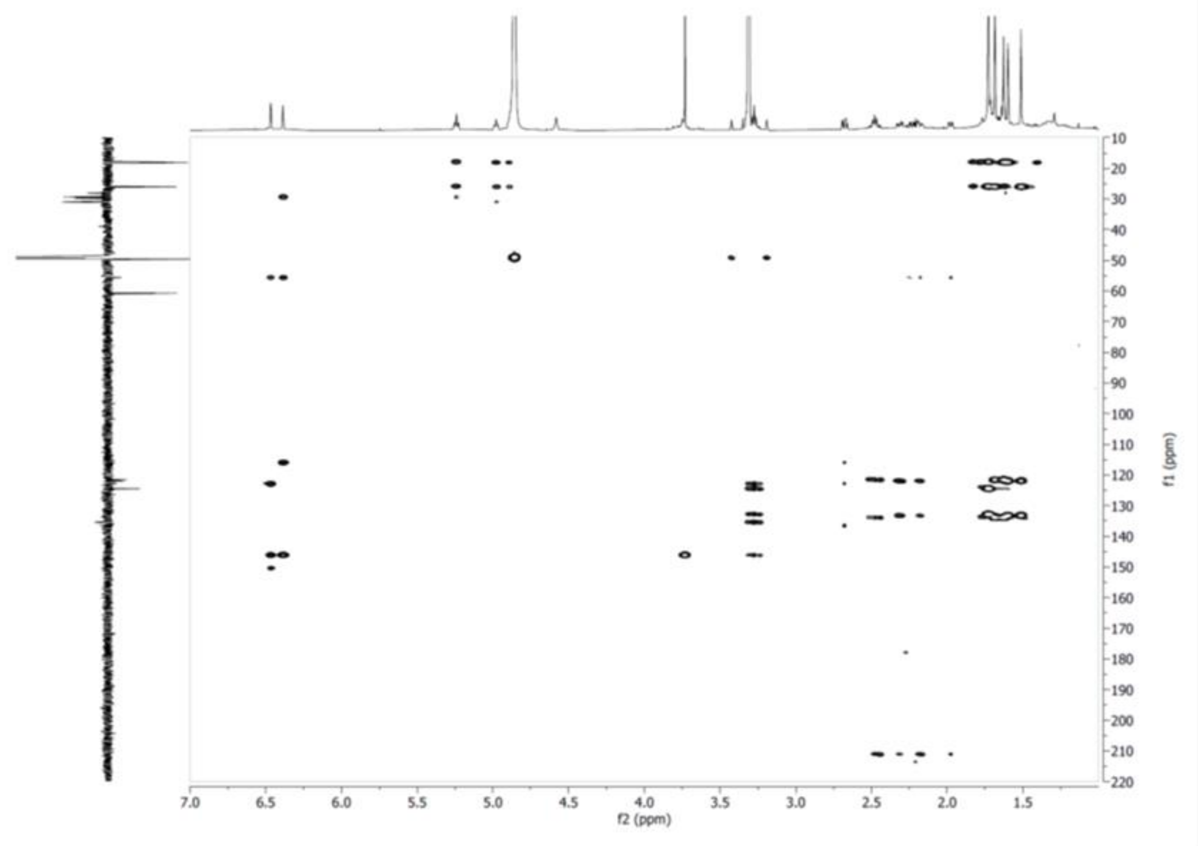

Supplementary Figure S71. HMBC NMR spectrum of compound **11** in CD_3OD.

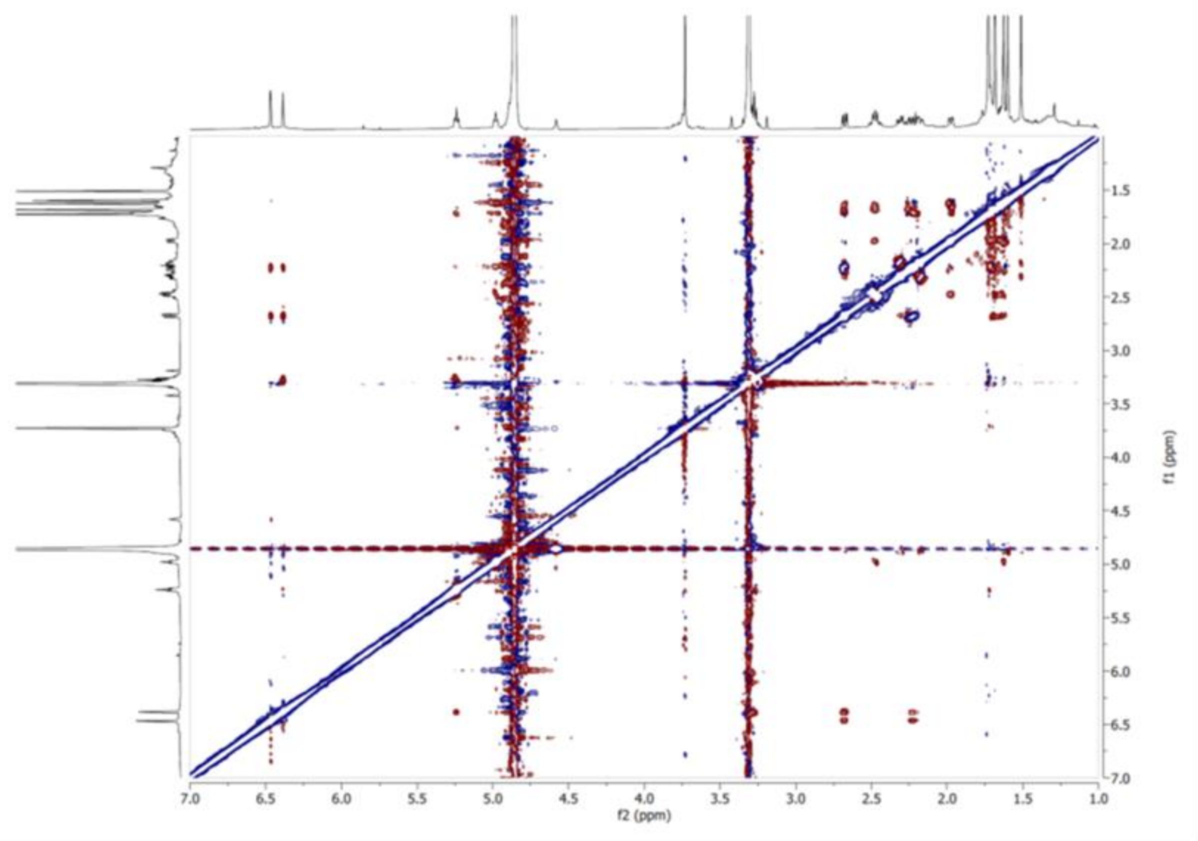

Supplementary Figure S72. ROESY NMR spectrum of compound **11** in CD_3OD.

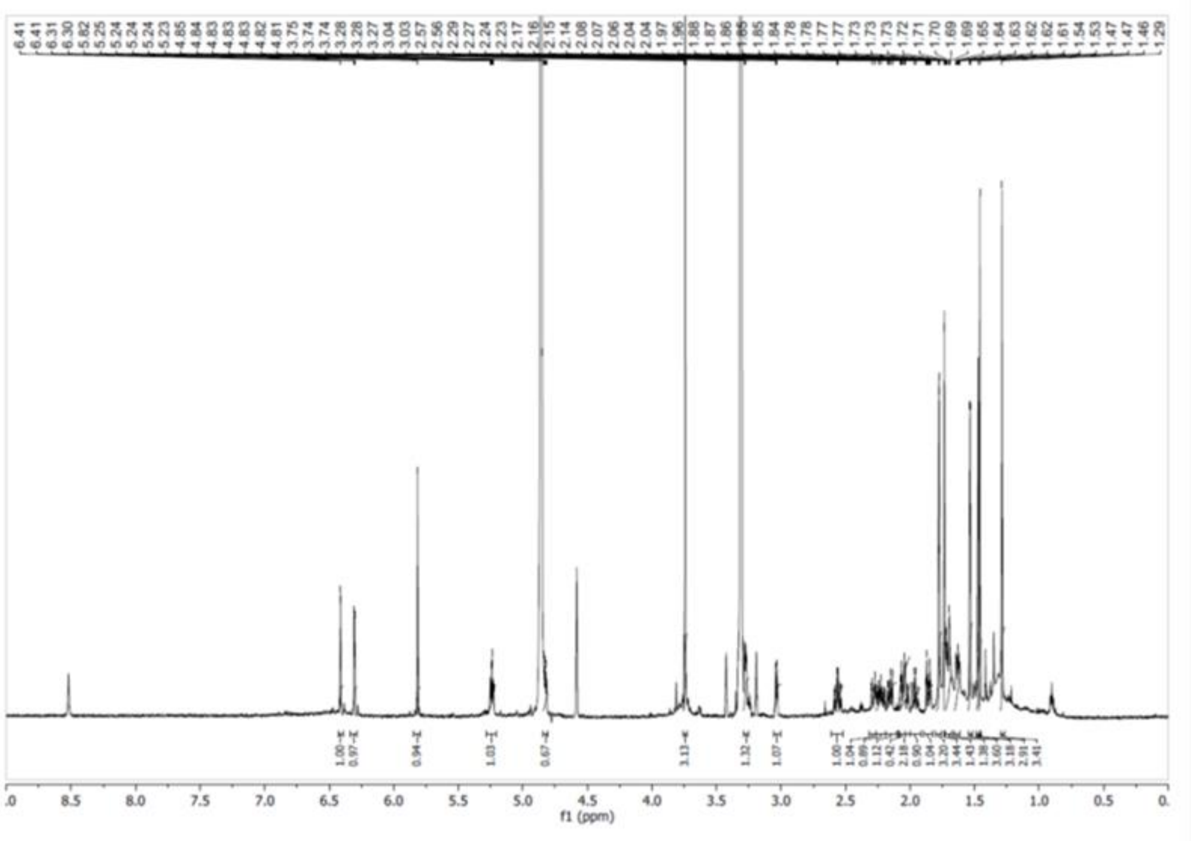

Supplementary Figure S73. ^{1}H NMR spectrum of compound **12** in CD_3OD at 600 MHz.

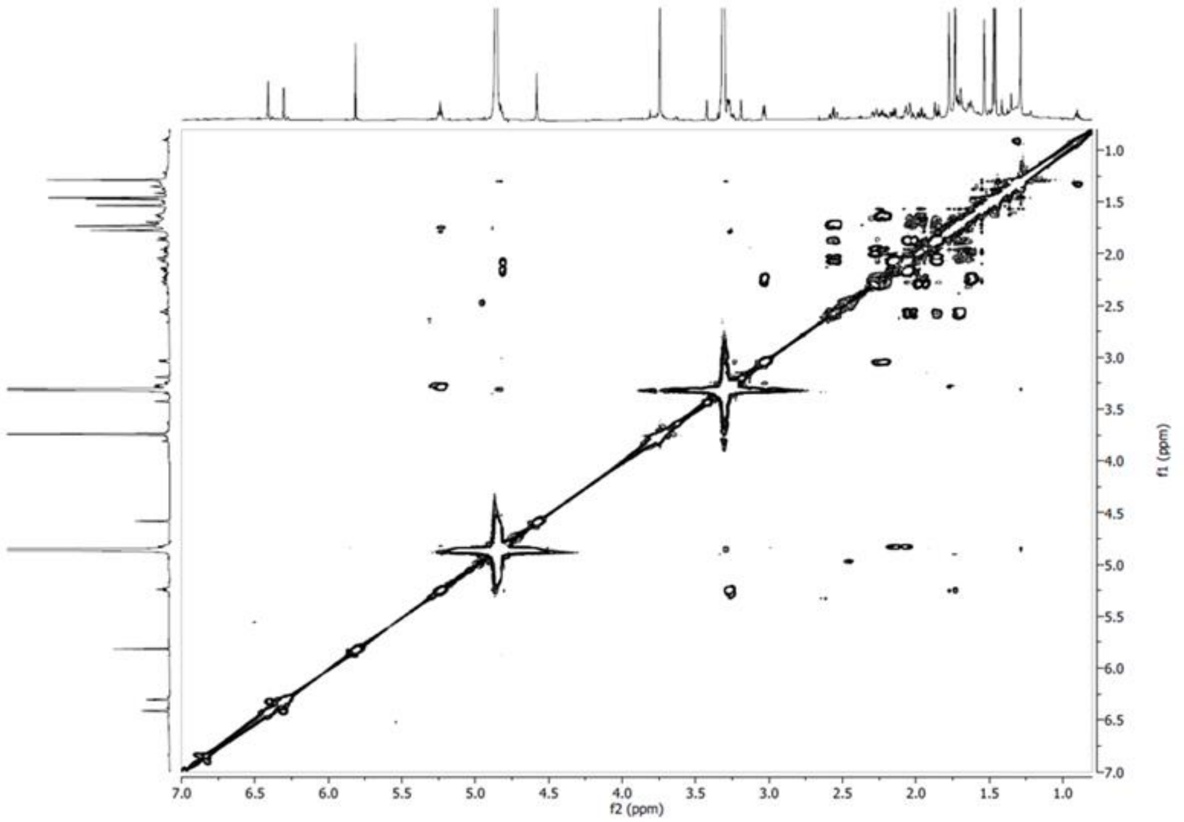

Supplementary Figure S74. COSY NMR spectrum of compound **12** in CD_3OD.

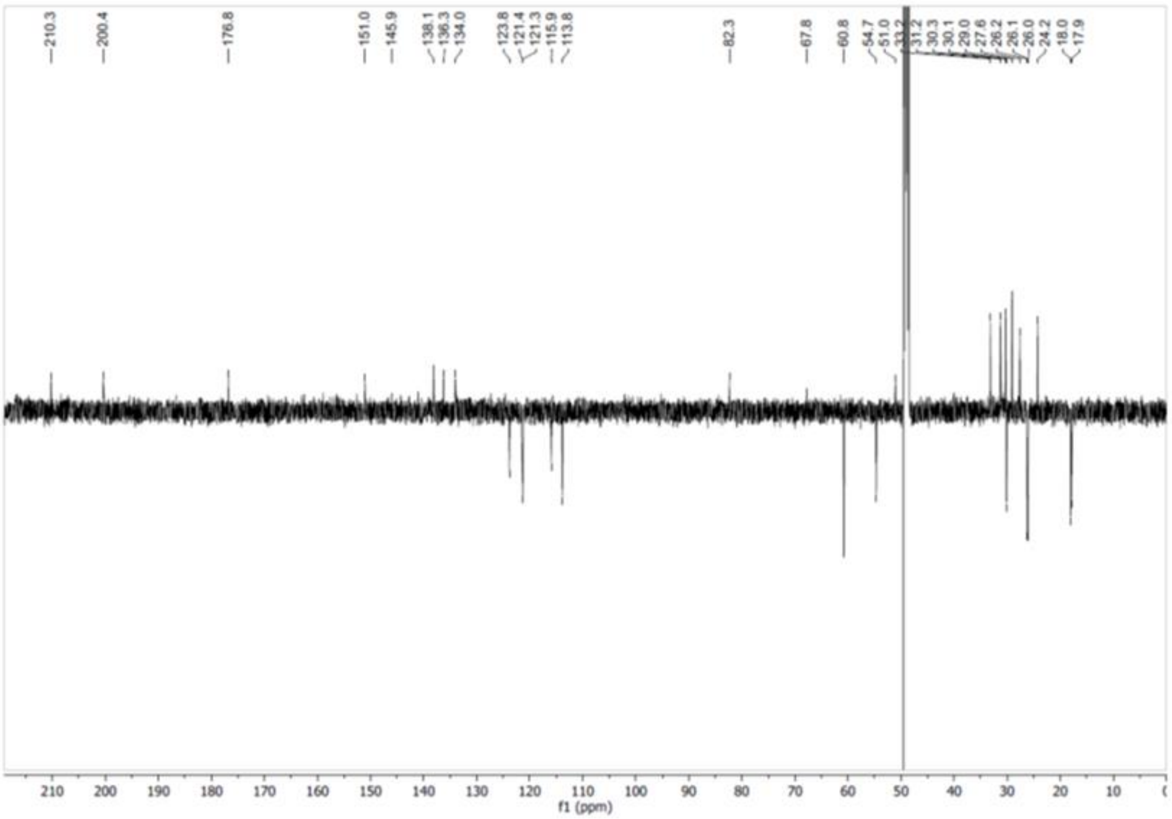

Supplementary Figure S75. ^{13}C-DEPTQ NMR spectrum of compound **12** in CD_3OD at 151 MHz.

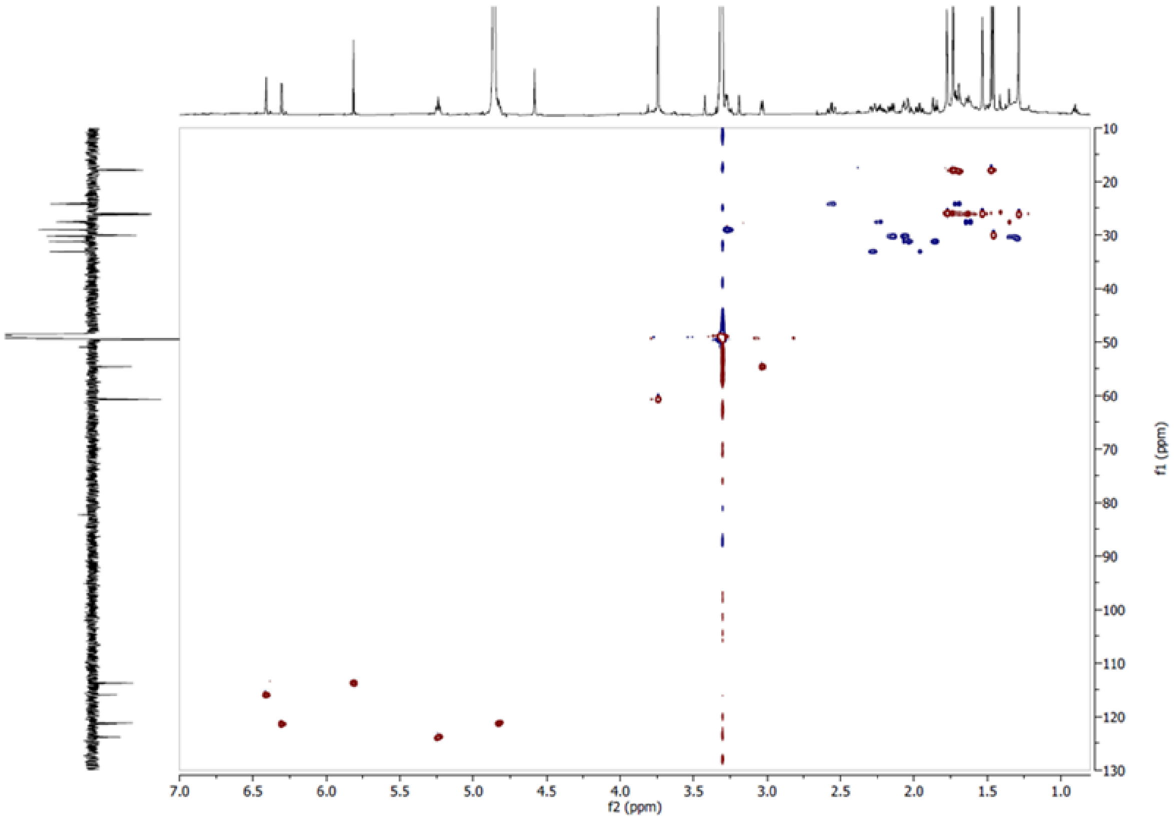

Supplementary Figure S76. Edited HSQC NMR spectrum of compound **12** in CD_3OD.

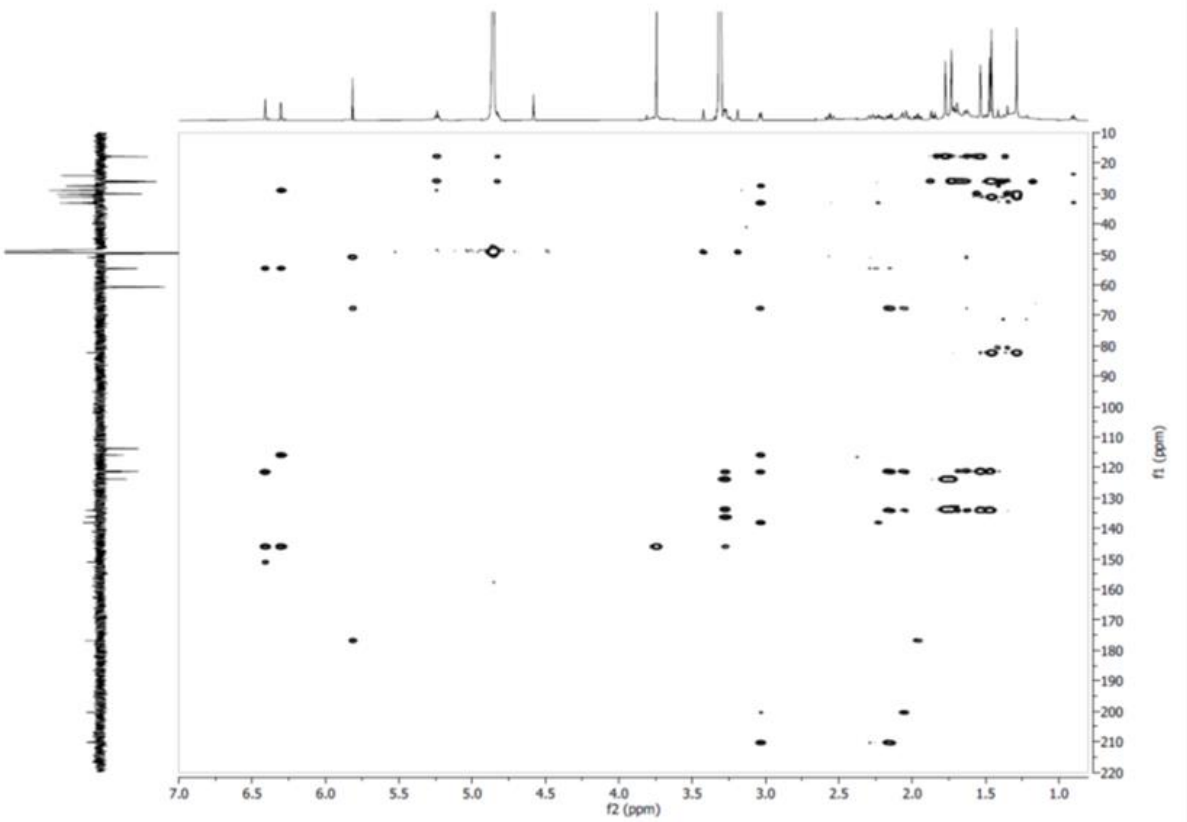

Supplementary Figure S77. HMBC NMR spectrum of compound **12** in CD_3OD.

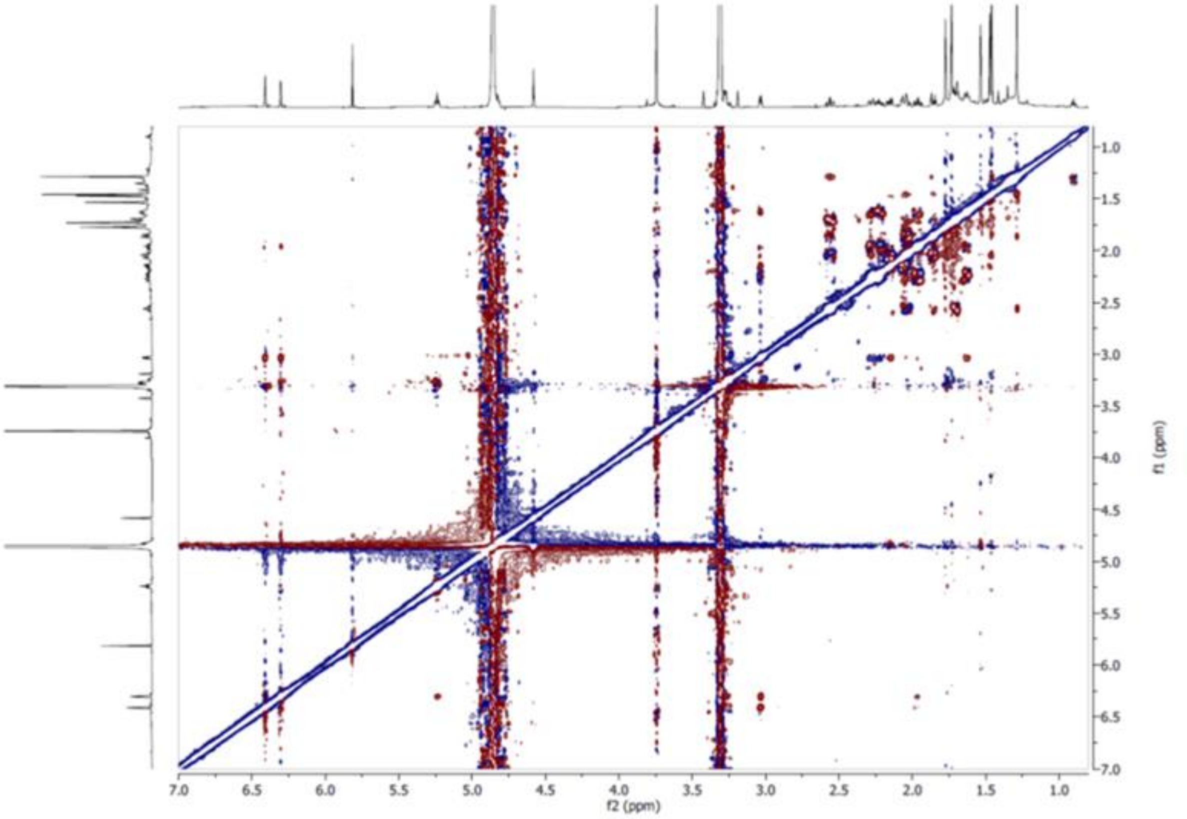

Supplementary Figure S78. ROESY NMR spectrum of compound **12** in CD_3OD.

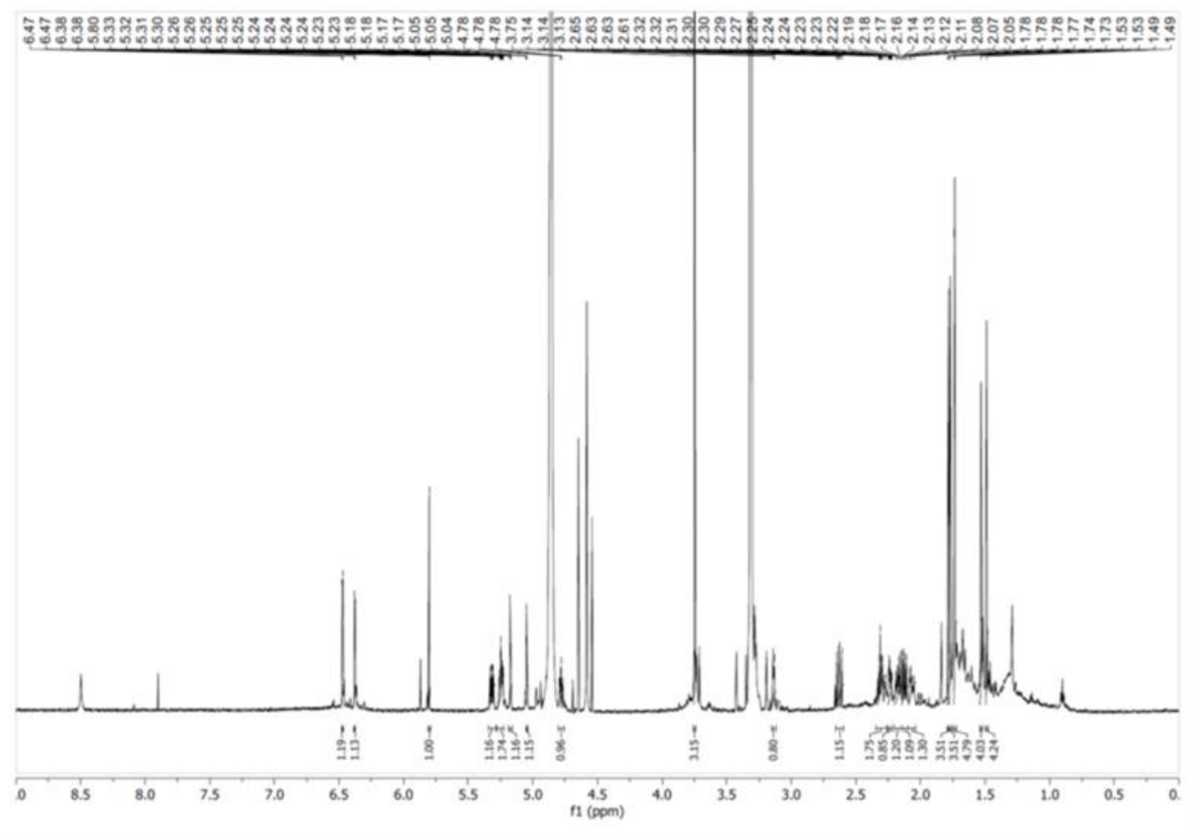

Supplementary Figure S79. ¹H NMR spectrum of compound **13** in CD_3OD at 600 MHz.

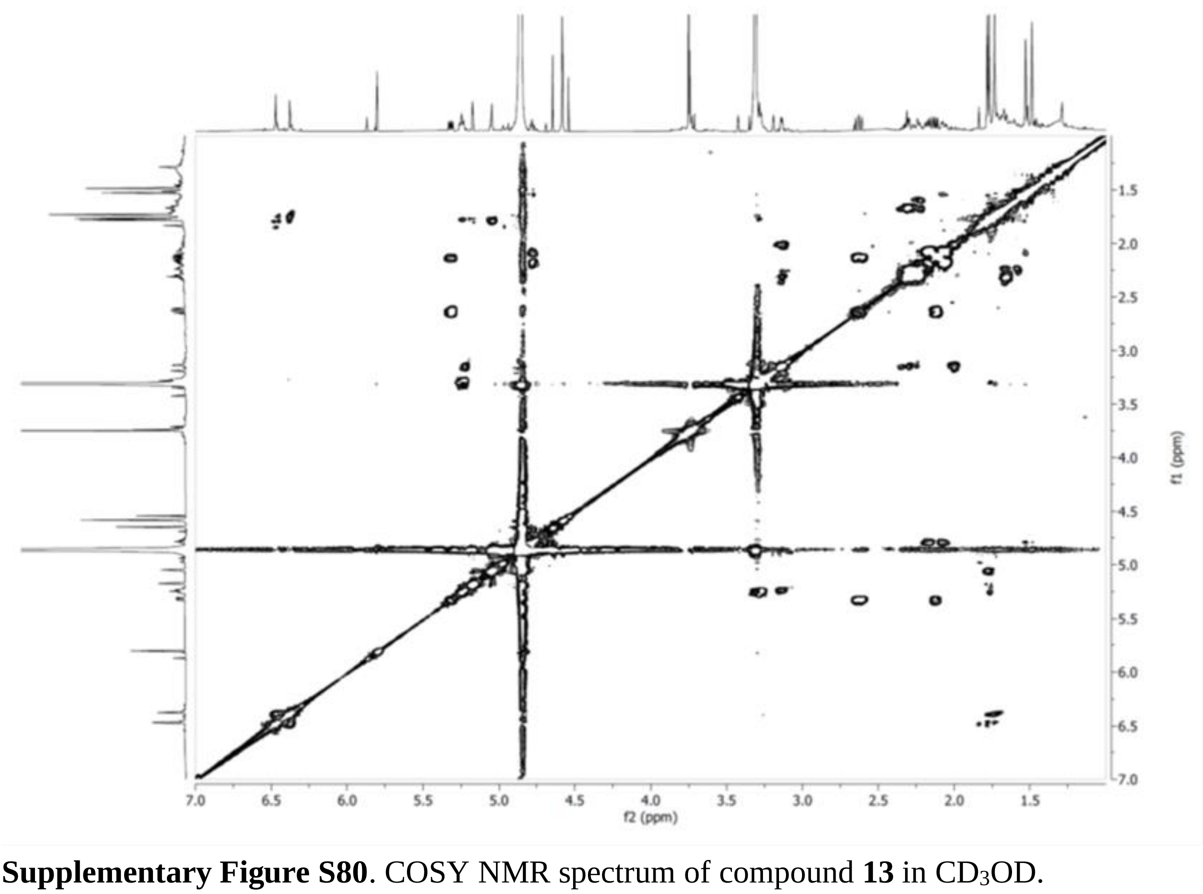

Supplementary Figure S80. COSY NMR spectrum of compound **13** in CD_3OD.

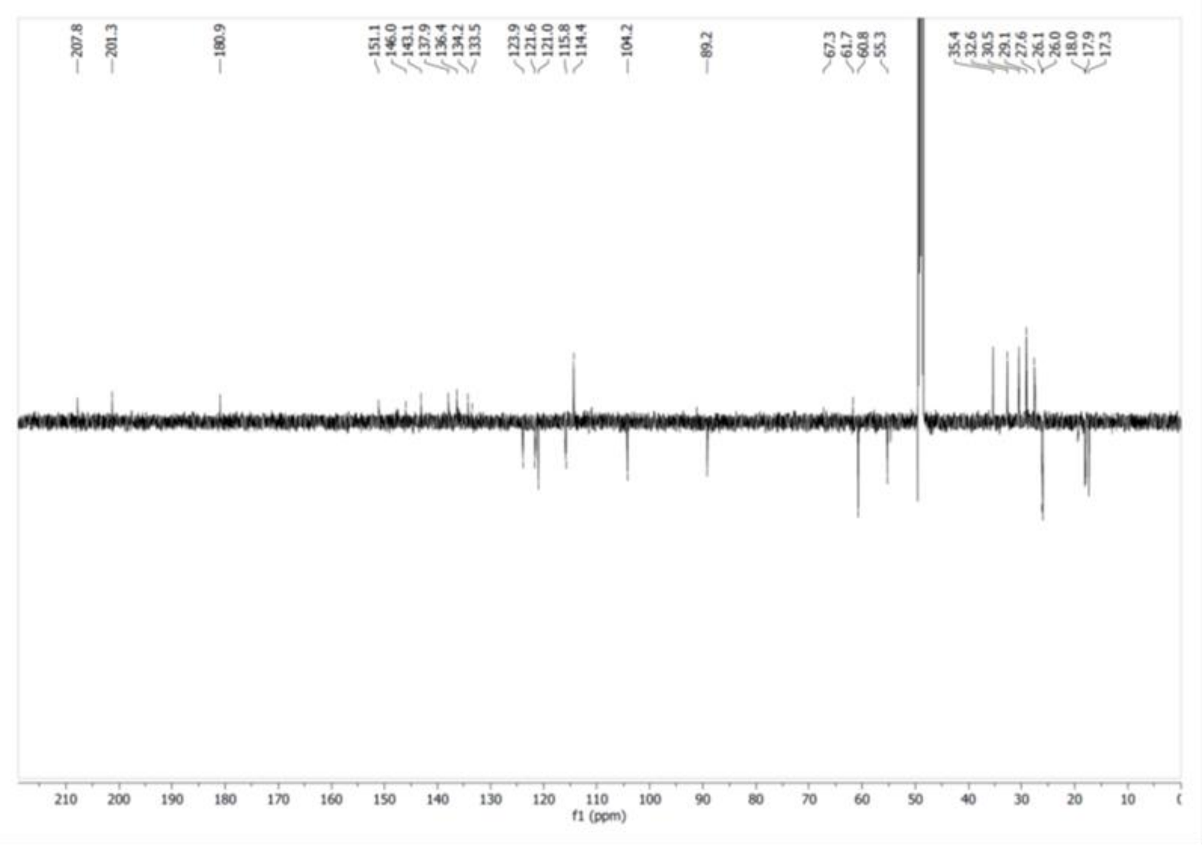

Supplementary Figure S81. ^{13}C-DEPTQ NMR spectrum of compound **13** in CD_3OD at 151 MHz.

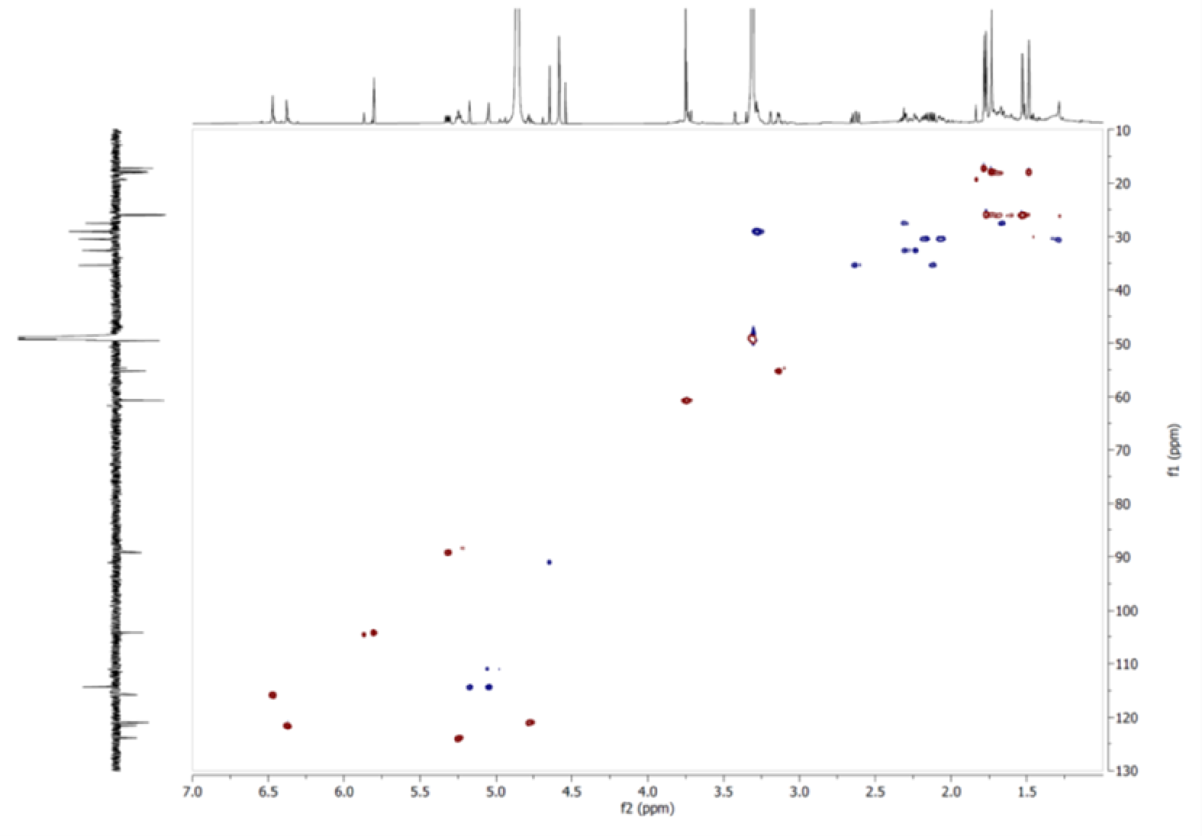

Supplementary Figure S82. Edited HSQC NMR spectrum of compound **13** in CD_3OD.

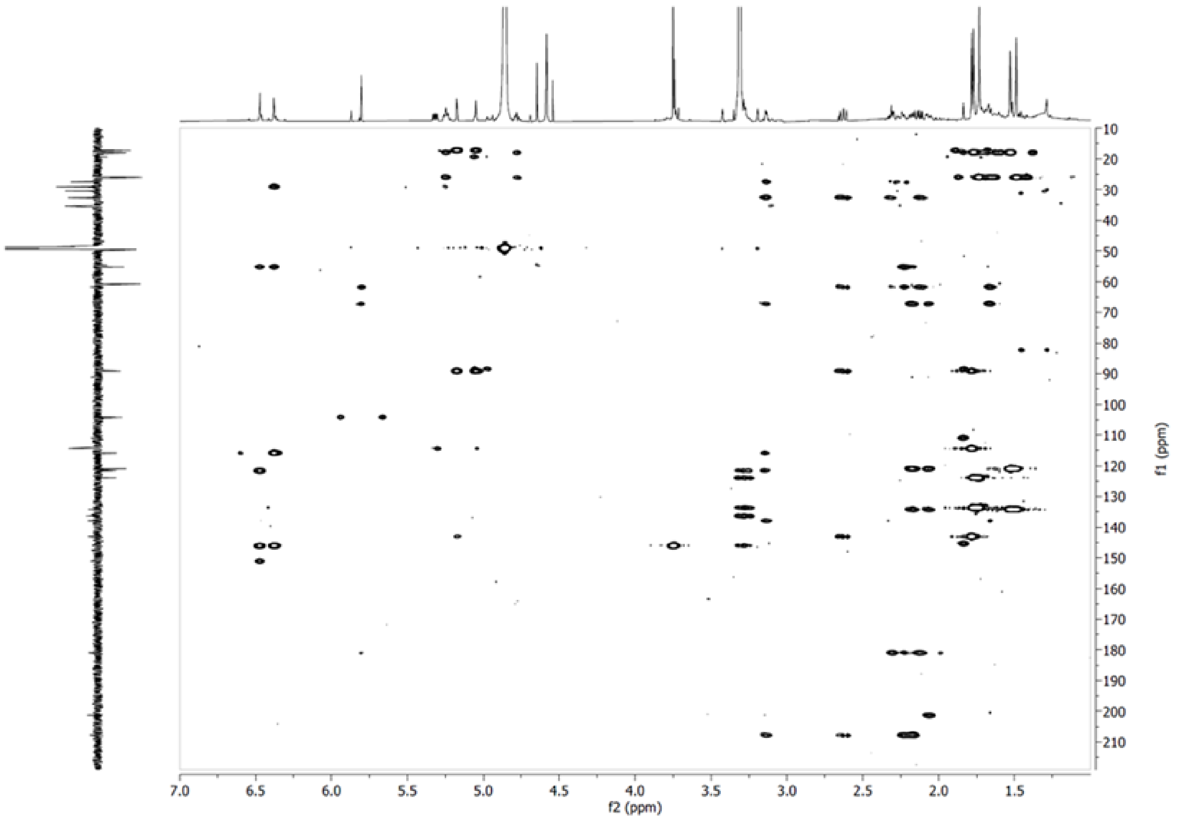

Supplementary Figure S83. HMBC NMR spectrum of compound **13** in CD_3OD.

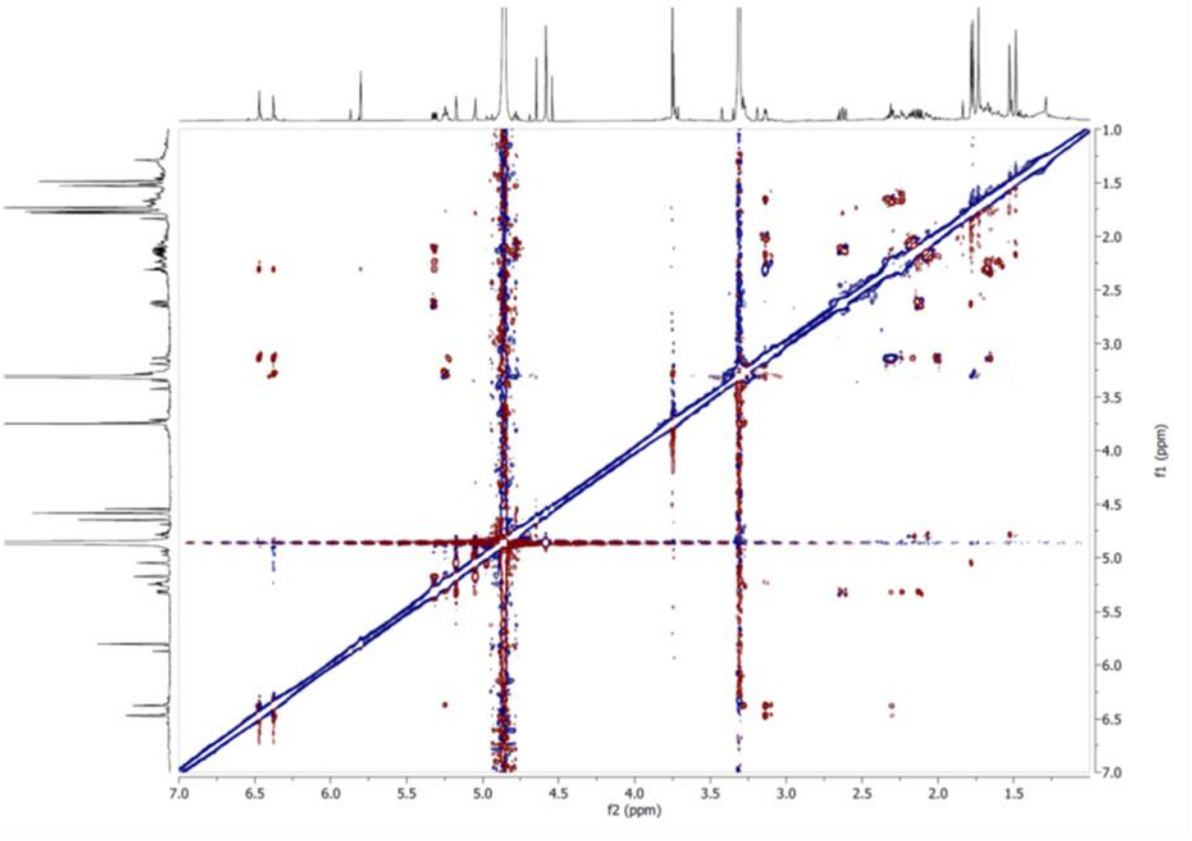

Supplementary Figure S84. ROESY NMR spectrum of compound **13** in CD_3OD.
